# Targeting Activin Receptor-like Kinase 2 Using Heterobifunctional Protein Degraders

**DOI:** 10.1021/acs.jmedchem.6c00714

**Published:** 2026-05-04

**Authors:** Daniel T. Webb, Katherine L. Jones, Natsuko Macabuag, Ruzica Bago, Joshua Betts, Sumit Bhattacharyya, Steve Clifton, Ryan A. J. Tinson, Chigozie Achara, Simon Gilbert, Stefanie Howell, David H. Drewry, Rebecca Rogers, Chris Jones, Kyle R. Ferguson, Alex N. Bullock, David M. Lindsay, William J. Kerr, William Esmieu

**Affiliations:** † Department of Pure and Applied Chemistry, University of Strathclyde, Glasgow G1 1XL, United Kingdom; ‡ 25913 Discovery from Charles River, Charles River, Chesterford Research Park, Saffron Walden CB10 1XL, United Kingdom; § Structural Genomics Consortium (SGC) and Division of Chemical Biology and Medicinal Chemistry, Eshelman School of Pharmacy and Lineberger Comprehensive Cancer Center, 15521 University of North Carolina at Chapel Hill, Chapel Hill, North Carolina 27599, United States; ∥ Division of Molecular Pathology and Division of Cancer Therapeutics, 5053 The Institute of Cancer Research, London SM2 5NG, United Kingdom; ⊥ Centre for Medicines Discovery, 105596 Nuffield Department of Medicine, University of Oxford, Oxford OX3 7FZ, United Kingdom

## Abstract

Activin receptor-like kinase 2 (ACVR1/ALK2) regulates bone morphogenetic protein signaling, and ALK2 modulation has been identified as a promising therapeutic strategy for conditions including fibrodysplasia ossificans progressiva (FOP), diffuse intrinsic pontine glioma (DIPG), and glioblastoma. Herein, we report on the development of first-in-class ALK2 degraders, including **M4K3233** (**13**), a potent and selective compound that was utilized as a chemical tool to study the mechanism of ALK2 degradation. Subsequent optimization of this compound resulted in **M4K3250** (**20**), a compound with improved ALK2 degradation potency. The compounds described have utility for studying the role of ALK2 in human disease and possess translational potential in drug discovery.

## Graphical abstract

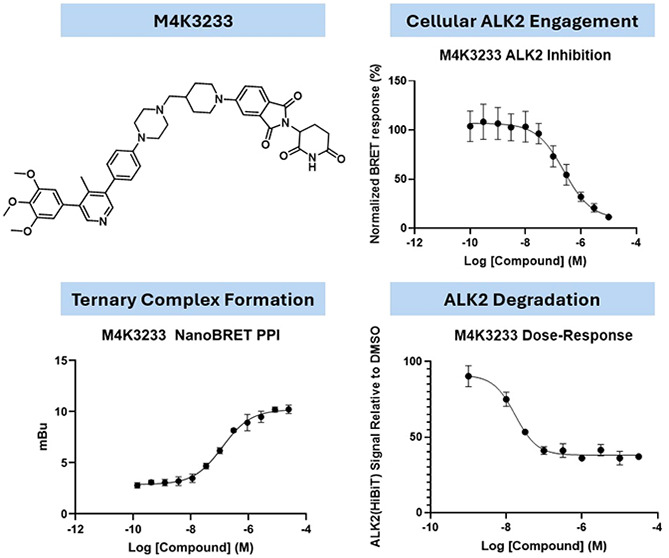

## Introduction

Activin Receptor-like Kinase 2 (ACVR1/ALK2) is a bone morphogenetic protein (BMP) type- I receptor, encoded by the gene *ACVR1*. ALK2 is one of seven BMP type-I receptors that are involved in the transforming growth factor-β (TGF-β)/BMP signaling pathways.^1,2^ BMP type-I receptors are transmembrane proteins, consisting of an extracellular region that contains the BMP ligand binding domain, a single transmembrane helix, a juxtamembrane region rich in glycine and serine residues (GS-region), and an intracellular serine/threonine kinase domain.^3^ BMP ligand binding regulates the formation of a heterotetrameric signaling complex consisting of two BMP type-I and two BMP type-II receptors.^4^ Trans-phosphorylation of the type-I receptor, within the GS-region, by the constitutively active type-II receptor leads to kinase activation. Upon activation, ALK2 phosphorylates the receptor-regulated SMAD proteins (R-SMADs) SMAD1/5/8, which then associate with the comediator protein SMAD4, and this protein complex is translocated into the nucleus where it regulates the expression of genes such as inhibitor of DNA binding 1 (ID1).^2,5^

Activating mutations within ALK2 have been identified as a genetic cause of the rare and devastating autosomal disorder fibrodysplasia ossificans progressiva (FOP), which is characterized by progressive heterotopic ossification (HO).^6,7^ The worldwide prevalence of FOP is approximately one in 1 million, the median estimated lifespan of patients is approximately 56 years, and to date only one drug, the retinoic acid receptor γ agonist, palovarotene, has been approved by the FDA for this condition.^8–11^ ALK2 inhibition is considered a promising therapeutic strategy for the treatment of FOP and, at present, there are three ALK2 inhibitors; **fidrisertib** (**1**), **saracatinib** (**2**), and **zilurgisertib** (**3**) in phase 2 clinical trials for FOP (NCT05039515, NCT04307953, and NCT05090891, respectively) (Figure 1).^12–15^ **Fidrisertib** (**1**) and **zilurgisertib** (**3**) were specifically developed to inhibit ALK2, whereas **saracatinib** (**2**) is a rather promiscuous kinase inhibitor that was initially developed by scientists at AstraZeneca, as a dual inhibitor of SRC/ABL, and was studied extensively in the clinic as a cancer therapy.^16^ It was subsequently identified that saracatinib is a potent inhibitor of ALK2 and, thus, presented a promising candidate for drug repurposing studies in FOP disorder.

**Figure 1:**
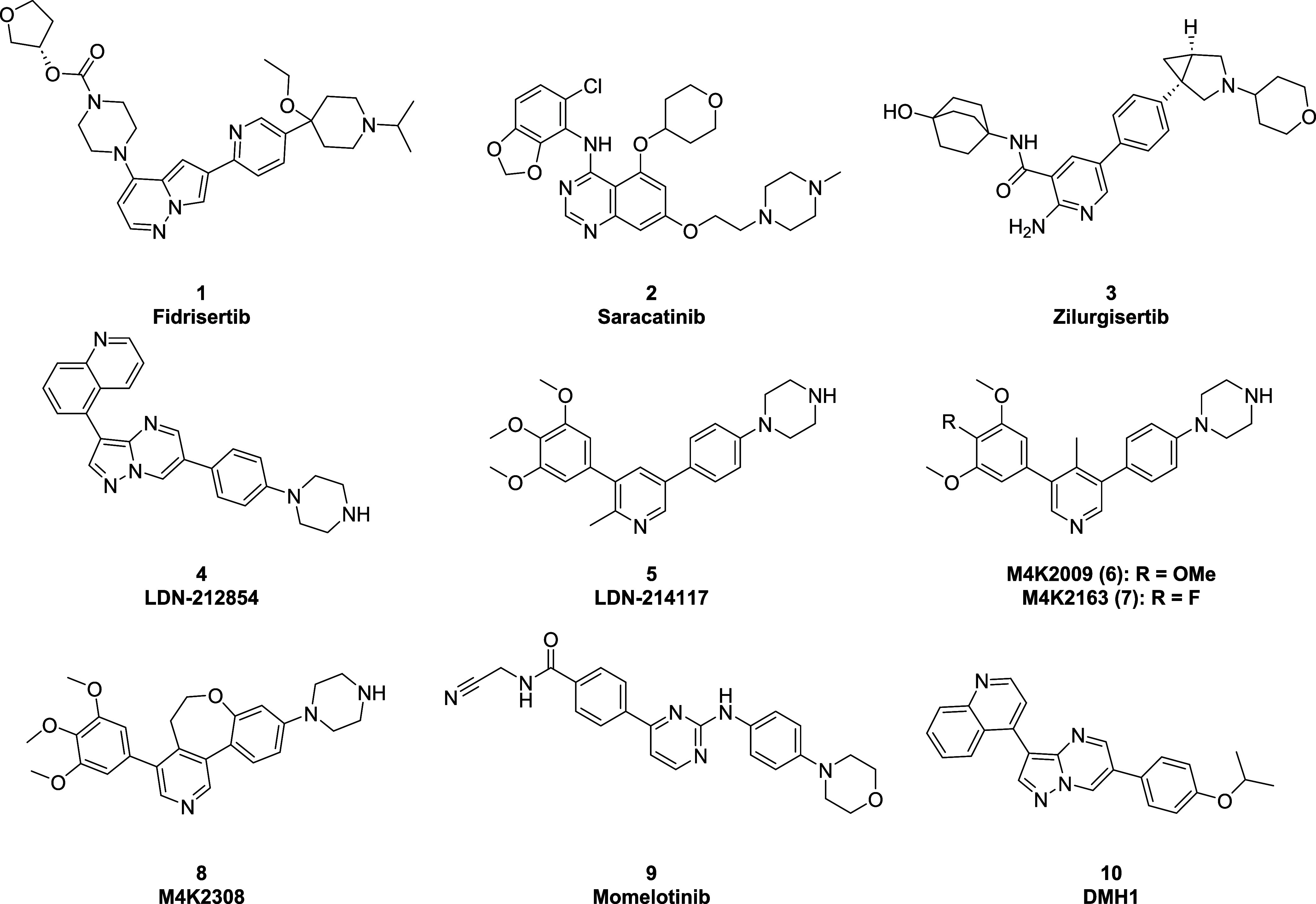
Chemical structures of selected ALK2 inhibitors.

Gain-of-function mutations in the *ACVR1* gene have also been identified in approximately 25% of diffuse intrinsic pontine glioma (DIPG) cases.^17^ DIPGs are grade IV pediatric brain tumors that account for approximately 10% of pediatric brain cancers. The five-year overall survival rate for this disease currently stands at <1% and there are no FDA-approved chemotherapeutic strategies for the treatment of DIPG.^18–20^ Studies by Carvalho et al. have shown that treatment of patient-derived DIPG cell lines with the preclinical stage ALK2 inhibitors, **LDN-212854** (**4**) and **LDN-214117** (**5**), inhibits BMP signaling.^21^ Furthermore, ALK2 inhibition was shown to induce apoptosis in HSJD-DIPG-007 cells, harboring ALK2 R206H mutations, extending survival in orthotopic patient-derived xenograft models of DIPG.^21^ Subsequent optimization of **LDN-214117** (**5**) has resulted in the identification of ALK2 inhibitors such as **M4K2009** (**6**), **M4K2163** (**7**), and **M4K2308** (**8**), which are being evaluated preclinically as potential therapeutics for DIPG (Figure 1).^22,23^

BMP signaling has been shown to play a central role in mediating hepcidin transcriptional induction in hepatocytes, which is associated with the development of anemia in myelofibrosis patients.^24^ **Momelotinib** (**9**) is a JAK1/JAK2/ALK2 inhibitor that is approved for the treatment of myelofibrosis patients with anemia. Its ability to suppress hepcidin expression and restore erythropoiesis in patients has been attributed to its ability to inhibit both BMP and JAK/STAT3 signaling pathways.^25^ Recent studies have also shown that BMP signaling is growth-promoting in glioblastoma cells and that treatment of U87 glioblastoma cells with the selective BMP type-1 receptor inhibitor **DMH1** (**10**) resulted in a decrease in ID1 expression, and impaired cell viability (Figure 1).^26^

Targeted protein degradation (TPD) is an emerging drug discovery approach that harnesses the body’s endogenous protein degradation pathways. Heterobifunctional compounds can be used to degrade disease-causing proteins by recruiting an E3 ubiquitin ligase and bringing it into proximity of the target protein. This can result in the poly ubiquitination of the target protein, which marks it for degradation by the proteasome or, less frequently, the lysosome.^27–29^ Protein degradation by heterobifunctional molecules occurs via an event-driven mechanism of action (MOA), in contrast to the occupancy-driven MOA typically seen with small molecule inhibitors.^30,31^

ALK2 regulation is a validated strategy with broad scope for therapeutic benefit; however, to date, most reported drug discovery efforts have focused on using ATP-competitive small molecule inhibitors (SMIs) to achieve this effect. We hypothesized that employing a TPD approach could have several benefits over the use of SMIs in the context of targeting ALK2. First, due to their event driven MOA, protein degraders can exhibit substoichiometric activity, which can result in enhanced efficacy over SMIs.^32^ Second, because the activity of bifunctional degraders depends on the formation of a ternary complex between two proteins, they may possess increased selectivity with respect to protein degradation; in contrast, achieving kinome-wide selectivity using SMIs has historically been challenging in drug discovery.^33,34^ Finally, protein degraders also target the nonenzymatic functions of proteins, and several kinases are known to have scaffolding roles in signaling pathways. As such, we believed that an ALK2 degrader would represent a highly useful chemical probe to study the potential noncatalytic functions of ALK2.^35^

## Results and Discussion

### Development of ALK2 Degraders

At the outset of this study, we selected **M4K2009** (**6**) as the ALK2 ligand to be incorporated into heterobifunctional degraders. **M4K2009** (**6**) is a potent and selective ALK2 inhibitor (ALK2 NanoBRET pIC_50_ = 7.3), which demonstrates good oral bioavailability (F = 100%) and CNS penetration (C_brain_/C_plasma_ at 4 h = 0.90) in mice.^22^ Analysis of the cocrystal structure of **M4K2009** (**6**) bound to ALK2 (PDB: 6SZM)^22^ showed that the ligand occupied the ATP-binding site with the aryl piperazine structural feature exposed to solvent and, as such, presented an attractive vector for the attachment of a linker and E3 ligase ligand (Figure 2a). In line with these considerations, we initially prepared two bifunctional compounds (**11** and **12**) by connecting **M4K2009** (**6**) to ligands for the E3 ligases, cereblon (CRBN) and Von Hippel-Lindau (VHL), respectively, using a flexible polyethylene glycol (PEG) linker of moderate chain length (Figure 2b).

**Figure 2:**
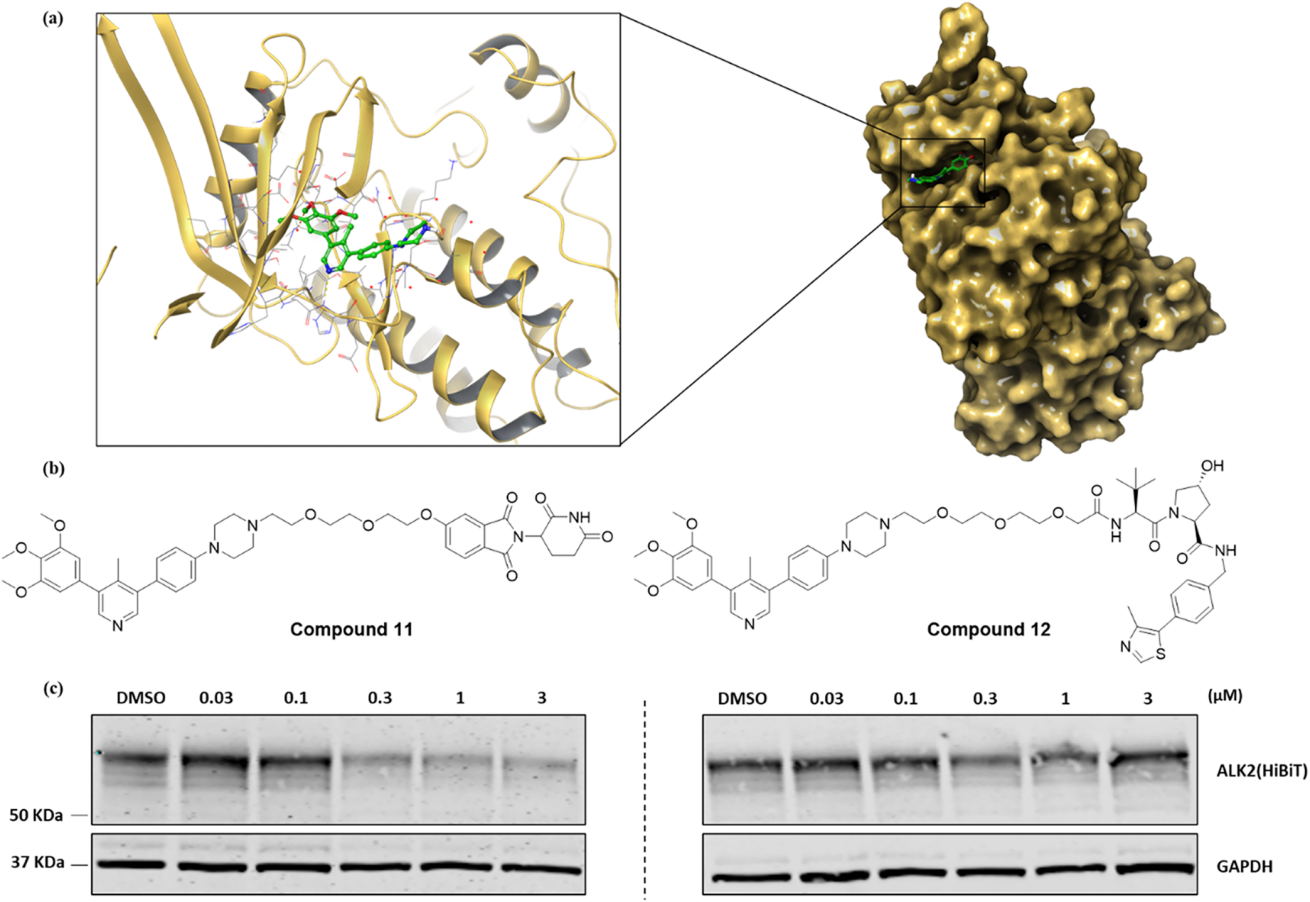
Approach to first generation ALK2 degraders. (a) Cocrystal structure of **M4K2009** (**6**) (green) bound to ALK2 (gold) (PDB: 6SZM).^22^ (b) Structures of first-generation ALK2 degraders. (c) ALK2-HiBiT transfected HEK-293 cells were treated for 24 h with increasing concentrations of each compound from 0.03 μM to 3 μM, then the cell lysates were analyzed by immunoblotting.

Initially, the ability of compounds **11** and **12** to engage ALK2 in cells was determined using a NanoBRET assay (Table 1, Figure S1). We found ALK2 to be a particularly challenging target from a biological evaluation standpoint due to its low expression levels across a range of cell lines. Furthermore, attempts to validate available ALK2 antibodies were unsuccessful. As such, a model system was employed whereby HEK-293 cells were transfected to overexpress an ALK2-HiBiT fusion. The cells were then treated with increasing concentrations of each compound for a 24 h period, and HiBiT levels were assessed using a HiBiT monoclonal antibody (Figure 2c). The immunoblot results highlighted that compound **11** induced substantial ALK2 degradation at concentrations >0.3 μM, whereas compound **12** did not substantially reduce ALK2 expression (<50% relative to control) at any of the tested concentrations.

**Table 1:** *In Vitro* Properties of First Generation ALK2 Degraders

Compound	NanoBRET ALK2 pIC_50_ ^a^	%ALK2 Remaining^b^	ChromLogD_7.4_/EPSA	HLM/MLM *T*_1/2_ (min)^c^	Kinetic Solubility (μM)^d^
**11**	6.6	46% at 1 μM	2.7/102	<5/<5	195
**12**	6.3	61% at 1 μM	2.4/101	<5/<5	ND

a ALK2 inhibition measured using a NanoBRET assay in HEK-293 cells (*n* = 3 biologically independent replicates).

b Relative ALK2 levels determined by immunoblotting following 24 h treatment with compound at a concentration of 1 μM; reported values are the mean of *n* = 2 biologically independent replicates.

c Metabolic stability determined in human and mouse liver microsomes (*n* = 2).

d Solubility assessed in fed state simulated intestinal fluid (FESSIF) at pH 5.0 (*n* = 2). ND = Not determined.

To investigate how these bifunctional compounds may behave under physiological conditions, the lipophilicity and effective polarity (EPSA)^36^ of each compound were measured using chromatographic methods and *in vitro* ADME data was generated (Table 1). Compounds **11** and **12** each demonstrated chameleonic behavior, with substantially lower EPSA values than their calculated TPSA values (158 and 186, respectively). This is likely to be associated with the flexibility of the PEG linker. Compound **11** possessed good solubility in fed-state simulated intestinal fluid (FESSIF), however, it suffered from metabolic instability in human and mouse liver microsomes. Due to the more promising ALK2 degradation profile, we focused our subsequent optimization efforts on developing CRBN-recruiting degraders with improved ALK2 degradation potency and metabolic stability.

### Second Generation ALK2 Degraders

We believed that incorporating rigidifying groups within the linker could yield potency improvements, by reducing the entropic penalty associated with ternary complex formation. Furthermore, we hypothesized that the major sites of metabolism were the methylenes of the PEG linker and, therefore, exploring alternatives could lead to improved metabolic stability. Based on these hypotheses, a second series of ALK2 degraders was prepared incorporating *N*-heterocycles within the linker portion (Figure 3). These compounds were screened for their ability to degrade ALK2 at a concentration of 1 μM, using HEK-293 cells that had been transfected to express an ALK2-HiBiT fusion (Figure S3 and Table 2). Furthermore, the metabolic stability of all compounds was assessed in liver microsomes, and kinetic solubility was measured in FESSIF (Table 2).

**Figure 3:**
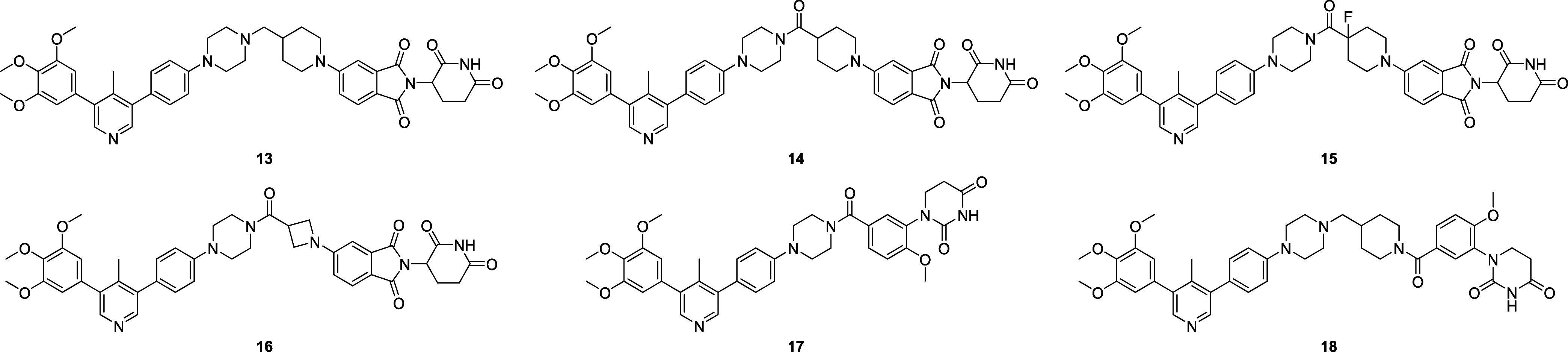
Structures of second generation ALK2 degraders.

**Table 2:** *In Vitro* Properties of Second Generation ALK2 Degraders Compared with Compound **11** as Reference

Compound	%ALK2 Remaining^a^	HLM/MLM *T*_1/2_ (min)^b^ [Table-fn t2fn5]	Kinetic Solubility (μM)^c^	clogD/TPSA/#RotB^d^
**11**	46	<5/<5	195	2.9/158/17
**13**	27	>73*/>100	17	3.2/134/10
**14**	53	34/50	54	3.0/151/9
**15**	40	41/>77*	8	3.0/151/9
**16**	86	30/24	9	2.6/151/9
**17**	67	>94*/40	>200	3.1/123/9
**18**	63	69/47	>200	3.3/126/11

a Relative ALK2 levels determined by immunoblotting following 24 h treatment with compound at a concentration of 1 μM; reported values are the mean of *n* = 2 biologically independent replicates.

b Metabolic stability determined in human and mouse liver microsomes (*n* = 2).

c Solubility assessed in fed state simulated intestinal fluid (FESSIF) at pH 5.0 (*n* = 2).

d Values calculated using Percepta Portal.

* Indicates that *T*_1/2_ was measured as this value in one replicate, and >100 min in a second replicate.

By replacing the PEG linker in **11** with a range of *N*-heterocycles, compound **13** was identified as inducing substantially more ALK2 degradation at a concentration of 1 μM. Furthermore, replacement of the PEG linker with *N*-heterocyclic groups led to substantial improvements in metabolic stability relative to **11** for all compounds. Interestingly, while compound **13** was less polar than compound **11** based on calculated metrics (TPSA), it possessed a higher EPSA value of 111, thus demonstrating reduced capacity for chameleonic behavior, consistent with the fact that it contained fewer rotatable bonds. Based on its promising degradation of ALK2, excellent stability in liver microsomes, and acceptable solubility, compound **13** was selected for further *in vitro* analysis and will be referred to as **M4K3233** (**13**) hereafter.

Dose–response immunoblotting experiments were conducted using **M4K3233** (**13**) in HEK-293 cells that were transfected to express an ALK2-HiBiT fusion. These experiments revealed that **M4K3233** (**13**) potently induced ALK2 degradation in a dose-dependent manner up to a concentration of approximately 1 μM, above which degradation plateaued at a value of approximately 60–65% (Figure 4a and 4b). The kinetics of ALK2 degradation were assessed by a time-course experiment conducted in HEK-293 cells treated with **M4K3233** (**13**) at a concentration of 0.1 μM and the cells lysed at various time points. ALK2-HiBiT expression was quantified by immunoblotting, revealing that ALK2 degradation was a time-dependent process (Figure 4c and 4d). Finally, intracellular ALK2 engagement by degrader **13** was confirmed in HEK-293 cells using a NanoBRET assay (Figure 4e).

**Figure 4:**
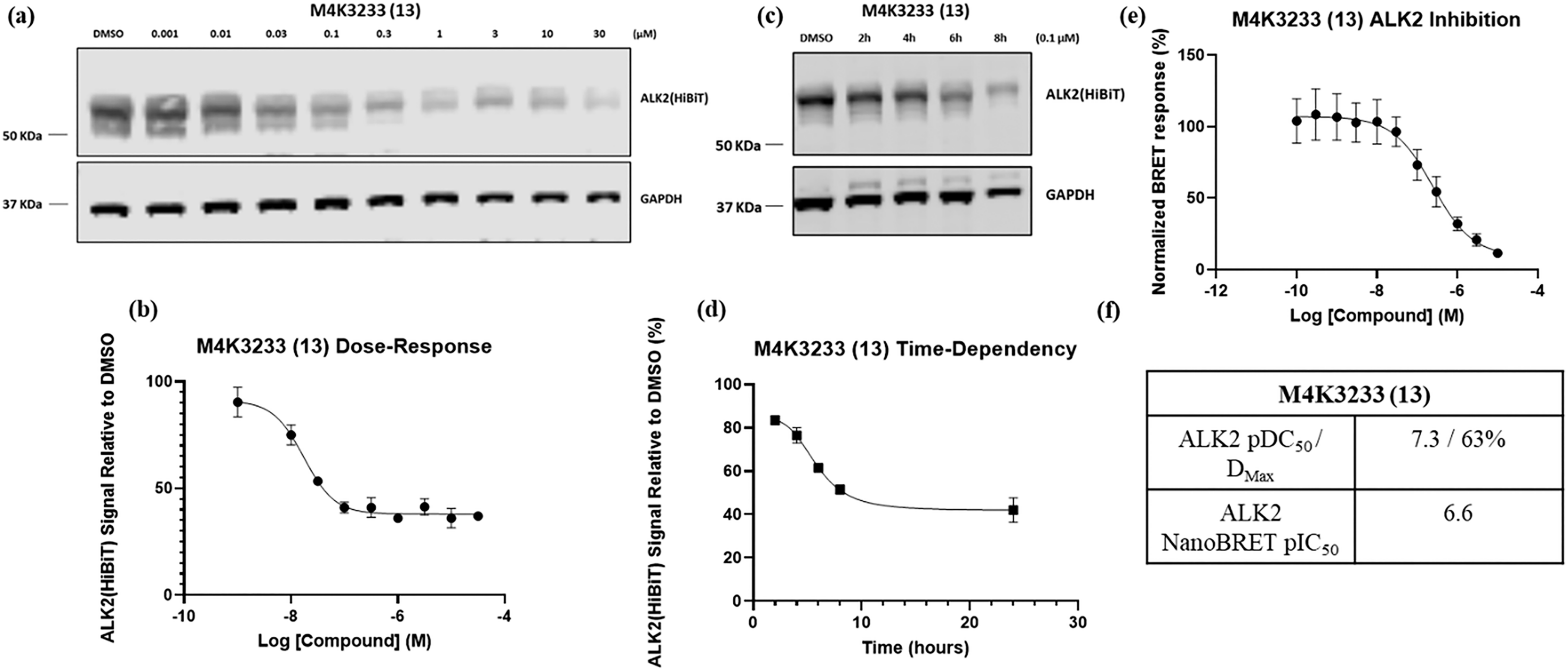
*In vitro* assessment of **M4K3233** (**13**). (a) ALK2-HiBiT transfected HEK-293 cells were treated for 24 h with increasing concentrations of **M4K3233** (**13**), then the cell lysates were analyzed by immunoblotting. (b) Quantification of Figure 4a. Data plotted is the mean ± standard error of mean (SEM) of *n* = 3 biologically independent replicates. (c) ALK2-HiBiT transfected HEK-293 cells were treated for the indicated time periods with 0.1 μM **M4K3233** (**13**), then the cell lysates were analyzed by immunoblotting. (d) Quantification of Figure 4c. Data plotted is the mean ± SEM of *n* = 2 biologically independent replicates. (e) ALK2 IC_50_ curve for **M4K3233** (**13**) determined using a NanoBRET assay in HEK-293 cells. Data plotted is the mean ± SEM of *n* = 3 biologically independent replicates. (f) Estimated ALK2 pDC_50_, *D*_max_, and pIC_50_ values for **M4K3233** (**13**) in HEK-293 cells generated using the [inhibitor] versus response (four-parameter) nonlinear regression curve fitting software of GraphPad Prism 9.

### Selectivity Profiling

To assess the selectivity of degradation, quantitative proteomics studies were conducted using ALK2-overexpressing HEK-293 cells, as well as U87-MG cells, which were expected to have high endogenous ALK2 expression. In addition to **M4K3233** (**13**), the glutarimide *N*-methylated analogue, **M4K3233NC** (**19**), was also synthesized and tested as a negative control compound lacking the ability to recruit CRBN. In HEK-293 cells, treatment with **M4K3233** (**13**) (1 μM, 24 h) induced a significant reduction in ALK2 levels (Log_2_FC = −1.5, *P.*value <0.0001), thus corroborating the immunoblotting results (Figure 5a). Two additional proteins were shown to be downregulated to a greater extent than ALK2 after compound treatment. More specifically, Phosphodiesterase 6 subunit delta (PDE6D) and FLT3-interacting zinc finger 1 (FIZ1) are both proteins that have recently been shown to be neo-substrates that are degraded by derivatives of immunomodulatory imide drugs (IMiDs).^37,38^ As such, it is plausible that the glutarimide region of **M4K3233** (**13**) binds to CRBN, modifying the protein surface and causing it to interact with PDE6D and FIZ1, resulting in their subsequent ubiquitination and degradation. Using **M4K3233NC** (**19**), it was demonstrated that degradation of each of these proteins relied on the binding of **M4K3233** (**13**) to CRBN (Figure 5b). In U87-MG cells, we were able to quantify ALK2 levels in all the DMSO-treated cell lysates but were only able to quantify ALK2 levels in 2 out of 5 lysates after treatment with **M4K3233** (**13**). In these lysates, a significant reduction in ALK2 levels was observed (Log_2_FC = −2.2); however, we excluded this data from our volcano plot (Figure 5c), as it did not meet our internal criteria for conducting statistical analysis. We hypothesized that in the lysates where ALK2 could not be detected, **M4K3233** (**13**) reduced ALK2 levels to below the level of quantification.

**Figure 5:**
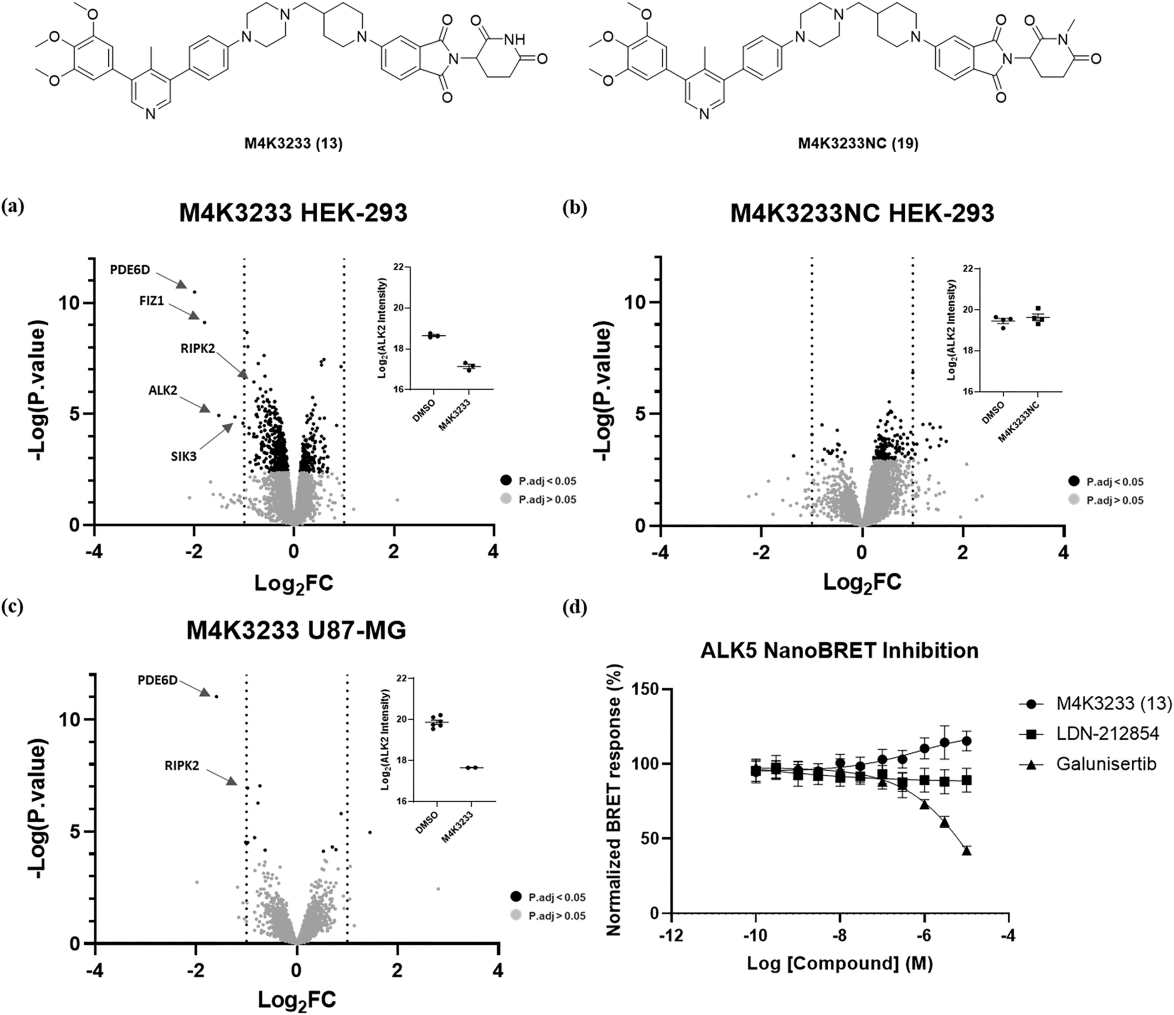
Selectivity profiling using **M4K3233** (**13**) and **M4K3233NC** (**19**). (a) Quantitative proteomics profiling in ALK2-overexpressing HEK-293 cells treated for 24 h with 1 μM **M4K3233** (**13**) vs DMSO (protein FDR < 5%, *n* = 3 biologically independent replicates per treatment). Log_2_(fold-change) difference between means of treated vs DMSO plotted against −log_10_(*P*.value). Lines in the plot indicate absolute [Log_2_(fold-change)] > 1. (b) Quantitative proteomics profiling in ALK2-overexpressing HEK-293 cells treated for 24 h with 1 μM **M4K3233NC** (**19**) vs DMSO (protein FDR < 5%, *n* = 4 biologically independent replicates per treatment). Log_2_(fold-change) difference between means of treated vs DMSO plotted against −log_10_(*P*.value). Lines in the plot indicate absolute [Log_2_(fold-change)] > 1. (c) Quantitative proteomics profiling in U87-MG cells treated for 24 h with 1 μM **M4K3233** (**13**) vs DMSO (protein FDR < 5%, *n* = 5–6 biologically independent replicates per treatment). Log_2_(fold-change) difference between means of treated vs DMSO plotted against −log_10_(*P*.value). Lines in the plot indicate absolute [Log_2_(fold-change)] > 1. (d) ALK5 IC_50_ curves for **M4K3233** (**13**), **LDN-212854** (**4**), and galunisertib, determined using a NanoBRET assay in HEK-293 cells. Data is plotted as the mean ± SEM of *n* = 3 biologically independent replicates.

From these experiments we were pleased to see that **M4K3233** (**13**) was a very selective kinase degrader, with salt-inducible kinase 3 (SIK3) being the only other kinase that was significantly down-regulated in HEK-293 cells (Log_2_FC = −1.2, *P*.value <0.0001), although in U87-MG cells, the expression of this protein was not significantly altered (Log_2_FC = −0.4, *P*.value = 0.05). Across both cell lines, receptor-interacting serine/threonine-protein kinase 2 (RIPK2) was also moderately down-regulated (HEK-293 Log_2_FC = −0.81, *P*.value <0.0001, U87-MG Log_2_FC = −0.98, *P*.value <0.0001). Additionally, some kinases which are reported to be inhibited by the parent ALK2 inhibitor, **M4K2009** (**6**), such as ABL1, TNIK, and MINK,^22^ were detected by mass-spectrometry and were found not to be differentially expressed after treatment with the degrader **M4K3233** (**13**). It should be noted that there may be additional kinases that can be degraded by **M4K3233** (**13**) but were not expressed sufficiently in the tested cell lines. It was also observed that **M4K3233** (**13**) displayed some selectivity with respect to CRBN neo-substrate degradation and did not alter the expression of reported IMiD neo-substrates such as GSPT1.^39^

A critical requirement of chemical probe compounds is selectivity for the desired target protein over related family proteins. Activin receptor-like kinase 5 (TGFBR1/ALK5) is a type-1 TGF-β pathway receptor, inhibition of which has been associated with cardiotoxicity and physeal dysplasia.^40–42^ Using a NanoBRET assay, we assessed the cellular engagement of ALK5 by **M4K3233** (**13**), including reported ALK2 and ALK5 inhibitors in these assays as positive and negative controls (Figure 5d and S2). In these studies, no ALK5 inhibition was observed up to a concentration of 30 μM by **M4K3233** (**13**) or the selective ALK2 inhibitor **LDN-212854** (**4**), although the reported ALK5 inhibitor galunisertib did inhibit ALK5 activity as expected. Furthermore, in our U87-MG proteomic studies, the expression of ALK5 was successfully quantified and was found not to be affected by **M4K3233** (**13**) treatment (Log_2_FC = 0.15, *P*.value = 0.38). Using proteomics, we were unable to investigate the effects of **M4K3233** (**13**) on other closely related BMP type-1 receptors such as ALK1, ALK3, and ALK6 due to their expression levels in the tested cell lines. Future work to progress the field of ALK2 degrader chemical probes should evaluate the development and application of nonproteomic approaches to measuring selectivity against other BMP type-1 receptors, as well as disease relevant ALK2 mutants such as ALK2-R206H.

### Mechanistic Studies

Using HEK-293 cells that had been transfected to express HiBiT-tagged ALK2, we investigated the mechanism of ALK2 degradation by **M4K3233** (**13**). First, we sought to demonstrate that recruitment of both ALK2 and CRBN was essential for ALK2 degradation by conducting competition experiments with the ALK2 ligand **M4K2009** (**6**), as well as the CRBN ligand, pomalidomide. These studies showed that **M4K3233** (**13**) was required to bind to both ALK2 and CRBN in order to induce ALK2 degradation (Figure 6a). We also showed that **M4K3233NC** (**19**) does not induce ALK2 degradation, consistent with our proteomics results (Figure 6a).^43^ For completeness, we also confirmed that **M4K3233NC** (**19**) was indeed capable of engaging ALK2 in HEK-293 cells using a NanoBRET assay (pIC_50_ = 5.9) (Figure S1).

**Figure 6:**
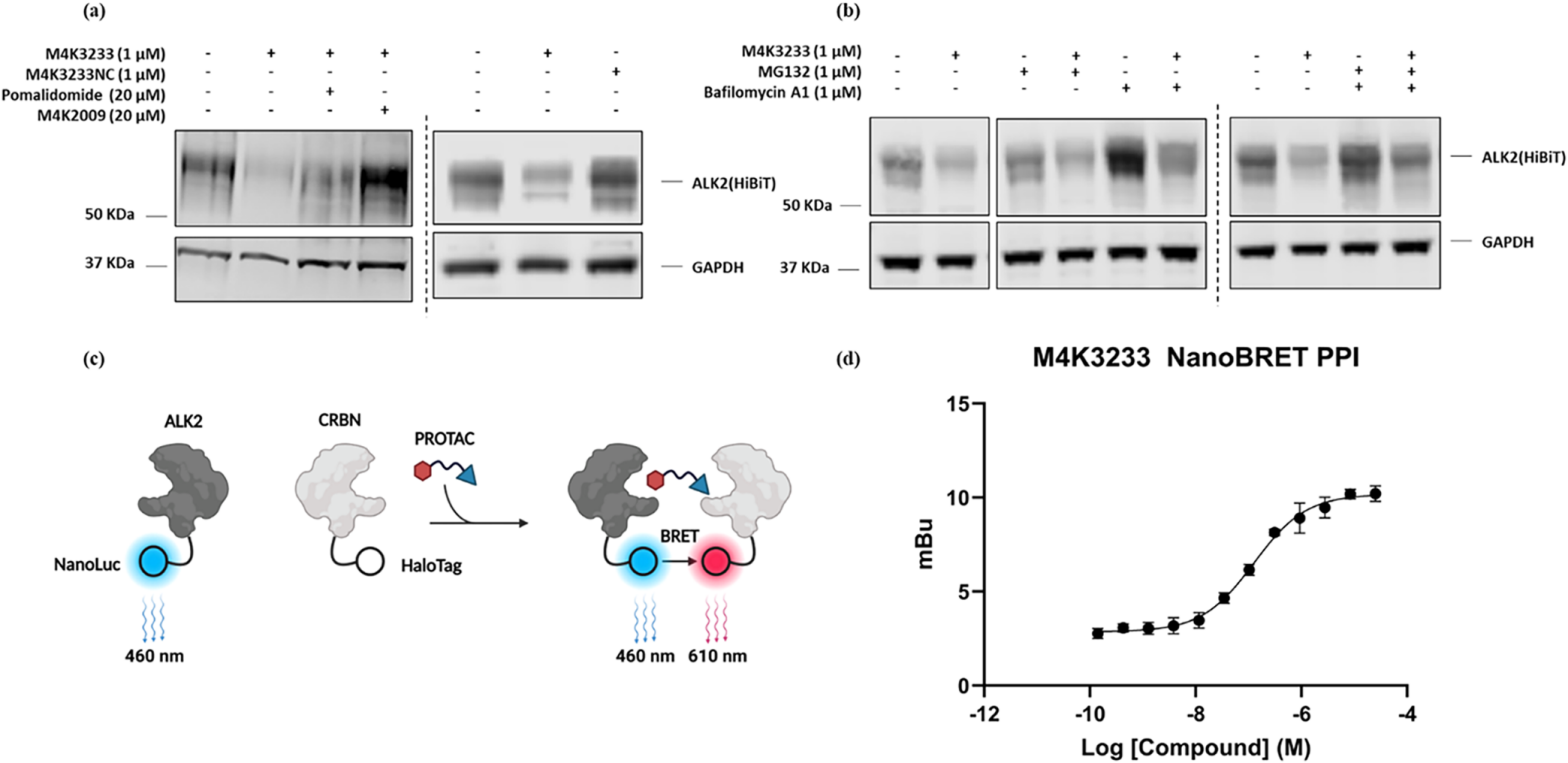
Mechanistic basis for ALK2 degradation. (a) Competition experiments in ALK2-HiBiT transfected HEK-293 cells using **M4K3233** (**13**), **M4K3233NC** (**19**), and reported ligands for ALK2 and CRBN. The cells were pretreated with pomalidomide or **M4K2009** (**6**) for 20 min prior to treatment with **M4K3233** (**13**) for 24 h. (b) Competition experiments in ALK2-HiBiT transfected HEK-293 cells using **M4K3233** (**13**) and reported inhibitors of the proteasome and lysosome. The cells were pretreated with MG132 and/or bafilomycin A1 for 20 min prior to treatment with **M4K3233** (**13**) for 24 h. (c) Schematic overview of NanoBRET protein–protein interaction (PPI) assay. (d) ALK2-CRBN NanoBRET PPI dose–response curve for **M4K3233** (**13**), determined in HEK-293 cells coexpressing ALK2-NanoLuc and HaloTag-CRBN. Data are plotted as mean ± SEM of technical triplicates from a representative biological experiment; pEC_50_ value reported in the text represents the mean ± SEM from four independent biological experiments.

Next, the role of the proteasome and lysosomal pathways on ALK2 degradation were assessed by conducting competition experiments with the proteasome inhibitor MG132, as well as bafilomycin A1, which inhibits lysosomal acidification. Interestingly, in each case, ALK2 levels were only partially recovered, suggesting that ALK2 degradation was partially reliant on both proteasomal and lysosomal pathways (Figure 6b). This hypothesis was supported by treating HEK-293 cells with both MG132 and bafilomycin A1, in addition to **M4K3233** (**13**), and under these conditions no ALK2 degradation was observed (Figure 6b). These combined experiments suggested that ALK2 degradation by **M4K3233** (**13**) in HEK-293 cells occurred via a CRBN-mediated, ubiquitin, proteasome/lysosome combinatorial pathway. This unusual mechanism of degradation is consistent with that reported for the epidermal growth factor receptor (EGFR), which is a transmembrane kinase like ALK2.^29,44^ Similarly, it was recently demonstrated that a CRBN-recruiting PROTAC induced the degradation of the GPCR CC chemokine receptor 2 (CCR2) via the lysosomal pathway.^45^

To further investigate the mechanism of ALK2 degradation, a NanoBRET protein–protein interaction (PPI) assay was developed to confirm that **M4K3233** (**13**) induces proximity between ALK2-Nanoluc and CRBN-Halotag in live HEK-293 cells (Figure 6c). **M4K3233** (**13**) produced a dose-dependent increase in NanoBRET signal, with an apparent potency of pEC_50_ = 7.42 ± 0.22 (mean ± SEM, *n* = 4 independent biological experiments), demonstrating that **M4K3233** (**13**) promotes intracellular ALK2-CRBN proximity consistent with PROTAC-mediated ternary complex formation (Figure 6d).

### Third Generation ALK2 Degraders

We were interested in the development of ALK2 degraders as a potential therapeutic strategy for glioblastoma and DIPG, both of which are diseases affecting the central nervous system. As such, we next explored strategies which had the potential to improve the likelihood of compounds being able to penetrate the blood-brain barrier. Smil et al. showed previously that replacement of the trimethoxyphenyl ring in the ALK2 inhibitor **M4K2009** (**6**) with a 4-fluoro-3,5-dimethoxyphenyl group as in **M4K2163** (**7**) resulted in a significant increase in CNS penetration *in vivo*.^22^ Based on this, we prepared a small number of CRBN-recruiting ALK2 degraders containing this structural motif and assessed the ability of these compounds to degrade ALK2 at a concentration of 1 μM, as well as their *in vitro* ADME properties (Figure 7, Table 3, and S3). It should be noted that pyrrolidine-containing compounds **22** and **23** were isolated and subject to biological evaluation as a mixture of diastereomers.

**Figure 7:**
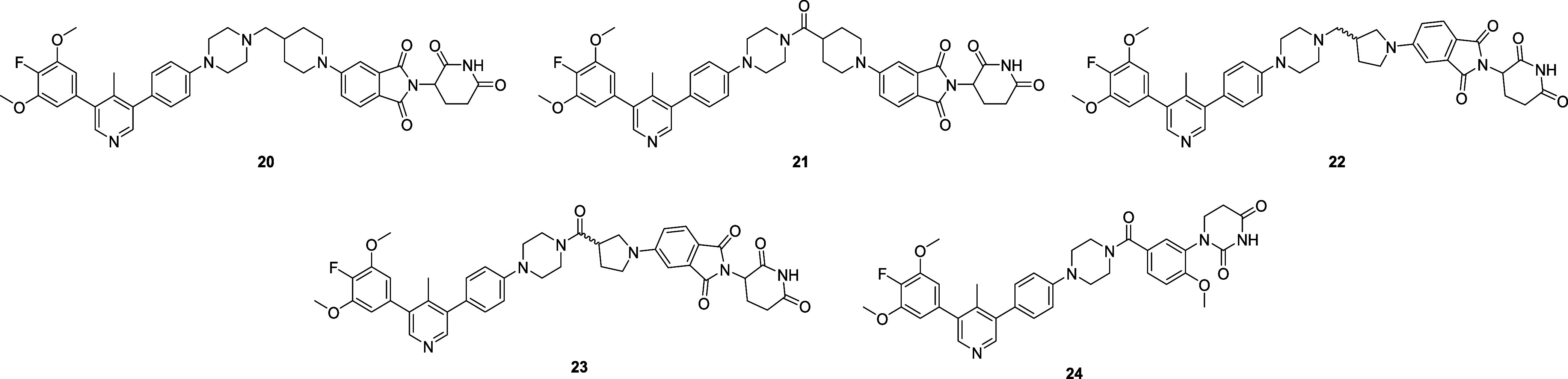
Chemical structures of third generation ALK2 degraders.

**Table 3:** *In Vitro* Properties of Third Generation ALK2 Degraders Compared with Compound **13** as Reference

Compound	% ALK2 Remaining^a^	HLM/MLM *T*_1/2_ (min)^b^ [Table-fn t3fn5]	Kinetic Solubility (μM)^c^	clogD/TPSA/#RotB^d^	ChromLogD_7.4_/EPSA
**M4K** **3233 (13)**	27	>73*/>100	17	3.2/134/10	3.6/111
**20**	4	>95*/66	<5	4.1/125/9	3.5/108
**21**	29	>96*/>88*	<5	3.2/142/8	ND/ND
**22**	19	>100/>84*	<5	3.8/125/9	3.4/111
**23**	52	66/41	<5	3.3/142/8	3.0/114
**24**	42	68/47	<5	3.7/114/8	ND/ND

a Relative ALK2 expression determined by immunoblotting following 24 h treatment with each compound at a concentration of 1 μM; reported values are the mean of *n* = 2 biologically independent replicates.

b Metabolic stability determined in human and mouse liver microsomes (*n* = 2).

c Solubility assessed in fed state simulated intestinal fluid (FESSIF) at pH 5.0 (*n* = 2).

d Values calculated using Percepta Portal.

* Indicates that *T*_1/2_ was measured as this value in one replicate, and >100 min in a second replicate.

Incorporation of a fluorine atom within the ALK2 binding portion had a beneficial effect on ALK2 degradation ability, with all compounds inducing approximately 50% or more ALK2 degradation at a concentration of 1 μM. Furthermore, the compounds in this iteration possessed excellent metabolic stability in human liver microsomes, (*T*_1/2_ > 60 min for all compounds), although incorporation of the fluorine atom was accompanied by a reduction in kinetic solubility, which may have had an impact on the observed stability in the microsomal stability assay. Compound **20**, referred to as **M4K3250** (**20**) hereafter, is the matched-molecular pair of **M4K3233** (**13**) and was shown to induce a substantial depletion in ALK2-HiBiT levels at a concentration of 1 μM. Follow-up immunoblotting experiments confirmed **M4K3250** (**20**) to be a very promising ALK2 degrader (pDC_50_ = 7.9, *D*_Max_ = 78%) (Figure 8a and 8b). Using NanoBRET assays, we confirmed that **M4K3250** (**20**) inhibited ALK2, but not ALK5 in cells, and demonstrated, by NanoBRET PPI, that **M4K3250** (**20**) induces ternary complex formation between ALK2 and CRBN with an apparent potency of pEC_50_ = 6.9 ± 0.27 (mean ± SEM, *n* = 3 independent biological experiments) (Figure 8c and 8d).

**Figure 8:**
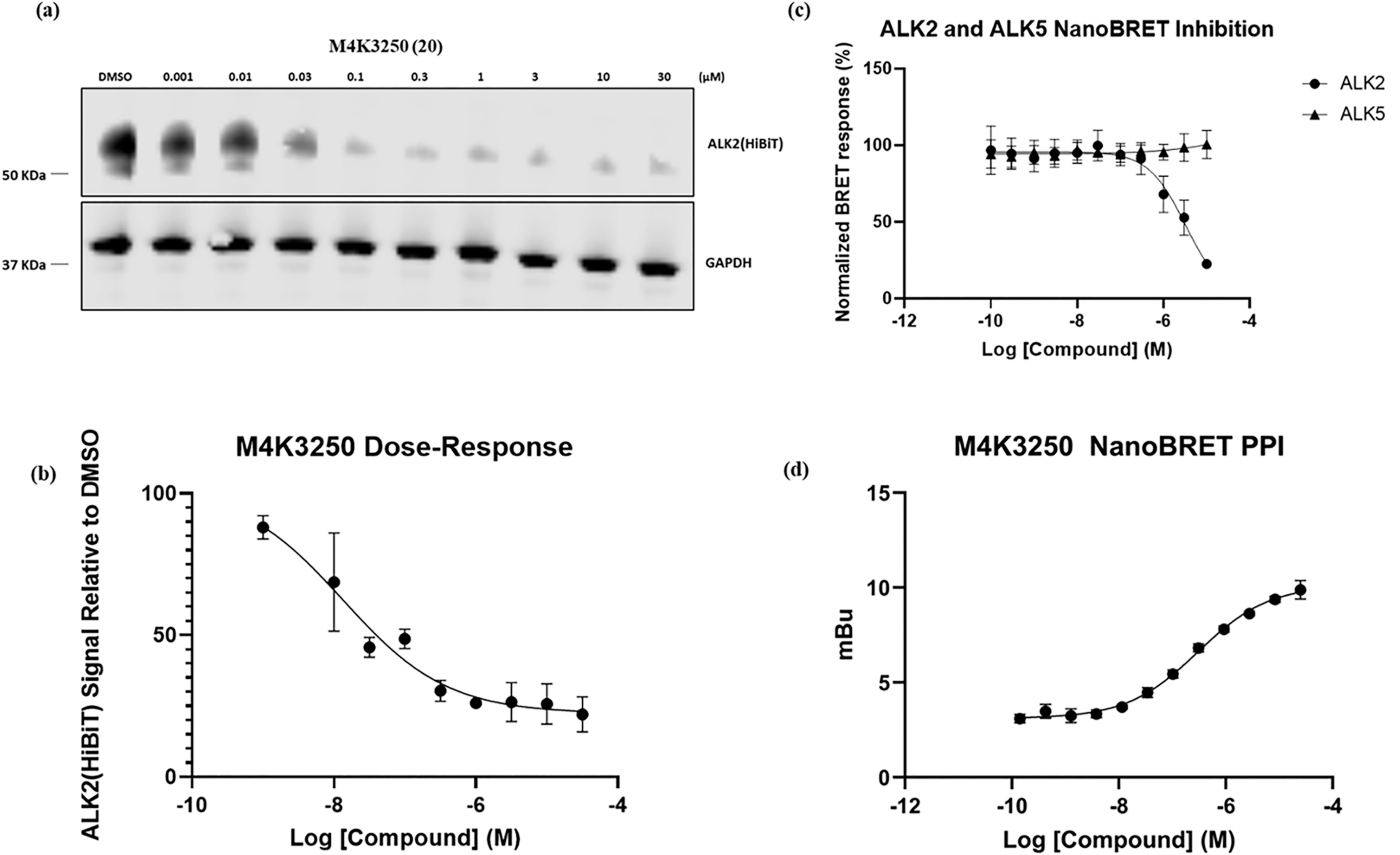
*In vitro* assessment of **M4K3250** (**20**). (a) ALK2-HiBiT transfected HEK-293 cells were treated for 24 h with increasing concentrations of **M4K3250** (**20**), then the cell lysates were analyzed by immunoblotting. (b) Quantification of Figure 8a. Data plotted is the mean ± standard error of mean (SEM) of *n* = 3 biologically independent replicates. (c) ALK2 and ALK5 IC_50_ curves for **M4K3250** (**20**) determined using a NanoBRET assay in HEK-293 cells. Data plotted is the mean ± SEM of *n* = 3 biologically independent replicates. (d) ALK2-CRBN NanoBRET PPI dose–response curve for **M4K3250 (20)** in HEK-293 cells coexpressing ALK2-NanoLuc and HaloTag-CRBN. Data are shown as the mean ± SEM of technical triplicates from a representative biological experiment; the pEC_50_ value reported in the text represents the mean ± SEM from three independent biological experiments.

### Phenotypic Studies

To assess the therapeutic potential of ALK2 degradation, concentration–response assays were performed across a panel of patient-derived pediatric-diffuse high-grade glioma (PDHGG) models to measure the ability of the ALK2 degrader **M4K3233** (**13**) to inhibit cell proliferation. The non-CRBN recruiting negative control analogue **M4K3233NC** (**19**) was also evaluated in these assays to deconvolute between the phenotypic effects of ALK2 inhibition and ALK2 degradation. These models represent the DMG-H3K27-altered and DHG-H3G34R/V subgroups of PDHGG and exhibit different mutational backgrounds as summarized in Figure S4. The cells were treated with increasing concentrations of each compound for 8 days, then cell viability was assessed by CellTiter-Glo (CTG) assay. The ALK2 degrader **M4K3233** (**13**) and the non-CRBN recruiting analogue **M4K3233NC** (**19**) showed similar potency, with GI_50_ determinations ranging from approximately 0.3 μM to 5 μM and 0.3 μM to 8 μM, respectively (Figure 9a), suggesting that the observed phenotypic effect was independent of CRBN-mediated ALK2 degradation. We next compared the potency of these compounds (**13** and **19**) to their parent ALK2 inhibitor **M4K2009** (**6**), which is reported to be a more potent ALK2 inhibitor than the bifunctional compounds (ALK2 NanoBRET pIC_50_ = 7.3).^22^ Intriguingly, **M4K2009** (**6**) showed lower potency than either of the bifunctional compounds but greater selectivity in the *ACVR1* mutant DMG-H3K27-altered models compared to *ACVR1*-wt DMG-H3K27-altered and DHG-H3G34R/V models (Figure 9a). As such, it was suggested that the phenotypic effects of **M4K3233** (**13**) and **M4K3233NC** (**19**) were unlikely to be caused by ALK2 inhibition alone.

**Figure 9:**
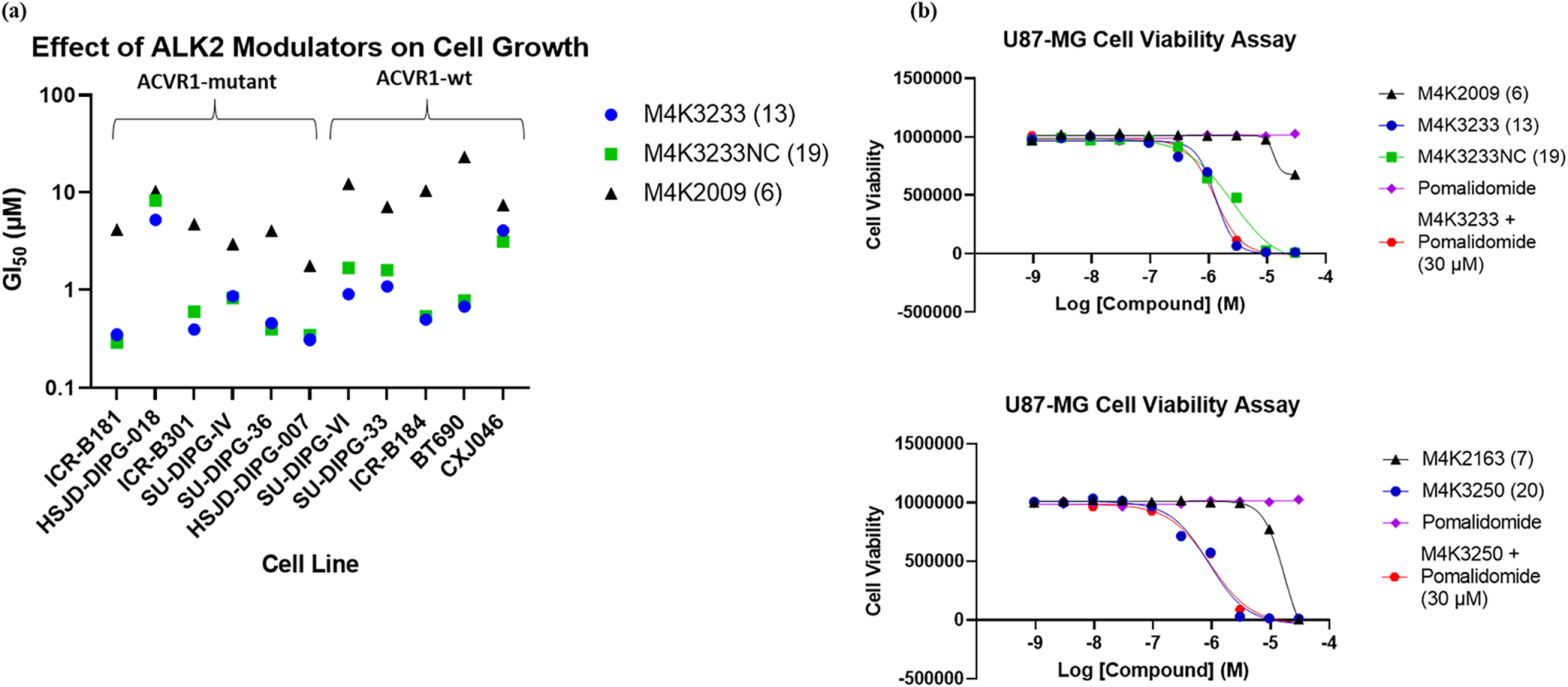
Phenotypic effects of ALK2 modulators in cancer cell lines. (a) GI_50_ determinations for **M4K3233** (**13**), **M4K3233NC** (**19**), and **M4K2009** (**6**) in a panel of patient-derived PDHGG models. Cells were treated with the indicated compounds at a range of concentrations for 8 days, then cell viability was assessed by CellTiter-Glo assay and GI_50_ values were determined from at least two biological replicates. (b) Cell viability assay results for **M4K3233** (**13**), **M4K3233NC** (**19**), **M4K2009** (**6**), **M4K3250** (**20**), and **M4K2163** (**7**) in U87-MG cells. Cells were treated with the indicated compounds at a range of concentrations, for a period of 4 days, then cell viability was assessed using a CTG assay. Data plotted is the mean ± SEM of *n* = 3 biologically independent replicates.

Next, we conducted further cell viability assays using U87-MG glioblastoma cells, which were reported to be sensitive to the ALK2 inhibitor **DMH-1** (**10**) by Langenfeld et al.^26^ In addition to the previously tested compounds, we also used the degrader **M4K3250** (**20**) and its parent ALK2 inhibitor **M4K2163** (**7**) in these experiments. As a separate set of controls, the ALK2 degraders were cotreated with pomalidomide (30 μM), which would serve to block CRBN recruitment. Consistent with the previous results, the ALK2 degraders were found to be more potent cytotoxic agents than the parent ALK2 inhibitors, however, blocking CRBN recruitment by these compounds did not affect their ability to induce cytotoxicity (Figure 9b). Based on these collective findings, we suggest that the phenotypic effects of **M4K3233** (**13**), **M4K3233NC** (**19**), and **M4K3250** (**20**) in glioma and glioblastoma cells arise from unknown polypharmacology.

### Chemistry

The ALK2 inhibitors **M4K2009** (**6**), and **M4K2163** (**7**), which served as the starting points for the bifunctional degraders, were prepared in three steps starting from 3,5-dibromo-4-methylpyridine (Scheme 1).

**Scheme 1:**
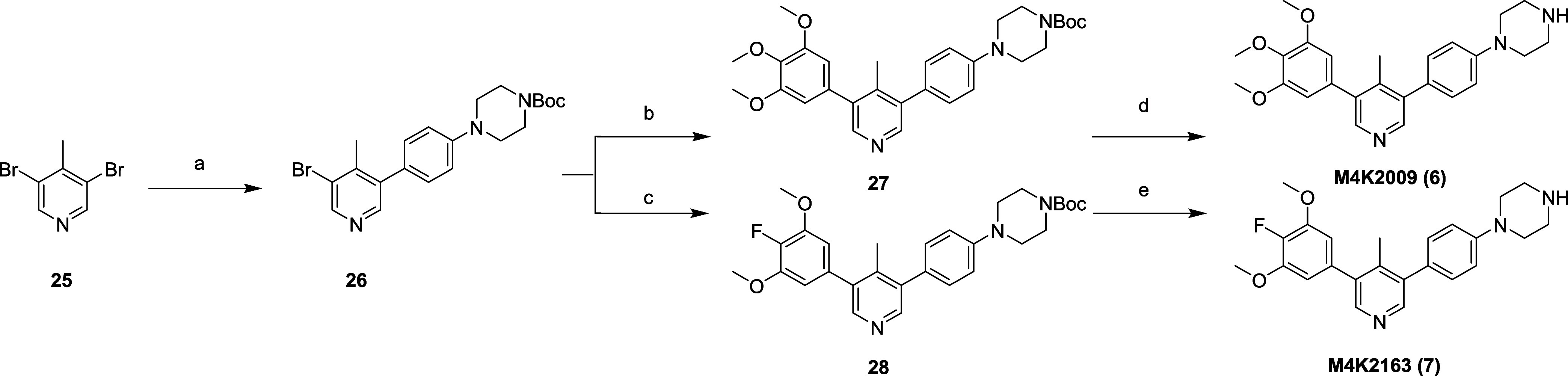
Synthesis of ALK2 Ligands **6** and **7** ^a^

a Reagents and conditions: (a) 4-(4-*tert*-butoxycarbonylpiperazinyl)­phenylboronic acid pinacol ester, K_3_PO_4_, Pd­(dppf)­Cl_2_ (10 mol %), 1,4-dioxane/water (4:1), 70 °C, 5 h, 39%; (b) 3,4,5-trimethoxyphenylboronic acid, K_3_PO_4_, Pd­(dppf)­Cl_2_ (9 mol %), CPME/water (4:1), 80 °C, 4 h, 57%; (c) (4-fluoro-3,5-dimethoxyphenyl)­boronic acid, K_3_PO_4_, Pd­(dppf)­Cl_2_ (9 mol %), 1,4-dioxane/water (4:1), 80 °C, 4 h, 79%; (d) 3 M HCl in CPME, DCM, r.t., 2 h, 92%; (e) 4 M HCl in 1,4-dioxane, DCM, r.t., 3 h, 96%.

To prepare compound **11**, triethylene glycol monobenzyl ether (**29**) was converted to the corresponding mesylate and then conjugated to 5-hydroxythalidomide. The product was hydrogenated to remove the benzyl protecting group, then the alcohol oxidized to the corresponding aldehyde, which was reacted with **M4K2009** (**6**) under reductive amination conditions to afford the final product **11** (Scheme 2). To prepare compound **12**, triethylene glycol monobenzyl ether (**29**) was reacted with *tert*-butyl bromoacetate, the substitution product was then debenzylated using standard hydrogenation conditions, and the resultant free alcohol mesylated to form intermediate **35**, which was used to alkylate **M4K2009** (**6**). The *tert*-butyl ester in **36** was then deprotected and the corresponding carboxylic acid converted to the acyl chloride, which enabled formation of the amide by reacting with the commercially available amine VHL ligand to afford compound **12** (Scheme 2).

**Scheme 2:**
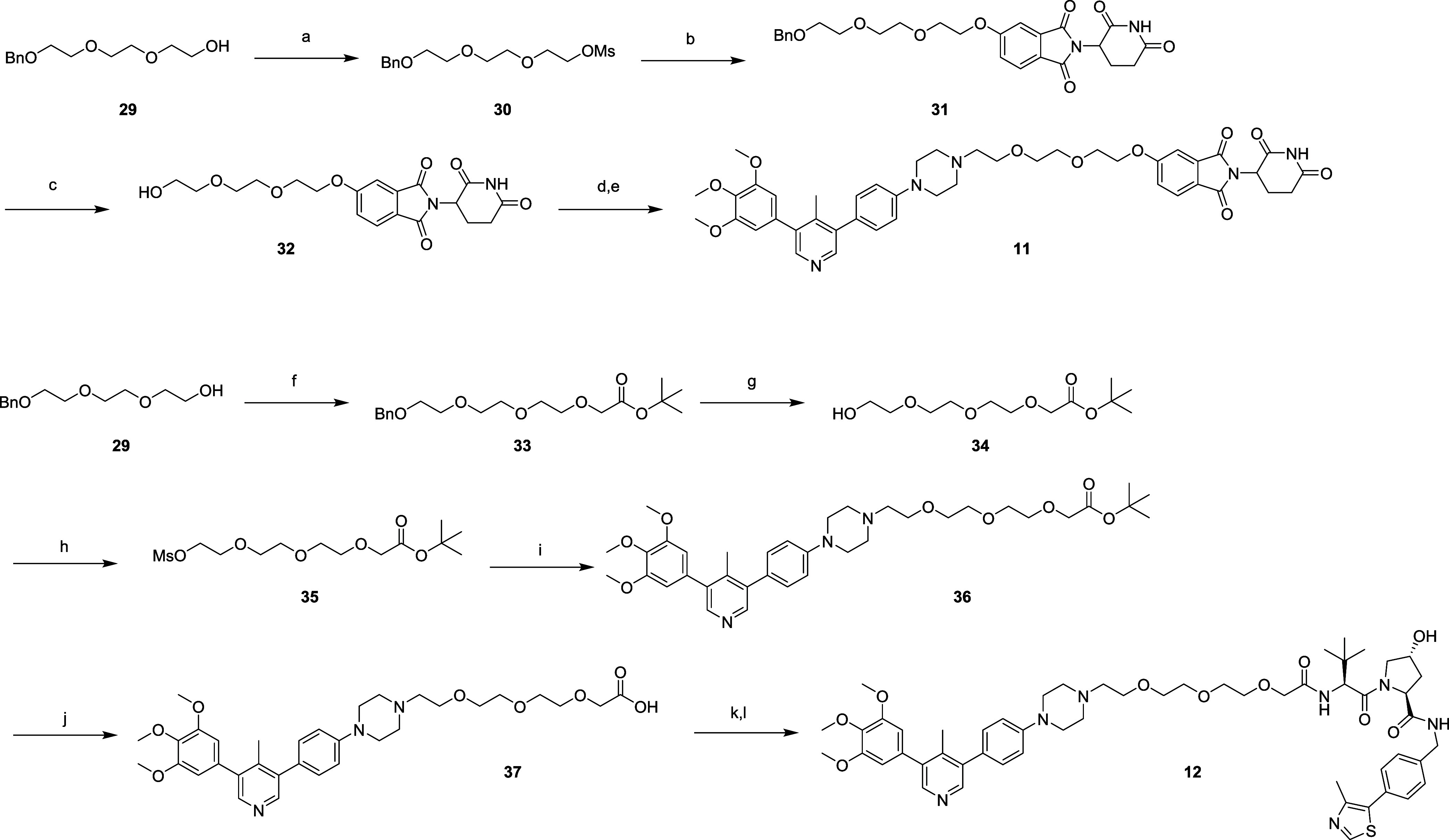
Synthesis of Compounds **11** and **12** ^a^

a Reagents and conditions: (a) Et_3_N, MsCl, DCM, 0 °C - r.t., 24 h, 78%; (b) 5-hydroxythalidomide, K_2_CO_3_, DMF, 80 °C, 24 h, 18%; (c) 1-methyl-1,4-cyclohexadiene, 10% Pd/C, EtOH, 80 °C, 17 h, 81%; (d) DMP, DCM, r.t., 3 h; (e) **M4K2009** (**6**), STAB, r.t., 17 h, 10% (over 2 steps); (f) *tert*-butyl bromoacetate, KO*t*Bu, THF, 0 °C - r.t., 72 h, 47%; (g) H-Cube, 10% Pd/C cartridge, MeOH, 60 °C, 1 atm, 2 h, 77%; (h) MsCl, Et_3_N, DCM, 0 °C - r.t., 17 h, quant; (i) **M4K2009** (**6**), K_2_CO_3_, MeCN, 80 °C, 72 h, 79%; (j) 4 M HCl in 1,4-dioxane, r.t., 1 h, quant; (k) SOCl_2_, DCM, 40 °C, 3 h; (l) VH032.HCl, Et_3_N, DMF, r.t., 17 h, 4% (over 2 steps).

To prepare the second and third iterations of ALK2 degraders, the ALK2 inhibitors **M4K2009** (**6**), and **M4K2163** (**7**) were conjugated to a selection of Boc-protected *N*-heterocycles using alkylation and amide coupling reactions. The intermediates formed were then Boc-deprotected under acidic conditions to afford intermediates **38**–**45** (Scheme 3).

**Scheme 3:**
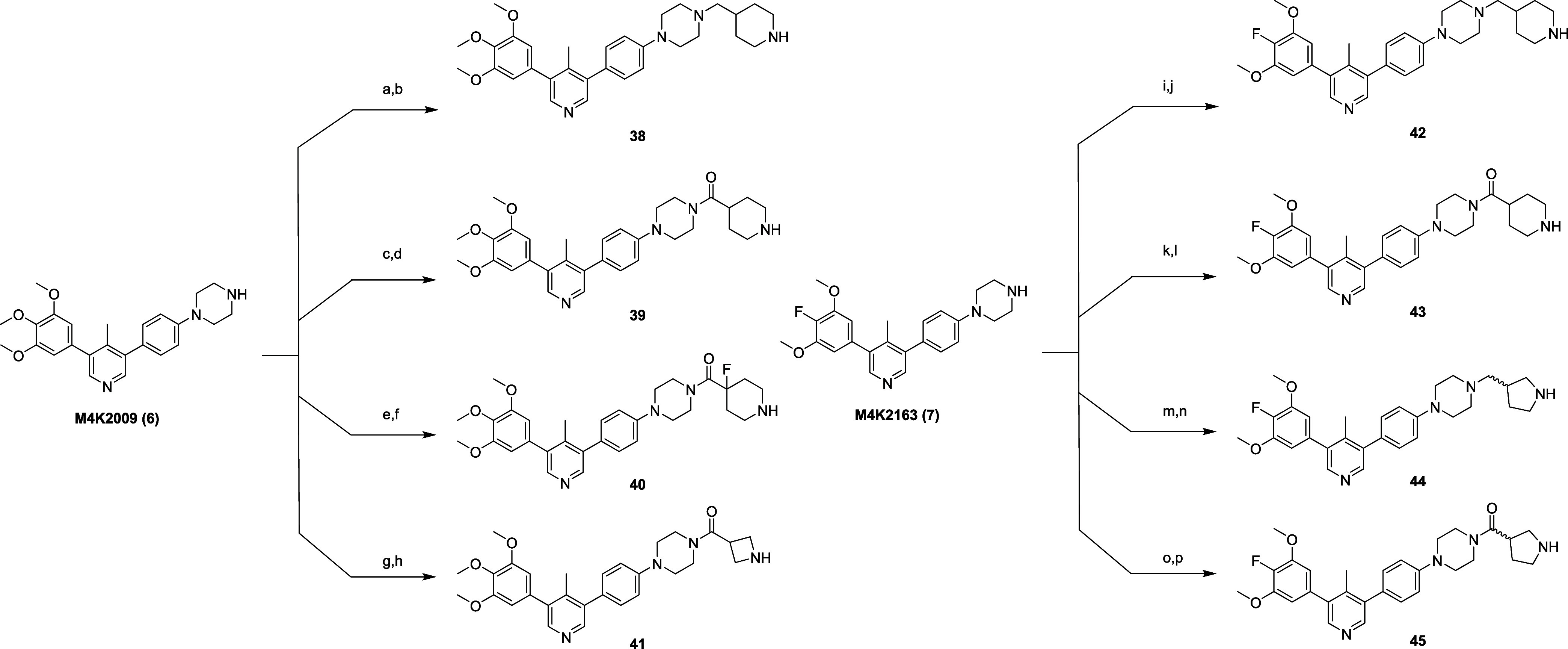
Synthesis of Intermediates **38**–**45** ^a^

a Reagents and conditions: (a) 1-Boc-4-bromomethylpiperidine, K_2_CO_3_, DMF, 120 °C, 17 h; (b) 4 M HCl in 1,4-dioxane, DCM, r.t., 2 h, 54% (over 2 steps); (c) 1-Boc-piperidine-4-carboxylic acid, T3P in EtOAc, Et_3_N, DMF, 50 °C, 3 h, (d) 4 M HCl in 1,4-dioxane, DCM, r.t., 17 h, 74% (over 2 steps); (e) 1-Boc*-*4-fluoropiperidine-4-carboxylic acid, T3P in EtOAc, Et_3_N, DMF, 50 °C, 3 h; (f) 4 M HCl in 1,4-dioxane, DCM, r.t., 17 h, 61% (over 2 steps); (g) 1-Boc-azetidine-3-carboxylic acid, T3P in EtOAc, Et_3_N, DMF, 50 °C, 3 h, (h) 4 M HCl in 1,4-dioxane, DCM, r.t., 17 h, 45% (over 2 steps); (i) 1-Boc-4-bromomethylpiperidine, K_2_CO_3_, DMF, 120 °C, 17 h; (j) 4 M HCl in 1,4-dioxane, DCM, r.t., 2 h, 46% (over 2 steps); (k) 1-Boc-piperidine-4-carboxylic acid, T3P in EtOAc, Et_3_N, DMF, 50 °C, 3 h, (l) 4 M HCl in 1,4-dioxane, DCM, r.t., 17 h, 79% (over 2 steps); (m) *tert*-butyl 3-(bromomethyl)­pyrrolidine-1-carboxylate, K_2_CO_3_, DMF, 120 °C, 17 h; (n) 3 M HCl in CPME, DCM, r.t., 2 h, 42% (over 2 steps); (o) 1-Boc-pyrrolidine-3-carboxylic acid, HATU, Et_3_N, DMF, 50 °C, 3 h, (p) 3 M HCl in CPME, DCM, r.t., 17 h, 54% (over 2 steps).

From these intermediates, compounds **13**–**16**, and **19–23** were prepared using S_N_Ar reactions, and compounds **17**, **18**, and **24** were prepared using amide coupling processes (Scheme 4).

**Scheme 4:**
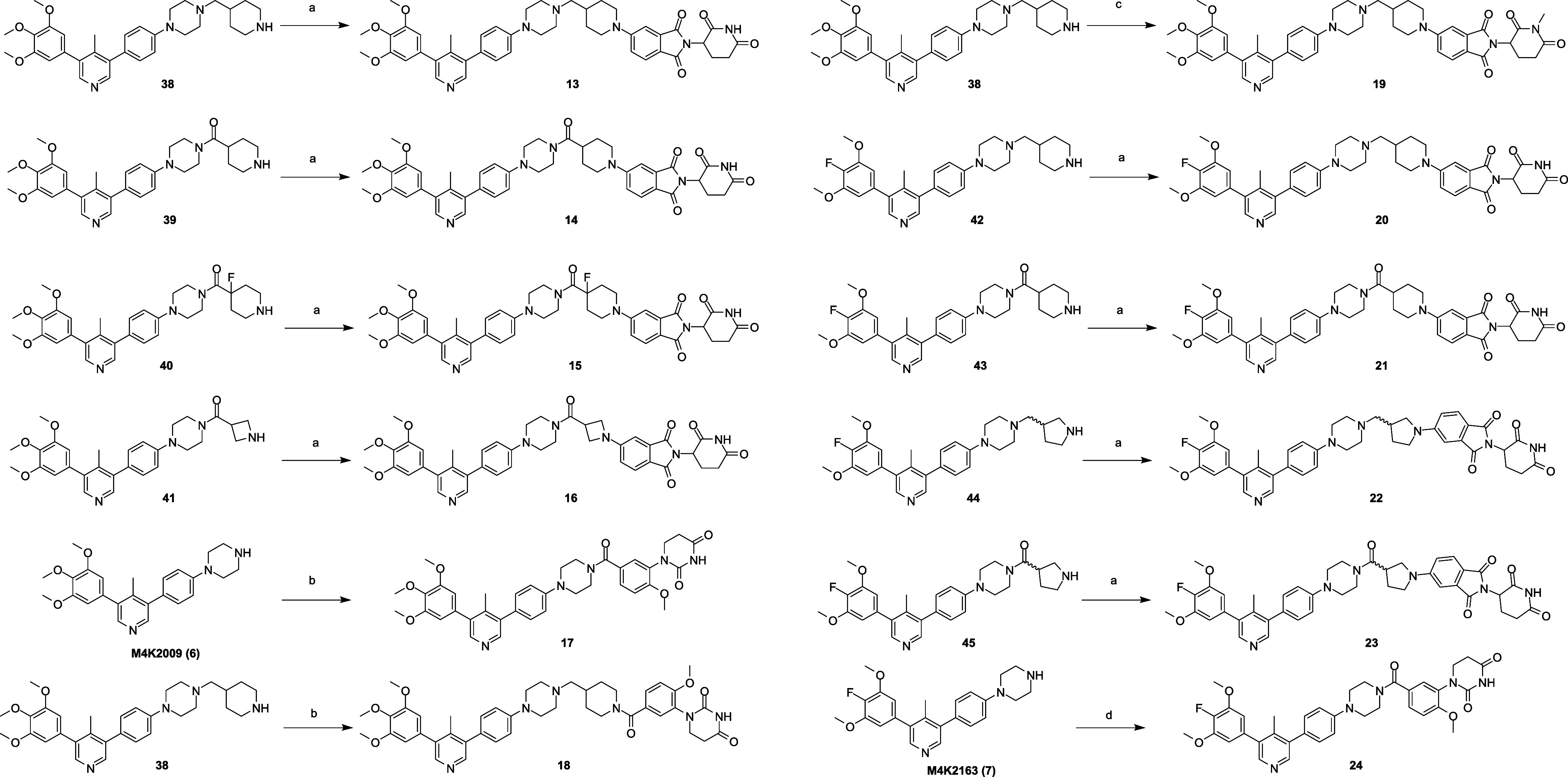
Synthesis of Final Compounds **13**–**24** ^a^

a Reagents and conditions: (a) 2-(2,6-dioxo-3-piperidyl)-5-fluoroisoindoline-1,3-dione, Et_3_N, DMF, 100 °C, 17 h, 9–21%; (b) 3-(2,4-dioxohexahydropyrimidin-1-yl)-4-methoxybenzoic acid, T3P in EtOAc, DIPEA, THF, 50 °C, 17 h, 8–44%; (c) 5-fluoro-2-(1-methyl-2,6-dioxo-3-piperidyl)­isoindoline-1,3-dione, Et_3_N, DMF, 100 °C, 17 h, 16%; (d) 3-(2,4-dioxohexahydropyrimidin-1-yl)-4-methoxybenzoic acid, T3P in EtOAc, DIPEA, DMF, 50 °C, 17 h, 21%.

## Conclusions

Activin receptor-like kinase 2 (ALK2) is a preclinically validated therapeutic target for the treatment of multiple conditions, including FOP disorder and DIPG, which currently lack effective treatment options. While ALK2 regulation has been extensively studied, most of the reported efforts to date have centered on using ATP-competitive small molecule inhibitors to achieve this. In this study we synthesized heterobifunctional compounds featuring an ALK2 ligand connected to ligands for the E3 ligases VHL and CRBN and are the first to demonstrate the degradation of ALK2 using such an approach. Subsequent optimization efforts resulted in the identification of potent ALK2 degraders with good metabolic stability in human and mouse liver microsomes, as well as acceptable kinetic solubility. Using one of the most promising degraders, **M4K3233** (**13**), we studied the mechanism of ALK2 degradation in HEK-293 cells and have discovered that ALK2 degradation is subject to both proteasomal and lysosomal pathways. To assess the utility of **M4K3233** (**13**) as a chemical probe compound, the selectivity of ALK2 degradation was assessed using mass spectrometry-based proteomics, demonstrating that **M4K3233** (**13**) is a highly selective ALK2 degrader. The phenotypic effects of the ALK2 degraders were assessed by conducting cell viability assays in cancer cell lines which have been reported to be sensitive to ALK2 inhibition. In these studies, the ALK2 degraders were shown to be more potent antiproliferative agents than the parent ALK2 inhibitors; however, we subsequently showed using a series of control experiments that these desirable results were not the result of direct effects on ALK2 and instead were occurring through an undefined mechanism. It is suggested that the cytotoxic effect of these compounds arises from polypharmacology and, as a consequence, the decision was taken to discontinue the development of these ALK2 degraders for applications in DIPG and glioblastoma. These results highlight the critical importance of using appropriate control experiments when conducting phenotypic assays, without which it is possible to incorrectly assume that the phenotypic effect observed derives from an on-target effect/targeted mechanism of action, as opposed to off-target or, indeed, polypharmacology effects. We believe that the compound classes disclosed herein have potential further importance as aligned with applications in studying the role of ALK2 in conditions such as FOP disorder and myelofibrosis.

## Experimental Section

### Chemistry

Chemical reagents that were commercially available were purchased from Apollo Scientific, Sigma-Aldrich, Fluorochem, Enamine, and PharmaBlock, and were used without further purification. All solvents used for reactions were anhydrous unless stated otherwise. LCMS analysis was conducted using one of two methods: (i) Acidic LC-MS was performed on a Waters 2795/2695 separations module with a Waters Diode Array Detector coupled to a single quadrupole mass spectrometer using an Agilent Zorbax column (4.6 × 50 mm, 3.5 μm, at 40 °C); a linear gradient of 5–95% MeCN (mobile phase), with 0.1% formic acid in water (0.1% formic acid) within 2 min was used (Flow rate = 3.9 mL/min); and (ii) Basic LC-MS was performed on a Waters 2795 separations module with a Waters Diode Array Detector coupled to a single quadrupole mass spectrometer using a Waters Sunfire C_18_ column (4.6 × 50 mm, 3.5 μm, at 40 °C); a linear gradient of 5–95% MeCN (mobile phase) in water, with 10 mM ammonium bicarbonate within 2.5 min was used (Flow rate = 2.0 mL/min). Purity of final compounds was assessed by UPLC-MS or HPLC, and all final compounds were >95% pure using these methods. UPLC-MS was carried out using UPLC + Waters DAD + Waters SQD2, single quadrupole UPLC-MS, and an Acquity UPLC HSS C_18_ 1.8 μm 100 × 2.1 mm column maintained at room temperature, running a linear gradient of 5–95% acetonitrile + 0.1% v/v formic acid in water + 0.1% v/v formic acid (0.4 mL/min over a period of 15 min). HPLC data were obtained on an Agilent 1200 series HPLC using a Machery-Nagel Nucleodur C_18_ column and a gradient method 5–95% acetonitrile (containing 0.1% TFA) in water (containing 0.1% TFA) over 18 min at a flow rate of 2 mL/min and UV monitoring at 250 nm. Infrared spectra were recorded on a Shimadzu IR Affinity-1 Spectrophotometer machine; wavenumbers are indicated in cm^–1^. High-resolution mass spectra were recorded using a Thermoscientific Exactive Plus equipped with a Vanquish LC. ^1^H Nuclear magnetic resonance (NMR) spectroscopy was carried out using a Bruker AVIII or AVNEO instrument operating at 400 or 500 MHz using the stated solvent at room temperature unless otherwise stated. Characteristic chemical shifts (δ) are given in parts-per-million using conventional abbreviations for designation of major peaks: e.g., s, singlet; d, doublet; t, triplet; q, quartet; dd, doublet of doublets; dt, doublet of triplets; m, multiplet; br, broad. Chemical shifts are quoted with respect to either an internal standard (SiMe_4_, 0.00 ppm), or with respect to residual nondeuterated solvent peak ((CD_3_)_2_SO, 2.50 ppm; CDCl_3_, 7.26 ppm; CD_3_CN 1.94 ppm; CD_3_OD 3.31 ppm). ^13^C Nuclear magnetic resonance (NMR) spectroscopy was carried out using a Bruker AVIII or AVNEO instrument operating at 101 or 126 MHz using the stated solvent at around room temperature unless otherwise stated. Characteristic chemical shifts (δ) are given in parts-per-million. Chemical shifts are quoted with respect to residual nondeuterated solvent peak ((CD_3_)_2_SO, 39.5 ppm; CDCl_3_, 77.2 ppm; CD_3_CN 1.3 ppm; CD_3_OD 49.0 ppm). ^19^F Nuclear magnetic resonance (NMR) spectroscopy was carried out using a Bruker AVIII or AVNEO instrument operating at 376 or 471 MHz using the stated solvent at around room temperature unless otherwise stated. Characteristic chemical shifts (δ) are given in parts-per-million. Preparative reverse phase HPLC was conducted using either a Waters FractionLynx preparative HPLC system (2525 pump, 2996/2998 UV/vis detector, 2767 liquid handler), a Gilson preparative HPLC system (322 pump, 155 UV/vis detector, GX-281 liquid handler), or a Gilson preparative HPLC system (322 pump, 151 UV/vis 163 spectrometer, 234 autoinjector, GX-271 liquid handler). The columns used for the preparative purification of compounds were a Waters Sunfire OBD, Phenomenex Luna Phenyl Hexyl, Waters Xbridge Phenyl, or Waters XBridge Prep OBD C18 column unless otherwise stated. Appropriate focused gradients were selected based on acetonitrile and methanol solvent systems under either acidic or basic conditions. The modifiers used under acidic/basic conditions were formic acid (0.1% v/v) or TFA (0.1% v/v) and ammonium bicarbonate (10 mM), respectively. A flow rate of 20 mL/min was used unless otherwise stated with UV monitoring at 254 nm. Supercritical Fluid Chromatography (SFC) was conducted using either a Waters Thar Prep100 preparative SFC system (P200 CO_2_ pump, 2545 modifier pump, 2998 UV/vis detector, 2767 liquid handler with Stacked Injection Module) or a Waters Thar Investigator semi preparative system (Waters Fluid Delivery Module, 2998 UV/vis detector, Waters Fraction Collection Module). Where the Waters 2767 liquid handler was used it acted as both autosampler and fraction collector. The compounds were purified using a YMC Amylose-C column. Appropriate isocratic methods were selected based on methanol, ethanol, or isopropanol solvent systems under unmodified conditions.

#### *Tert*-butyl 4-(4-(5-Bromo-4-methylpyridin-3-yl)­phenyl)­piperazine-1-carboxylate (**26**)

3,5-Dibromo-4-methylpyridine (**25**) (1500 mg, 5.98 mmol) was combined with 4-(4-*tert*-butoxycarbonylpiperazinyl)­phenylboronic acid pinacol ester (2100 mg, 5.41 mmol), potassium phosphate tribasic (3800 mg, 17.9 mmol), and Pd­(dppf)­Cl_2_ (450 mg, 0.544 mmol) in a mixture of 1,4-dioxane (8 mL) and water (2 mL). The mixture was sparged via N_2_ bubbling for 5 min, then stirred at 70 °C for 5 h. Upon cooling, the reaction mixture was filtered through Celite, eluting with ethyl acetate (3 × 10 mL). The filtrate was then washed with 1 M aq. HCl (30 mL) and the phases were separated. The organic layer was passed through a hydrophobic frit and concentrated *in vacuo*. The crude material was purified by silica gel flash chromatography eluting with 0–100% ethyl acetate in diethyl ether. Fractions containing product were combined and concentrated *in vacuo* to afford a yellow solid. Ten mL of 1:1 hexane/ether was added to the solid, the mixture was heated to 70 °C for 30 min, then cooled on ice for 2 h. The mixture was filtered and the solid dried under vacuum to afford *tert*-butyl 4-[4-(5-bromo-4-methyl-3-pyridyl)­phenyl]­piperazine-1-carboxylate (**26**) (1000 mg, 2.31 mmol, 39%) as a white solid. ^1^H NMR (500 MHz, CDCl_3_): δ 8.54 (s, 1H), 8.24 (s, 1H), 7.13 (d, *J* = 8.9 Hz, 2H), 6.91 (d, *J* = 8.9 Hz, 2H), 3.54 (t, *J* = 5.2 Hz, 4H), 3.15 (t, *J* = 5.2 Hz, 4H), 2.29 (s, 3H), 1.43 (s, 9H) ppm. ^13^C NMR (126 MHz, CDCl_3_): δ 154.7, 150.8, 149.9, 148.7, 144.4, 139.1, 130.3, 128.6, 124.4, 116.0, 80.0, 48.9, 31.6, 28.4, 20.4 ppm. LRMS (ES^+^): [M­(^79^Br)+H]^+^ 432.2 and [M­(^81^Br)+H]^+^ 434.2. HRMS: Calculated for C_21_H_27_BrN_3_O_2_ [M­(^79^Br)+H]^+^ 432.1287 and [M­(^81^Br)+H]^+^ 434.1266. Found 432.1284 and 434.1259. Error <2 ppm.

#### *Tert*-butyl 4-[4-[4-Methyl-5-(3,4,5-trimethoxyphenyl)-3-pyridyl]­phenyl]­piperazine-1-carboxylate (**27**)

*Tert*-butyl 4-[4-(5-bromo-4-methyl-3-pyridyl)­phenyl]­piperazine-1-carboxylate (**26**) (2500 mg, 5.78 mmol) was combined with 3,4,5-trimethoxyphenylboronic acid (1500 mg, 7.08 mmol), potassium phosphate tribasic (3750 mg, 17.7 mmol), and Pd­(dppf)­Cl_2_ (420 mg, 0.508 mmol) in a mixture of cyclopentyl methyl ether (5 mL) and water (1.25 mL), and the mixture was sparged via N_2_ bubbling for 10 min, then stirred at 80 °C for 4 h. Upon cooling, the mixture was filtered through Celite eluting with ethyl acetate (3 × 10 mL). The filtrate was washed with 1 M aq. HCl solution (20 mL) and the organic phase was passed through a hydrophobic frit and concentrated *in vacuo*. The crude material was purified by silica gel flash chromatography eluting with 0–100% ethyl acetate in diethyl ether. Fractions containing product were combined and concentrated *in vacuo* to afford *tert*-butyl 4-[4-[4-methyl-5-(3,4,5-trimethoxyphenyl)-3-pyridyl]­phenyl]­piperazine-1-carboxylate (**27**) (1700 mg, 3.27 mmol, 57%) as a cream colored solid. ^1^H NMR (400 MHz, *d*_6_-DMSO): δ 8.36 (s, 1H), 8.33 (s, 1H), 7.32 (d, *J* = 8.9 Hz, 2H), 7.07 (d, *J* = 8.9 Hz, 2H), 6.73 (s, 2H), 3.82 (s, 6H), 3.72 (s, 3H), 3.48 (t, *J* = 5.2 Hz, 4H), 3.19 (t, *J* = 5.2 Hz, 4H), 2.18 (s, 3H), 1.43 (s, 9H) ppm. ^13^C NMR (101 MHz, *d*_6_-DMSO): δ 154.4, 153.2, 150.7, 148.7, 148.2, 142.1, 138.1, 137.7, 137.4, 133.9, 130.6, 128.7, 116.0, 107.4, 79.5, 60.5, 56.5, 48.4, 28.5, 18.6 ppm. LRMS (ES^+^): [M + H]^+^ 520.3. HRMS: Calculated for C_30_H_38_N_3_O_5_ [M + H]^+^ 520.2811. Found 520.2802. Error <2 ppm.

#### *Tert*-butyl 4-(4-(5-(4-Fluoro-3,5-dimethoxyphenyl)-4-methylpyridin-3-yl)­phenyl)­piperazine-1-carboxylate (**28**)

*Tert*-butyl 4-[4-(5-bromo-4-methyl-3-pyridyl)­phenyl]­piperazine-1-carboxylate (**26**) (700 mg, 1.62 mmol) was combined with (4-fluoro-3,5-dimethoxyphenyl)­boronic acid (400 mg, 2.00 mmol), potassium phosphate tribasic (1050 mg, 4.95 mmol), and Pd­(dppf)­Cl_2_ (120 mg, 0.145 mmol) in a mixture of 1,4-dioxane (5 mL) and water (1.25 mL) and the mixture was sparged via N_2_ bubbling for 10 min, then stirred at 80 °C for 4 h. Upon cooling, the mixture was filtered through Celite eluting with ethyl acetate (3 × 10 mL). The filtrate was washed with 1 M aq. HCl solution (20 mL) and the organic phase was passed through a hydrophobic frit and concentrated *in vacuo*. The crude material was purified by silica gel flash chromatography eluting with 0–100% ethyl acetate in diethyl ether. Fractions containing product were combined and concentrated *in vacuo* to afford *tert*-butyl 4-[4-[5-(4-fluoro-3,5-dimethoxyphenyl)-4-methyl-3-pyridyl]­phenyl]­piperazine-1-carboxylate (**28**) (650 mg, 1.28 mmol, 79%) as a cream-colored solid. ^1^H NMR (400 MHz, CDCl_3_): δ 8.45 (s, 1H), 8.40 (s, 1H), 7.30 (d, *J* = 8.9 Hz, 2H), 7.03 (d, *J* = 8.9 Hz, 2H), 6.60 (d, *J* = 7.5 Hz, 2H), 3.93 (s, 6H), 3.64 (t, *J* = 5.2 Hz, 4H), 3.24 (t, *J* = 5.2 Hz, 4H), 2.20 (s, 3H), 1.52 (s, 9H) ppm. ^13^C NMR (126 MHz, CDCl_3_): δ 154.3, 150.1, 148.8, 147.8 (d, ^3^ *J*_C–F_ = 8.1 Hz), 147.7, 142.9 (d, ^1^ *J*_C–F_ = 245 Hz), 141.8, 137.2, 133.3 (d, ^4^ *J*_C–F_ = 5.1 Hz), 129.9, 128.9, 128.1 (d, ^2^ *J*_C–F_ = 45.5 Hz), 115.5, 106.8, 79.5, 56.2, 48.5, 29.8, 27.9, 17.5 ppm. ^19^F NMR (376 MHz, CDCl_3_) δ −159.48 (t, *J* = 7.5 Hz) ppm. LRMS (ES^+^): [M + H]^+^ 508.3. HRMS: Calculated for C_29_H_35_FN_3_O_4_ [M + H]^+^ 508.2612. Found 508.2604. Error <2 ppm.

#### 1-[4-[4-Methyl-5-(3,4,5-trimethoxyphenyl)-3-pyridyl]­phenyl]­piperazine (**6**)^21^

*Tert*-butyl 4-[4-[4-methyl-5-(3,4,5-trimethoxyphenyl)-3-pyridyl]­phenyl]­piperazine-1-carboxylate (**27**) (1670 mg, 3.21 mmol) was combined with 3 M HCl in CPME (6.0 mL, 18.0 mmol) in dichloromethane (6 mL) and the mixture was stirred at r.t. for 2 h. The mixture was concentrated *in vacuo* to afford 1-[4-[4-methyl-5-(3,4,5-trimethoxyphenyl)-3-pyridyl]­phenyl]­piperazine (**6**) (1300 mg, 2.94 mmol, 92%) as an orange solid. The product was used as such in the next step without further purification. LRMS (ES^+^): [M + H]^+^ 420.3. Analytical data consistent with literature data.^21^

#### 1-(4-(5-(4-Fluoro-3,5-dimethoxyphenyl)-4-methylpyridin-3-yl)­phenyl)­piperazine (**7**)^21^

*Tert*-butyl 4-[4-[5-(4-fluoro-3,5-dimethoxyphenyl)-4-methyl-3-pyridyl]­phenyl]­piperazine-1-carboxylate (**28**) (600 mg, 1.18 mmol) was combined with 4 M HCl in dioxane (1.3 mL, 5.14 mmol) in dichloromethane (1 mL) and the mixture was stirred at r.t. for 3 h. The mixture was concentrated *in vacuo* to afford 1-[4-[5-(4-fluoro-3,5-dimethoxyphenyl)-4-methyl-3-pyridyl]­phenyl]­piperazine (**7**) (460 mg, 1.13 mmol, 96%) as an orange solid. The product was used as such in the next step without subsequent purification. LRMS (ES^+^): [M + H]^+^ 408.3. Analytical data consistent with literature data.^21^

#### 2-(2-(2-(Benzyloxy)­ethoxy)­ethoxy)­ethyl methanesulfonate (**30**)

2-(2-(2-(Benzyloxy)­ethoxy)­ethoxy)­ethan-1-ol (**29**) (2500 mg, 10.4 mmol) was combined with Et_3_N (4.4 mL, 31.2 mmol) in DCM (7 mL), and the mixture was cooled to 0 °C, then methanesulfonyl chloride (1.0 mL, 13.5 mmol) was added dropwise. The mixture was allowed to warm to room temperature and was stirred under nitrogen for 24 h. The mixture was washed with sat. aq. Na_2_CO_3_, the layers were partitioned and separated, then the aqueous layer was washed with DCM. The combined organic layers were dried using Mg_2_SO_4_, then filtered through a hydrophobic frit, and concentrated *in vacuo* to afford 2-(2-(2-(benzyloxy)­ethoxy)­ethoxy)­ethyl methanesulfonate (**30**) (2581 mg, 8.10 mmol, 78%) as a yellow oil. The product was used as such in the next step without subsequent purification. ^1^H NMR (400 MHz, CDCl_3_): δ 7.34–7.27 (m, 5H), 4.56 (s, 2H), 4.40–4.31 (m, 2H), 3.79–3.73 (m, 2H), 3.68–3.63 (m, 8H), 3.03 (s, 3H) ppm.

#### 5-(2-(2-(2-(Benzyloxy)­ethoxy)­ethoxy)­ethoxy)-2-(2,6-dioxopiperidin-3-yl)­isoindoline-1,3-dione (**31**)

5-Hydroxythalidomide (1000 mg, 3.65 mmol) and K_2_CO_3_ (1512 mg, 10.9 mmol) were dissolved in anhydrous DMF (5 mL). To this was added a solution of 2-(2-(2-(benzyloxy)­ethoxy)­ethoxy)­ethyl methanesulfonate (**30**) (1161 mg, 3.65 mmol) in anhydrous DMF (5 mL) and the reaction mixture was stirred at 80 °C for 24 h under nitrogen. Upon cooling, the mixture was partitioned between ethyl acetate and sat aq. NH_4_Cl, and the phases were separated. The aqueous layer was then washed with additional ethyl acetate. The combined organic layers were dried using Mg_2_SO_4_ and passed through a hydrophobic frit, then concentrated *in vacuo.* The crude material was purified by silica gel flash chromatography eluting with 0–75% 3:1 EtOH in ethyl acetate in cyclohexane to afford 5-(2-(2-(2-(benzyloxy)­ethoxy)­ethoxy)­ethoxy)-2-(2,6-dioxopiperidin-3-yl)­isoindoline-1,3-dione (**31**) (333 mg, 0.671 mmol, 18%) as a yellow oil. ^1^H NMR (400 MHz, CDCl_3_): δ 8.07 (s, 1H), 7.76 (d, *J* = 8.3 Hz, 1H), 7.35 (d, *J* = 2.3 Hz, 1H), 7.34–7.28 (m, 5H), 7.21 (dd, *J* = 8.3, 2.3 Hz, 1H), 4.95 (dd, *J* = 12.4, 5.4 Hz, 1H), 4.57 (s, 2H), 4.25–4.22 (m, 2H), 3.92–3.89 (m, 2H), 3.75–3.72 (m, 2H), 3.71–3.68 (m, 4H), 3.66–3.63 (m, 2H), 2.98–2.82 (m, 2H), 2.82–2.68 (m, 2H) ppm. LRMS (ES^+^): [M + H]^+^ 497.3.

#### 2-(2,6-Dioxopiperidin-3-yl)-5-(2-(2-(2-hydroxyethoxy)­ethoxy)­ethoxy)­isoindoline-1,3-dione (**32**)

5-(2-(2-(2-(Benzyloxy)­ethoxy)­ethoxy)­ethoxy)-2-(2,6-dioxopiperidin-3-yl)­isoindoline-1,3-dione (**31**) (333 mg, 0.671 mmol) was dissolved in ethanol (10 mL). To the solution was added 10% Pd/C (136 mg, 0.671 mmol) and 1-methyl-1,4-cyclohexadiene (0.75 mL, 6.71 mmol), and the reaction mixture was stirred at 80 °C for 17 h under nitrogen. The reaction mixture was filtered, and the filtrate concentrated *in vacuo* to afford 2-(2,6-dioxopiperidin-3-yl)-5-(2-(2-(2-hydroxyethoxy)­ethoxy)­ethoxy)­isoindoline-1,3-dione (**32**) (221 mg, 0.544 mmol, 81%) as a green gum. The product was used as such in the next step without further purification. ^1^H NMR (400 MHz, *d*_6_-DMSO): δ 11.13 (s, 1H), 7.85 (d, *J* = 8.3 Hz, 1H), 7.47 (d, *J* = 2.3 Hz, 1H), 7.39 (dd, *J* = 8.3, 2.3 Hz, 1H), 5.13 (dd, *J* = 12.9, 5.3 Hz, 1H), 4.58 (t, *J* = 5.5 Hz, 1H), 4.36–4.30 (m, 2H), 3.80 (t, *J* = 5.5 Hz, 2H), 3.66–3.59 (m, 2H), 3.58–3.53 (m, 2H), 3.51–3.47 (m, 2H), 3.43 (ddd, *J* = 6.0, 4.6, 1.0 Hz, 2H), 2.96–2.84 (m, 1H), 2.68–2.54 (m, 2H), 2.11–2.02 (m, 1H) ppm. LRMS (ES^+^): [M + H]^+^ 407.

#### 2-(2,6-Dioxopiperidin-3-yl)-5-(2-(2-(2-(4-(4-(4-methyl-5-(3,4,5-trimethoxyphenyl)­pyridin-3-yl)­phenyl)­piperazin-1-yl)­ethoxy)­ethoxy)­ethoxy)­isoindoline-1,3-dione (**11**)

2-(2,6-Dioxopiperidin-3-yl)-5-(2-(2-(2-hydroxyethoxy)­ethoxy)­ethoxy)­isoindoline-1,3-dione (**32**) (220 mg, 0.541 mmol) was dissolved in DCM (5.5 mL) and Dess-Martin periodinane (344 mg, 0.812 mmol) was added in one portion and the mixture was stirred at r.t. for 3 h. The mixture was diluted with DCM, then washed with a 1:1 mixture of sat aq. NaHCO_3_ and 10% aq. Na_2_S_2_O_3_. The aqueous layer was washed twice with DCM, then the combined organic layers were passed through a phase separator and concentrated *in vacuo* to afford a yellow gum. The crude material was dissolved in DCM (3.5 mL) and 1-(4-(4-methyl-5-(3,4,5-trimethoxyphenyl)­pyridin-3-yl)­phenyl)­piperazine (**6**) (218 mg, 0.519 mmol) was added and the mixture stirred at r.t. for 10 min, then sodium triacetoxyborohydride (220 mg, 1.04 mmol) was added and the mixture stirred at r.t. for 17 h. The reaction mixture was purified by silica gel flash chromatography eluting with 0–10% DCM in MeOH. Fractions containing product were combined and concentrated *in vacuo*, then the material obtained was repurified using supercritical fluid chromatography eluting with 35–40% isopropanol (0.1% diethylamine)/CO_2_ to afford 2-(2,6-dioxopiperidin-3-yl)-5-(2-(2-(2-(4-(4-(4-methyl-5-(3,4,5-trimethoxyphenyl)­pyridin-3-yl)­phenyl)­piperazin-1-yl)­ethoxy)­ethoxy)­ethoxy)­isoindoline-1,3-dione (**11**) (45 mg, 0.0557 mmol, 10%) as an off-white solid. FTIR (neat): *v*_max_ 2949, 2835, 1716, 1616, 1588, 1454 cm^–1^. ^1^H NMR (400 MHz, *d*_6_-DMSO): δ 11.13 (s, 1H), 8.36 (s, 1H), 8.33 (s, 1H), 7.84 (d, *J* = 8.3 Hz, 1H), 7.48 (d, *J* = 2.3 Hz, 1H), 7.39 (dd, *J* = 8.3, 2.3 Hz, 1H), 7.29 (d, *J* = 8.9 Hz, 2H), 7.00 (d, *J* = 8.9 Hz, 2H), 6.73 (s, 2H), 5.12 (dd, *J* = 12.8, 5.4 Hz, 1H), 4.36–4.30 (m, 2H), 3.82 (s, 6H), 3.80 (s, 2H), 3.72 (s, 3H), 3.62 (q, *J* = 3.3 Hz, 2H), 3.59–3.53 (m, 4H), 3.21–3.12 (m, 4H), 2.95–2.77 (m, 2H), 2.60–2.55 (m, 4H), 2.53–2.52 (m, 2H), 2.19 (s, 3H), 2.07–1.96 (m, 2H) ppm. ^13^C NMR (101 MHz, *d*_6_-DMSO): δ 173.2, 170.4, 167.3, 164.5, 153.2, 150.8, 148.7, 148.1, 142.1, 138.1, 137.8, 137.3, 134.4, 133.9, 130.5, 128.1, 127.3, 125.8, 123.5, 121.4, 115.3, 109.4, 107.4, 70.4, 70.2, 69.8, 69.1, 69.0, 68.9, 60.5, 56.5, 53.6, 49.4, 48.3, 31.4, 22.5, 18.6 ppm. HRMS: Calculated for C_44_H_50_N_5_O_10_ [M + H]^+^ 808.3558, found 808.3539. Error <3 ppm. UPLC-MS: Retention time: 2.97 min, peak area: 98.72%.

#### *Tert*-butyl 2-[2-[2-(2-Benzyloxyethoxy)­ethoxy]­ethoxy]­acetate (**33**)

2-[2-(2-Benzyloxyethoxy)­ethoxy]­ethanol (**29**) (5000 mg, 20.8 mmol) was combined with potassium *tert*-butoxide (3500 mg, 31.2 mmol) in tetrahydrofuran (30 mL) and the mixture stirred at 45 °C for 30 min. The mixture was then cooled to 0 °C and a solution of *tert*-butyl bromoacetate (3.7 mL, 25.1 mmol) in tetrahydrofuran (30 mL) was added. The mixture was stirred at room temperature for 72 h. The mixture was then diluted with 1 M aq. HCl (20 mL) and extracted with DCM (2 × 10 mL). The combined organic layers were passed through a hydrophobic frit and concentrated *in vacuo*. The crude residue was purified by silica gel flash chromatography eluting with 20–100% ethyl acetate in cyclohexane to afford *tert*-butyl 2-[2-[2-(2-benzyloxyethoxy)­ethoxy]­ethoxy]­acetate (**33**) (3500 mg, 9.87 mmol, 47%) as an orange oil. ^1^H NMR (400 MHz, *d*_6_-DMSO): δ 7.39–7.30 (m, 5H), 4.51 (s, 2H), 4.00 (s, 2H), 3.59–3.51 (m, 12H), 1.44 (s, 9H) ppm. ^13^C NMR (101 MHz, *d*_6_-DMSO): δ 169.8, 139.0, 128.7, 128.0, 127.8, 81.1, 72.5, 70.33, 70.30, 70.2, 70.2, 69.6, 68.6, 28.2 ppm.

#### *Tert*-butyl 2-[2-[2-(2-Hydroxyethoxy)­ethoxy]­ethoxy]­acetate (**34**)

*Tert*-butyl 2-[2-[2-(2-benzyloxyethoxy)­ethoxy]­ethoxy]­acetate (**33**) (3500 mg, 9.87 mmol) was dissolved in methanol (70 mL) and run through an H-Cube using a 10% Pd/C cartridge in continuous flow, at a rate of 1 mL/min, 60 °C, and at atmospheric pressure for 2 h. The solution obtained was concentrated *in vacuo* to afford *tert*-butyl 2-[2-[2-(2-hydroxyethoxy)­ethoxy]­ethoxy]­acetate (**34**) (2500 mg, 7.57 mmol, 77%) as a colorless oil. This was used in the next step without further purification.^1^H NMR (400 MHz, *d*_6_-DMSO): δ 3.99 (s, 2H), 3.60–3.40 (m, 12H), 1.43 (s, 9H) ppm. (1 × exchangeable proton not observed).

#### *Tert*-butyl 2-(2-(2-(2-((Methylsulfonyl)­oxy)­ethoxy)­ethoxy)­ethoxy)­acetate (**35**)

*Tert*-butyl 2-[2-[2-(2-hydroxyethoxy)­ethoxy]­ethoxy]­acetate (**34**) (400 mg, 1.51 mmol) was dissolved in DCM (10 mL) and the mixture cooled to 0 °C, then Et_3_N (0.63 mL, 4.54 mmol) and methanesulfonyl chloride (0.15 mL, 1.97 mmol) were added. The mixture was allowed to warm to r.t. and stirred for 17 h. The mixture was washed with sat. aq. Na_2_CO_3_, the phases were partitioned and separated, and the aqueous layer was washed with DCM. The combined organic layers were passed through a hydrophobic frit and concentrated *in vacuo* to afford *tert*-butyl 2-(2-(2-(2-((methylsulfonyl)­oxy)­ethoxy)­ethoxy)­ethoxy)­acetate (**35**) (516 mg, 1.51 mmol, quant) as a green oil. The product was used as such in the next step without further purification. ^1^H NMR (400 MHz, CDCl_3_): δ 4.41–4.35 (m, 2H), 4.01 (s, 2H), 3.80–3.75 (m, 2H), 3.72–3.65 (m, 8H), 3.08 (s, 3H), 1.48 (s, 9H) ppm.

#### *Tert*-butyl 2-(2-(2-(2-(4-(4-(4-Methyl-5-(3,4,5-trimethoxyphenyl)­pyridin-3-yl)­phenyl)­piperazin-1-yl)­ethoxy)­ethoxy)­ethoxy)­acetate (**36**)

*Tert*-butyl 2-(2-(2-(2-((methylsulfonyl)­oxy)­ethoxy)­ethoxy)­ethoxy)­acetate (**35**) (413 mg, 1.21 mmol) was combined with 1-[4-[4-methyl-5-(3,4,5-trimethoxyphenyl)-3-pyridyl]­phenyl]­piperazine (**6**) (389 mg, 0.927 mmol), and K_2_CO_3_ (384 mg, 2.78 mmol) in MeCN (10 mL), and the mixture was stirred at 80 °C for 72 h. Upon cooling, the mixture was concentrated *in vacuo*, then partitioned between ethyl acetate and water. The phases were separated and the aqueous layer was washed three times with ethyl acetate. The combined organic layers were dried using Mg_2_SO_4_ and filtered through a hydrophobic frit, then concentrated *in vacuo*. The crude material was purified by silica gel flash chromatography eluting with 10–80% ethyl acetate in cyclohexane to afford *tert*-butyl 2-(2-(2-(2-(4-(4-(4-methyl-5-(3,4,5-trimethoxyphenyl)­pyridin-3-yl)­phenyl)­piperazin-1-yl)­ethoxy)­ethoxy)­ethoxy)­acetate (**36**) (485 mg, 0.728 mmol, 79%) as a yellow oil. ^1^H NMR (400 MHz, *d*_6_-DMSO): δ 8.41 (s, 1H), 8.39 (s, 1H), 7.28–7.26 (m, 2H), 7.00 (d, *J* = 8.8 Hz, 2H), 6.55 (s, 2H), 4.03 (s, 2H), 3.91 (s, 3H), 3.89 (s, 6H), 3.73–3.66 (m, 12H), 3.29 (t, *J* = 5.0 Hz, 4H), 2.73–2.68 (m, 4H), 1.48 (s, 9H) ppm. LRMS (ES^+^): [M + H]^+^ 666.5.

#### 2-(2-(2-(2-(4-(4-(4-Methyl-5-(3,4,5-trimethoxyphenyl)­pyridin-3-yl)­phenyl)­piperazin-1-yl)­ethoxy)­ethoxy)­ethoxy)­acetic Acid (**37**)

*Tert*-butyl 2-(2-(2-(2-(4-(4-(4-methyl-5-(3,4,5-trimethoxyphenyl)­pyridin-3-yl)­phenyl)­piperazin-1-yl)­ethoxy)­ethoxy)­ethoxy)­acetate (**36**) (485 mg, 0.728 mmol) was combined with 4 M HCl in dioxane (5.4 mL, 33.6 mmol) and the mixture stirred at r.t. for 1 h. The mixture was concentrated *in vacuo* to afford 2-(2-(2-(2-(4-(4-(4-methyl-5-(3,4,5-trimethoxyphenyl)­pyridin-3-yl)­phenyl)­piperazin-1-yl)­ethoxy)­ethoxy)­ethoxy)­acetic acid (**37**) (444 mg, 0.728 mmol, quant) as a light brown solid. The product was used as such in the next step without further purification. LRMS (ES^+^): [M + H]^+^ 610.

#### (2*S*,4*R*)-1-((*S*)-2-(*Tert*-butyl)-14-(4-(4-(4-methyl-5-(3,4,5-trimethoxyphenyl)­pyridin-3-yl)­phenyl)­piperazin-1-yl)-4-oxo-6,9,12-trioxa-3-azatetradecanoyl)-4-hydroxy-*N*-(4-(4-methylthiazol-5-yl)­benzyl)­pyrrolidine-2-carboxamide (**12**)

2-(2-(2-(2-(4-(4-(4-Methyl-5-(3,4,5-trimethoxyphenyl)­pyridin-3-yl)­phenyl)­piperazin-1-yl)­ethoxy)­ethoxy)­ethoxy)­acetic acid (**37**) (444 mg, 0.728 mmol) was dissolved in DCM (6 mL) and the mixture heated to 40 °C. Thionyl chloride (0.23 mL, 3.20 mmol) was added and the mixture was stirred at 40 °C for 3 h. The reaction mixture was concentrated *in vacuo* to afford a brown oil. A solution of the crude acyl chloride (200 mg, 0.318 mmol) in DMF (1.5 mL) was added to a solution of (2*S*,4*R*)-1-((*S*)-2-amino-3,3-dimethylbutanoyl)-4-hydroxy-*N*-(4-(4-methylthiazol-5-yl)­benzyl)­pyrrolidine-2-carboxamide hydrochloride (164 mg, 0.350 mmol) and Et_3_N (0.11 mL, 0.796 mmol) in DMF (1.5 mL), and the mixture was stirred at r.t. for 17 h. The mixture was concentrated *in vacuo*, and the residue obtained was partitioned between ethyl acetate and water. The phases were separated and the aqueous phase was washed five times with ethyl acetate. The combined organic layers were dried using Mg_2_SO_4_, filtered through a hydrophobic frit, and concentrated *in vacuo*. The crude material was purified by reverse phase preparative HPLC eluting with 20–80% MeOH in water + 0.1% formic acid, to afford (2*S*,4*R*)-1-((*S*)-2-(*tert*-butyl)-14-(4-(4-(4-methyl-5-(3,4,5-trimethoxyphenyl)­pyridin-3-yl)­phenyl)­piperazin-1-yl)-4-oxo-6,9,12-trioxa-3-azatetradecanoyl)-4-hydroxy-*N*-(4-(4-methylthiazol-5-yl)­benzyl)­pyrrolidine-2-carboxamide (**12**) (14 mg, 0.0137 mmol, 4%) as a white solid. FTIR (neat): *v*_max_ 2947, 2878, 1644, 1636, 1618, 1519, 1508, 1458 cm^–1^. ^1^H NMR (400 MHz, *d*_6_-DMSO): δ 8.98 (s, 1H), 8.60 (t, *J* = 6.1 Hz, 1H), 8.35 (s, 1H), 8.32 (s, 1H), 7.43 (d, *J* = 9.3 Hz, 2H), 7.40 (s, 2H), 7.38 (s, 1H), 7.29 (d, *J* = 8.9 Hz, 2H), 7.03 (d, *J* = 8.9 Hz, 2H), 6.72 (s, 2H), 4.57 (d, *J* = 9.6 Hz, 1H), 4.43 (dd, *J* = 7.1, 3.5 Hz, 1H), 4.41–4.30 (m, 2H), 4.24 (dd, *J* = 15.8, 5.6 Hz, 1H), 3.98 (s, 2H), 3.81 (s, 6H), 3.72 (s, 3H), 3.68–3.66 (m, 1H) 3.66–3.49 (m, 12H), 3.18 (t, *J* = 5.3 Hz, 4H), 2.56 (t, *J* = 5.3 Hz, 4H), 2.44 (s, 3H), 2.18 (s, 3H), 2.13–1.98 (m, 2H), 1.90 (ddd, *J* = 12.9, 8.9, 4.6 Hz, 1H), 0.95 (s, 9H) ppm. (1 × exchangeable proton not observed). ^13^C NMR (101 MHz, *d*_6_-DMSO): δ 172.2, 169.6, 169.1, 153.2, 151.7, 150.8, 148.2, 148.1, 144.3, 142.1, 139.9, 138.1, 137.4, 133.9, 131.6, 130.5, 130.2, 129.2, 127.9, 124.3, 115.4, 107.4, 99.1, 71.0, 70.3, 70.2, 70.1, 69.3, 68.9, 66.6, 60.5, 59.2, 57.7, 56.5, 53.6, 49.3, 48.3, 45.0, 36.2, 26.7, 19.9, 18.6, 16.4, 12.9 ppm. HRMS: Calculated for C_55_H_72_N_7_O_10_S [M + H]^+^ 1022.5061, found 1022.5021. Error <4 ppm. UPLC-MS: Retention time: 4.57 min, peak area: 98.51%.

#### 1-[4-[4-Methyl-5-(3,4,5-trimethoxyphenyl)-3-pyridyl]­phenyl]-4-(4-piperidylmethyl)­piperazine (**38**)

1-[4-[4-Methyl-5-(3,4,5-trimethoxyphenyl)-3-pyridyl]­phenyl]­piperazine (**6**) (1200 mg, 2.86 mmol) was combined with 1-boc-4-bromomethylpiperidine (1000 mg, 3.59 mmol) and potassium carbonate (1000 mg, 7.24 mmol) in *N,N*-dimethylformamide (3 mL) and the mixture was stirred at 120 °C for 17 h. Upon cooling, the mixture was concentrated *in vacuo* to remove the solvent, then redissolved in DCM (5 mL) and washed with water (5 mL). The organic phase was separated, then passed through a hydrophobic frit and concentrated *in vacuo*. The crude residue was dissolved in DCM (1 mL) and treated with 4 M HCl in dioxane (3 mL, 12.0 mmol) and the mixture stirred at r.t. for 2 h, then concentrated *in vacuo* to afford 1-[4-[4-methyl-5-(3,4,5-trimethoxyphenyl)-3-pyridyl]­phenyl]-4-(4-piperidylmethyl)­piperazine (**38**) (800 mg, 1.55 mmol, 54%) as an orange solid. The product was used as such in the next step without further purification. LRMS (ES^+^): [M + H]^+^ 517.3.

#### (4-(4-(4-Methyl-5-(3,4,5-trimethoxyphenyl)­pyridin-3-yl)­phenyl)­piperazin-1-yl)­(piperidin-4-yl)­methanone (**39**)

1-Boc-piperidine-4-carboxylic acid (100 mg, 0.436 mmol) was stirred with propylphosphonic anhydride solution 50% in EtOAc (50%, 0.35 mL, 0.588 mmol) and triethylamine (0.20 mL, 1.43 mmol) in *N,N*-dimethylformamide (1 mL) for 30 min at 50 °C, then 1-[4-[4-methyl-5-(3,4,5-trimethoxyphenyl)-3-pyridyl]­phenyl]­piperazine (**6**) (150 mg, 0.358 mmol) was added and the mixture stirred at 50 °C for 3 h. The mixture was concentrated *in vacuo* to remove the solvent, then redissolved in EtOAc (10 mL) and washed sequentially with sat. aq. Na_2_CO_3_ (5 mL), 5% HCl (5 mL), and sat. aq. Na_2_CO_3_ (5 mL). The organic layer was passed through a hydrophobic frit and concentrated *in vacuo* to afford an orange gum. The crude material was redissolved in dichloromethane (1 mL) and treated with 4 M HCl in dioxane (0.56 mL, 2.26 mmol) and the mixture was stirred at r.t. for 17 h, then concentrated *in vacuo* to afford [4-[4-[4-methyl-5-(3,4,5-trimethoxyphenyl)-3-pyridyl]­phenyl]­piperazin-1-yl]-(4-piperidyl)­methanone (**39**) (140 mg, 0.264 mmol, 74%) as a yellow gum. The product was used as such in the next step without further purification. LRMS (ES^+^): [M + H]^+^ 531.3.

#### (4-Fluoropiperidin-4-yl)­(4-(4-(4-methyl-5-(3,4,5-trimethoxyphenyl)­pyridin-3-yl)­phenyl)­piperazin-1-yl)­methanone (**40**)

1-(*Tert*-butoxycarbonyl)-4-fluoropiperidine-4-carboxylic acid was stirred with T3P (50% in EtOAc) (0.35 mL, 0.588 mmol) and triethylamine (0.2 mL, 1.43 mmol) in *N,N*-dimethylformamide (1 mL) for 30 min at 50 °C, then 1-[4-[4-methyl-5-(3,4,5-trimethoxyphenyl)-3-pyridyl]­phenyl]­piperazine (**6**) (150 mg, 0.358 mmol) was added and the mixture stirred at 50 °C for 3 h. The mixture was concentrated *in vacuo* to remove the solvent, then redissolved in EtOAc (10 mL) and washed sequentially with sat. aq. Na_2_CO_3_ (5 mL), 5% HCl (5 mL), and sat. aq. Na_2_CO_3_ (5 mL). The organic layer was passed through a hydrophobic frit and concentrated *in vacuo* to afford an orange gum. The crude material was redissolved in dichloromethane (1 mL) and treated with 4 M HCl in dioxane (0.5 mL, 2.00 mmol) and the mixture was stirred at r.t. for 17 h, then concentrated *in vacuo* to afford (4-fluoro-4-piperidyl)-[4-[4-[4-methyl-5-(3,4,5-trimethoxyphenyl)-3-pyridyl]­phenyl]­piperazin-1-yl]­methanone (**40**) (120 mg, 0.219 mmol, 61%) as a yellow gum. The product was used as such in the next step without subsequent purification. LRMS (ES^+^): [M + H]^+^ 549.3.

#### Azetidin-3-yl­(4-(4-(4-methyl-5-(3,4,5-trimethoxyphenyl)­pyridin-3-yl)­phenyl)­piperazin-1-yl)­methanone (**41**)

1-Boc-azetidine-3-carboxylic acid (90 mg, 0.447 mmol) was stirred with T3P (50% in EtOAc) (0.35 mL, 0.588 mmol) and triethylamine (0.20 mL, 1.43 mmol) in *N,N*-dimethylformamide (1 mL) for 30 min at 50 °C, then 1-[4-[4-methyl-5-(3,4,5-trimethoxyphenyl)-3-pyridyl]­phenyl]­piperazine (**6**) (150 mg, 0.358 mmol) was added and the mixture stirred at 50 °C for 3 h. The mixture was concentrated *in vacuo* to remove the solvent, then redissolved in EtOAc (10 mL) and washed sequentially with sat. aq. Na_2_CO_3_ (5 mL), 5% HCl (5 mL), and sat. aq. Na_2_CO_3_ (5 mL). The organic layer was passed through a hydrophobic frit and concentrated *in vacuo* to afford an orange gum. The crude material was redissolved in dichloromethane (1 mL) and treated with 4 M HCl in dioxane (0.50 mL, 2.00 mmol) and the mixture was stirred at r.t., for 17 h, then concentrated *in vacuo* to afford azetidin-3-yl-[4-[4-[4-methyl-5-(3,4,5-trimethoxyphenyl)-3-pyridyl]­phenyl]­piperazin-1-yl]­methanone (**41**) (80 mg, 0.159 mmol, 45%) as a yellow gum. The product was used as such in the next step without further purification. LRMS (ES^+^): [M + H]^+^ 503.3.

#### 1-(4-(5-(4-Fluoro-3,5-dimethoxyphenyl)-4-methylpyridin-3-yl)­phenyl)-4-(piperidin-4-ylmethyl)­piperazine (**42**)

1-[4-[5-(4-Fluoro-3,5-dimethoxyphenyl)-4-methyl-3-pyridyl]­phenyl]­piperazine (**7**) (300 mg, 0.736 mmol) was combined with 1-boc-4-bromomethylpiperidine (220 mg, 0.791 mmol) and potassium carbonate (260 mg, 1.88 mmol) in *N,N*-dimethylformamide (3 mL) and the mixture was stirred at 120 °C for 17 h. The mixture was concentrated *in vacuo* to remove the solvent, then redissolved in DCM (5 mL) and washed with water (5 mL). The organic phase was separated, passed through a hydrophobic frit, and concentrated *in vacuo* to afford a yellow gum. This was redissolved in dichloromethane (1 mL) and treated with 4 M HCl in dioxane (0.83 mL, 3.33 mmol) and the mixture was stirred at r.t. for 2 h. The mixture was concentrated *in vacuo* to afford 1-[4-[5-(4-fluoro-3,5-dimethoxyphenyl)-4-methyl-3-pyridyl]­phenyl]-4-(4-piperidylmethyl)­piperazine (**42**) (170 mg, 0.337 mmol, 46%) as an orange solid. The product was used as such in the next step without further purification. LRMS (ES^+^): [M + H]^+^ 504.3.

#### (4-(4-(5-(4-Fluoro-3,5-dimethoxyphenyl)-4-methylpyridin-3-yl)­phenyl)­piperazin-1-yl)­(piperidin-4-yl)­methanone (**43**)

1-Boc-piperidine-4-carboxylic acid (70 mg, 0.305 mmol) was stirred with T3P (50% in EtOAc) (0.25 mL, 0.420 mmol) and triethylamine (0.15 mL, 1.08 mmol) in *N,N*-dimethylformamide (1 mL) for 30 min at 50 °C, then 1-[4-[5-(4-fluoro-3,5-dimethoxyphenyl)-4-methyl-3-pyridyl]­phenyl]­piperazine (**7**) (100 mg, 0.245 mmol) was added and the mixture stirred at 50 °C for 3 h. The mixture was concentrated *in vacuo* to remove the solvent, then redissolved in EtOAc (10 mL) and washed sequentially with sat. aq. Na_2_CO_3_ (5 mL), 5% HCl (5 mL), and sat. aq. Na_2_CO_3_ (5 mL). The organic layer was passed through a hydrophobic frit and concentrated *in vacuo*. The crude material was redissolved in dichloromethane (1 mL) and treated with 4 M HCl in dioxane (0.35 mL, 1.40 mmol) and the mixture was stirred at r.t. for 17 h, then concentrated *in vacuo* to afford [4-[4-[5-(4-fluoro-3,5-dimethoxyphenyl)-4-methyl-3-pyridyl]­phenyl]­piperazin-1-yl]-(4-piperidyl)­methanone (**43**) (100 mg, 0.193 mmol, 79%) as a yellow gum. The product was used as such in the next step without subsequent purification. LRMS (ES^+^): [M + H]^+^ 518.3.

#### 1-(4-(5-(4-Fluoro-3,5-dimethoxyphenyl)-4-methylpyridin-3-yl)­phenyl)-4-(pyrrolidin-3-ylmethyl)­piperazine (**44**)

1-[4-[5-(4-Fluoro-3,5-dimethoxyphenyl)-4-methyl-3-pyridyl]­phenyl]­piperazine (**7**) (200 mg, 0.491 mmol) was combined with *tert*-butyl 3-(bromomethyl)­pyrrolidine-1-carboxylate (135 mg, 0.511 mmol) and potassium carbonate (170 mg, 1.23 mmol) in *N,N*-dimethylformamide (3 mL) and the mixture was stirred at 120 °C for 17 h. The mixture was then concentrated *in vacuo* to remove the solvent, then redissolved in DCM (5 mL) and washed with water (5 mL). The organic phase was separated, passed through a hydrophobic frit, and concentrated *in vacuo* to afford a yellow gum. The crude material was redissolved in dichloromethane (1 mL) and treated with 3 M HCl in CPME (1.0 mL, 3.00 mmol), and the mixture was stirred at r.t. for 2 h. The mixture was then concentrated *in vacuo* to afford 1-[4-[5-(4-fluoro-3,5-dimethoxyphenyl)-4-methyl-3-pyridyl]­phenyl]-4-(pyrrolidin-3-ylmethyl)­piperazine (**44**) (100 mg, 0.204 mmol, 42%) as an orange solid. The product was used as such in the next step without subsequent purification. LRMS (ES^+^): [M + H]^+^ 491.2.

#### (4-(4-(5-(4-Fluoro-3,5-dimethoxyphenyl)-4-methylpyridin-3-yl)­phenyl)­piperazin-1-yl)­(pyrrolidin-3-yl)­methanone (**45**)

*N*-Boc-pyrrolidine-3-carboxylic acid (100 mg, 0.465 mmol) was stirred with HATU (300 mg, 0.789 mmol) and triethylamine (0.30 mL, 2.15 mmol) in *N,N*-dimethylformamide (2 mL) for 30 min at 50 °C, then 1-[4-[5-(4-fluoro-3,5-dimethoxyphenyl)-4-methyl-3-pyridyl]­phenyl]­piperazine (**7**) (150 mg, 0.368 mmol) was added and the mixture stirred at 50 °C for 3 h. The mixture was concentrated *in vacuo* to remove the solvent, then redissolved in EtOAc (10 mL) and washed sequentially with sat. aq. Na_2_CO_3_ (5 mL), 5% HCl (5 mL), and sat. aq. Na_2_CO_3_ (5 mL). The organic layer was passed through a hydrophobic frit and concentrated *in vacuo* to afford an orange gum. The crude material was redissolved in dichloromethane (2 mL) and treated with 3 M HCl in CPME (0.70 mL, 2.10 mmol) and the mixture was stirred at r.t. for 17 h, then concentrated *in vacuo* to afford [4-[4-[5-(4-fluoro-3,5-dimethoxyphenyl)-4-methyl-3-pyridyl]­phenyl]­piperazin-1-yl]­pyrrolidin-3-ylmethanone (**45**) (100 mg, 0.198 mmol, 54%) as a yellow gum. The material was used as such without further purification. LRMS (ES^+^): [M + H]^+^ 505.3.

#### 2-(2,6-Dioxo-3-piperidyl)-5-[4-[[4-[4-[4-methyl-5-(3,4,5-trimethoxyphenyl)-3-pyridyl]­phenyl]­piperazin-1-yl]­methyl]-1-piperidyl]­isoindoline-1,3-dione (**13**)

1-[4-[4-Methyl-5-(3,4,5-trimethoxyphenyl)-3-pyridyl]­phenyl]-4-(4-piperidylmethyl)­piperazine (**38**) (500 mg, 0.968 mmol) was combined with 2-(2,6-dioxo-3-piperidyl)-5-fluoroisoindoline-1,3-dione (350 mg, 1.27 mmol) and triethylamine (0.50 mL, 3.59 mmol) in *N,N*-dimethylformamide (0.5 mL) and the mixture was stirred at 100 °C for 17 h. The mixture was concentrated *in vacuo* to remove the solvent, then redissolved in THF (10 mL) and washed with 1 M aq. HCl solution (10 mL). The THF layer was discarded, and the aqueous layer was extracted with DCM (3 × 5 mL). The combined organic layers were passed through a hydrophobic frit and concentrated *in vacuo*. The crude material was purified by reverse phase preparative HPLC eluting with 5–95% MeCN in water + 0.1% v/v TFA to afford 2-(2,6-dioxo-3-piperidyl)-5-[4-[[4-[4-[4-methyl-5-(3,4,5-trimethoxyphenyl)-3-pyridyl]­phenyl]­piperazin-1-yl]­methyl]-1-piperidyl]­isoindoline-1,3-dione (**13**) (100 mg, 0.129 mmol, 13%) as a light green solid. FTIR (neat): *v*_max_ 3385, 2925, 2854, 1768, 1709, 1616, 1508, 1465, 1458 cm^–1^. ^1^H NMR (400 MHz, CDCl_3_): δ 8.42 (s, 1H), 8.40 (s, 1H), 8.15 (s, 1H), 7.68 (d, *J* = 8.5 Hz, 1H), 7.31–7.27 (m, 3H), 7.06 (dd, *J* = 8.5, 2.4 Hz, 1H), 7.01 (d, *J* = 8.8 Hz, 2H), 6.55 (s, 2H), 4.94 (dd, *J* = 12.4, 5.3 Hz, 1H), 3.97 (d, *J* = 12.7 Hz, 2H), 3.92 (s, 3H), 3.89 (s, 6H), 3.29 (t, *J* = 4.6 Hz, 4H), 2.99 (t, *J* = 11.7 Hz, 2H), 2.94–2.81 (m, 2H), 2.83–2.69 (m, 2H), 2.63 (d, *J* = 8.2 Hz, 4H), 2.31 (d, *J* = 7.0 Hz, 2H), 2.21 (s, 3H), 2.16–2.10 (m, 1H), 1.94 (d, *J* = 12.7 Hz, 2H), 1.32 (q, *J* = 11.7 Hz, 2H) ppm. ^13^C NMR (101 MHz, CDCl_3_): δ 170.7, 168.0, 167.6, 166.8, 154.9, 152.6, 150.1, 148.5, 147.6, 141.9, 137.6, 137.3, 137.0, 133.9, 133.5, 129.8, 128.4, 124.9, 118.1, 117.3, 114.9, 108.1, 106.3, 63.8, 60.5, 55.7, 53.1, 48.6, 48.2, 47.6, 32.8, 31.0, 29.6, 22.3, 17.6 ppm. HRMS: Calculated for C_44_H_49_N_6_O_7_ [M + H]^+^ 773.3663, found 773.3628. Error <5 ppm. UPLC-MS: Retention time: 3.04 min, peak area: 99.22%.

#### 2-(2,6-Dioxopiperidin-3-yl)-5-(4-(4-(4-(4-methyl-5-(3,4,5-trimethoxyphenyl)­pyridin-3-yl)­phenyl)­piperazine-1-carbonyl)­piperidin-1-yl)­isoindoline-1,3-dione (**14**)

2-(2,6-Dioxo-3-piperidyl)-5-fluoroisoindoline-1,3-dione (42 mg, 0.152 mmol) was combined with [4-[4-[4-methyl-5-(3,4,5-trimethoxyphenyl)-3-pyridyl]­phenyl]­piperazin-1-yl]-(4-piperidyl)­methanone (**39**) (70 mg, 0.132 mmol) and triethylamine (0.1 mL, 0.717 mmol) in *N,N*-dimethylformamide (0.5 mL) and the mixture was stirred at 100 °C for 17 h. Upon cooling, the mixture was concentrated *in vacuo* to remove the solvent, then redissolved in ethyl acetate (10 mL) and washed with 1 M aq. HCl solution (10 mL). The organic layer was discarded, and the aqueous layer was basified to pH 9 with sat. aq. Na_2_CO_3_ solution, then extracted with THF (3 × 5 mL). The combined organic layers were passed through a hydrophobic frit and concentrated *in vacuo*. The crude material was purified by reverse phase preparative HPLC eluting with 5–95% MeCN in water + 0.1% v/v TFA to afford 2-(2,6-dioxo-3-piperidyl)-5-[4-[4-[4-[4-methyl-5-(3,4,5-trimethoxyphenyl)-3-pyridyl]­phenyl]­piperazine-1-carbonyl]-1-piperidyl]­isoindoline-1,3-dione (**14**) (9.0 mg, 0.0114 mmol, 9%) as a light green solid. FTIR (neat): *v*_max_ 3069, 2967, 1718, 1620, 1509, 1464 cm^–1^. ^1^H NMR (400 MHz, CDCl_3_): δ 8.62 (s, 1H), 8.61 (s, 1H), 8.04 (s, 1H), 7.72 (d, *J* = 8.5 Hz, 1H), 7.37–7.30 (m, 3H), 7.12–7.09 (m, 1H), 7.07 (d, *J* = 8.8 Hz, 2H), 6.56 (s, 2H), 4.97 (dd, *J* = 12.4, 5.2 Hz, 1H), 4.03 (d, *J* = 13.1 Hz, 2H), 3.95 (s, 3H), 3.92 (s, 6H), 3.87 (s, 2H), 3.79 (s, 2H), 3.36 (d, *J* = 16.1 Hz, 4H), 3.11 (t, *J* = 11.0 Hz, 2H), 2.95–2.86 (m, 2H), 2.85–2.74 (m, 2H), 2.46 (s, 3H), 2.20–2.13 (m, 1H), 2.05–1.96 (m, 2H), 1.92 (d, *J* = 10.5 Hz, 2H) ppm. ^13^C NMR (101 MHz, CDCl_3_): δ 172.3, 170.4, 167.7, 167.4, 166.7, 154.8, 153.3, 150.7, 148.7, 141.3, 140.9, 139.2, 138.6, 138.5, 133.9, 129.8, 129.3, 125.0, 118.9, 117.7, 115.7, 111.7, 108.3, 105.9, 60.5, 55.9, 48.7, 46.9, 41.1, 40.0, 37.4, 30.9, 27.3, 22.3, 19.2 ppm. HRMS: Calculated for C_44_H_47_N_6_O_8_ [M + H]^+^ 787.3455, found 787.3421. Error <5 ppm. HPLC: Retention time: 9.311 min, peak area: 100.00%.

#### 2-(2,6-Dioxopiperidin-3-yl)-5-(4-fluoro-4-(4-(4-(4-methyl-5-(3,4,5-trimethoxyphenyl)­pyridin-3-yl)­phenyl)­piperazine-1-carbonyl)­piperidin-1-yl)­isoindoline-1,3-dione (**15**)

2-(2,6-Dioxo-3-piperidyl)-5-fluoroisoindoline-1,3-dione (35 mg, 0.127 mmol) was combined with (4-fluoro-4-piperidyl)-[4-[4-[4-methyl-5-(3,4,5-trimethoxyphenyl)-3-pyridyl]­phenyl]­piperazin-1-yl]­methanone (**40**) (60 mg, 0.109 mmol) and triethylamine (0.1 mL, 0.717 mmol) in *N,N*-dimethylformamide (0.5 mL) and the mixture was stirred at 100 °C for 17 h. The mixture was concentrated *in vacuo* to remove the solvent, then redissolved in ethyl acetate (10 mL) and washed with 1 M aq. HCl solution (10 mL). The organic layer was discarded, and the aqueous layer was basified to pH 9 with sat. aq. Na_2_CO_3_ solution, then extracted with THF (3 × 5 mL). The combined organic layers were passed through a hydrophobic frit and concentrated *in vacuo*. The crude material was purified by reverse phase preparative HPLC eluting with 5–95% MeCN in water + 0.1% v/v TFA to afford 2-(2,6-dioxo-3-piperidyl)-5-[4-fluoro-4-[4-[4-[4-methyl-5-(3,4,5-trimethoxyphenyl)-3-pyridyl]­phenyl]­piperazine-1-carbonyl]-1-piperidyl]­isoindoline-1,3-dione (**15**) (9.0 mg, 0.0112 mmol, 10%) as a light green solid. FTIR (neat): *v*_max_ 2981, 1717, 1620, 1514, 1456, 1396 cm^–1^. ^1^H NMR (400 MHz, CDCl_3_): δ 8.60 (s, 2H), 8.13 (s, br, 1H), 7.73 (d, *J* = 8.5 Hz, 1H), 7.37–7.29 (m, 3H), 7.13 (dd, *J* = 8.5, 2.4 Hz, 1H), 7.07 (d, *J* = 8.8 Hz, 2H), 6.56 (s, 2H), 4.97 (dd, *J* = 12.4, 5.3 Hz, 1H), 4.07–4.04 (m, 2H), 3.95 (s, 3H), 3.92 (s, 6H), 3.90–3.86 (m, 4H), 3.44 (t, *J* = 11.0 Hz, 2H), 3.41–3.31 (m, 4H), 2.98–2.83 (m, 2H), 2.84–2.69 (m, 2H), 2.46 (s, 3H), 2.40–2.27 (m, 2H), 2.19–2.11 (m, 2H) ppm. ^13^C NMR (101 MHz, CDCl_3_): δ 170.5, 168.1 (d, ^2^ *J*_C–F_ = 21.2 Hz), 167.7, 167.3, 166.6, 154.3, 153.3, 150.7, 141.2, 140.9, 140.2, 139.2, 138.54, 138.46, 133.9, 129.8, 129.4, 125.0, 124.9, 119.2, 117.7, 115.5, 108.4, 105.9, 95.0 (d, ^1^ *J*_C–F_ = 188.4 Hz), 60.9 (d, ^3^ *J*_C–F_ = 10.8 Hz), 60.5, 55.9, 48.7, 43.0, 32.0 (d, ^2^ *J*_C–F_ = 22.0 Hz), 30.9, 22.2, 19.2, 18.3 ppm. ^19^F NMR (376 MHz, CDCl_3_): δ −75.8 ppm. HRMS: Calculated for C_44_H_46_N_6_O_8_F [M + H]^+^ 805.3361, found 805.3321. Error <5 ppm. HPLC: Retention time: 4.509 min, peak area: 95.14%.

#### 2-(2,6-Dioxopiperidin-3-yl)-5-(3-(4-(4-(4-methyl-5-(3,4,5-trimethoxyphenyl)­pyridin-3-yl)­phenyl)­piperazine-1-carbonyl)­azetidin-1-yl)­isoindoline-1,3-dione (**16**)

2-(2,6-Dioxo-3-piperidyl)-5-fluoroisoindoline-1,3-dione (35 mg, 0.127 mmol) was combined with azetidin-3-yl­(4-(4-(4-methyl-5-(3,4,5-trimethoxyphenyl)­pyridin-3-yl)­phenyl)­piperazin-1-yl)­methanone (**41**) (60 mg, 0.109 mmol) and triethylamine (0.10 mL, 0.717 mmol) in *N,N*-dimethylformamide (0.5 mL) and the mixture was stirred at 100 °C for 17 h. The mixture was concentrated *in vacuo* to remove the solvent, then redissolved in ethyl acetate (10 mL) and washed with 1 M aq. HCl solution (10 mL). The organic layer was discarded, and the aqueous layer was basified to pH 9 with sat. aq. Na_2_CO_3_ solution, then extracted with THF (3 × 5 mL). The combined organic layers were passed through a hydrophobic frit and concentrated *in vacuo*. The crude material was purified by reverse phase preparative HPLC eluting with 5–95% MeCN in water + 0.1% v/v TFA to afford 2-(2,6-dioxo-3-piperidyl)-5-[3-[4-[4-[4-methyl-5-(3,4,5-trimethoxyphenyl)-3-pyridyl]­phenyl]­piperazine-1-carbonyl]­azetidin-1-yl]­isoindoline-1,3-dione (**16**) (13 mg, 0.0171 mmol, 14%) as a light green solid. FTIR (neat): *v*_max_ 2972, 1712, 1619, 1514, 1455, 1384 cm^–1^. ^1^H NMR (400 MHz, CDCl_3_): δ 8.61 (s, 1H), 8.59 (s, 1H), 8.27 (s, br, 1H), 7.67 (d, *J* = 8.3 Hz, 1H), 7.33 (d, *J* = 8.9 Hz, 2H), 7.07 (d, *J* = 8.9 Hz, 2H), 6.83 (d, *J* = 2.3 Hz, 1H), 6.59 (dd, *J* = 8.3, 2.3 Hz, 1H), 6.56 (s, 2H), 4.95 (dd, *J* = 12.3, 5.4 Hz, 1H), 4.33–4.29 (m, 2H), 3.95 (s, 3H), 3.91 (s, 6H), 3.91–3.85 (m, 4H), 3.61–3.58 (m, 2H), 3.38–3.31 (m, 4H), 2.96–2.82 (m, 2H), 2.82–2.68 (m, 2H), 2.46 (s, 3H), 2.18–2.11 (m, 1H) ppm. ^13^C NMR (101 MHz, CDCl_3_): δ 170.6, 168.9, 167.8, 167.3, 166.9, 154.3, 153.3, 153.1, 150.6, 141.2, 140.9, 139.3, 138.6, 138.4, 133.7, 129.8, 129.4, 125.3, 124.8, 118.2, 115.8, 113.9, 105.9, 104.6, 60.5, 55.9, 53.2, 48.6, 44.5, 41.3, 31.5, 30.9, 22.3, 19.2 ppm. HRMS: Calculated for C_42_H_43_N_6_O_8_ [M + H]^+^ 759.3142, found 759.3113. Error <4 ppm. HPLC: Retention time: 4.781 min, peak area: 95.94%.

#### 1-[2-Methoxy-5-[4-[4-[4-methyl-5-(3,4,5-trimethoxyphenyl)-3-pyridyl]­phenyl]­piperazine-1-carbonyl]­phenyl]­hexahydropyrimidine-2,4-dione (**17**)

3-(2,4-Dioxohexahydropyrimidin-1-yl)-4-methoxybenzoic acid (35 mg, 0.132 mmol) was stirred with *N,N*-diisopropylethylamine (0.10 mL, 0.574 mmol) and propylphosphonic anhydride solution 50% in EtOAc (0.15 mL, 0.252 mmol) in tetrahydrofuran (0.5 mL) at 50 °C for 30 min, then 1-[4-[4-methyl-5-(3,4,5-trimethoxyphenyl)-3-pyridyl]­phenyl]­piperazine (**6**) (50 mg, 0.119 mmol) was added. The mixture was stirred at 50 °C for 17 h. Upon cooling, the mixture was diluted with ethyl acetate (10 mL) and washed sequentially with sat. aq. Na_2_CO_3_ (5 mL), 5% HCl (5 mL), and sat. aq. Na_2_CO_3_ (5 mL). The organic layer was passed through a hydrophobic frit and concentrated *in vacuo*. The crude material was purified by reverse phase preparative HPLC eluting with 5–95% MeCN in water + 0.1% v/v TFA to afford 1-[2-methoxy-5-[4-[4-[4-methyl-5-(3,4,5-trimethoxyphenyl)-3-pyridyl]­phenyl]­piperazine-1-carbonyl]­phenyl]­hexahydropyrimidine-2,4-dione (**17**) (35 mg, 0.0526 mmol, 44%) as a light-colored solid. FTIR (neat): *v*_max_ 2843, 1703, 1617, 1593, 1514, 1454, 1414 cm^–1^. ^1^H NMR (400 MHz, CDCl_3_): δ 8.60 (s, 2H), 7.67 (s, br, 1H), 7.53 (dd, *J* = 8.5, 2.1 Hz, 1H), 7.47 (d, *J* = 2.1 Hz, 1H), 7.31 (d, *J* = 8.9 Hz, 2H), 7.08 (d, *J* = 8.5 Hz, 1H), 7.06 (d, *J* = 8.9 Hz, 2H), 6.55 (s, 2H), 3.95 (s, 6H), 3.92 (s, 6H), 3.89–3.87 (m, 4H), 3.80–3.72 (m, 2H), 3.38–3.36 (m, 4H), 2.86 (t, *J* = 6.7 Hz, 2H), 2.46 (s, 3H) ppm. ^13^C NMR (101 MHz, CDCl_3_): δ 169.2, 169.1, 162.9, 155.8, 153.3, 153.1, 151.7, 150.8, 141.2, 140.9, 139.2, 138.6, 133.3 129.8, 129.4, 128.6, 128.3, 126.8, 125.0, 115.7, 111.6, 105.9, 60.5, 55.9, 55.6, 48.2, 47.9, 44.3, 30.9, 19.2 ppm. HRMS: Calculated for C_37_H_40_N_5_O_7_ [M + H]^+^ 666.2928, found 666.2913. Error <3 ppm. HPLC: Retention time: 5.138 min, peak area: 96.87%.

#### 1-[2-Methoxy-5-[4-[[4-[4-[4-methyl-5-(3,4,5-trimethoxyphenyl)-3-pyridyl]­phenyl]­piperazin-1-yl]­methyl]­piperidine-1-carbonyl]­phenyl]­hexahydropyrimidine-2,4-dione (**18**)

3-(2,4-Dioxohexahydropyrimidin-1-yl)-4-methoxybenzoic acid (30 mg, 0.114 mmol) was stirred with *N,N*-diisopropylethylamine (0.10 mL, 0.574 mmol) and propylphosphonic anhydride solution 50% in EtOAc (0.13 mL, 0.210 mmol) in tetrahydrofuran (0.5 mL) at 50 °C for 30 min, then 1-[4-[4-methyl-5-(3,4,5-trimethoxyphenyl)-3-pyridyl]­phenyl]-4-(4-piperidylmethyl)­piperazine (**38**) (50 mg, 0.0968 mmol) was added. The mixture was stirred at 50 °C for 17 h. Upon cooling, the mixture was diluted with ethyl acetate (10 mL) then washed sequentially with sat. aq. Na_2_CO_3_ (5 mL), 5% HCl (5 mL), and sat. aq. Na_2_CO_3_ (5 mL). The organic layer was passed through a hydrophobic frit and concentrated *in vacuo*. The crude material was purified by reverse phase preparative HPLC eluting with 5–95% MeCN in water + 0.1% v/v TFA to afford 1-[2-methoxy-5-[4-[[4-[4-[4-methyl-5-(3,4,5-trimethoxyphenyl)-3-pyridyl]­phenyl]­piperazin-1-yl]­methyl]­piperidine-1-carbonyl]­phenyl]­hexahydropyrimidine-2,4-dione (**18**) (6.0 mg, 7.90 μmol, 8%) as a light green solid. FTIR (neat): *v*_max_ 3007, 2963, 1695, 1616, 1593, 1518, 1451, 1420 cm^–1^. ^1^H NMR (400 MHz, CDCl_3_): δ 8.61 (s, 1H), 8.58 (s, 1H), 7.77 (s, br, 1H), 7.43 (dd, *J* = 8.4, 2.1 Hz, 1H), 7.39 (d, *J* = 2.1 Hz, 1H), 7.34 (d, *J* = 8.7 Hz, 2H), 7.07–7.02 (m, 3H), 6.54 (s, 2H), 3.95 (s, 3H), 3.93 (s, 3H), 3.91 (s, 6H), 3.90–3.87 (m, 4H), 3.77–3.74 (m, 2H), 3.74–3.72 (m, 2H), 3.65–3.62 (m, 4H), 3.05 (d, *J* = 6.9 Hz, 2H), 2.96–2.93 (m, 2H), 2.84 (t, *J* = 6.7 Hz, 2H), 2.42 (s, 3H), 2.22–2.20 (m, 1H), 1.96 (d, *J* = 11.7 Hz, 2H), 1.39 (d, *J* = 11.7 Hz, 2H) ppm. ^13^C NMR (101 MHz, CDCl_3_): δ 169.7, 168.8, 155.6, 153.2, 152.5, 151.7, 149.4, 144.2, 141.0, 139.8, 139.3, 138.4, 130.0, 129.6, 128.3, 128.2, 127.3, 126.6, 116.3, 111.5, 107.7, 106.0, 62.0, 60.5, 55.9, 55.5, 51.9, 45.5, 44.2, 40.7, 31.3, 30.9, 29.7, 19.0 ppm. HRMS: Calculated for C_43_H_51_N_6_O_7_ [M + H]^+^ 763.3819, found 763.3815. Error <1 ppm. HPLC: Retention time: 5.421 min, peak area: 95.20%.

#### 2-(1-Methyl-2,6-dioxopiperidin-3-yl)-5-(4-((4-(4-(4-methyl-5-(3,4,5-trimethoxyphenyl)­pyridin-3-yl)­phenyl)­piperazin-1-yl)­methyl)­piperidin-1-yl)­isoindoline-1,3-dione (**19**)

1-[4-[4-Methyl-5-(3,4,5-trimethoxyphenyl)-3-pyridyl]­phenyl]-4-(4-piperidylmethyl)­piperazine (**38**) (80 mg, 0.155 mmol) was combined with 5-fluoro-2-(1-methyl-2,6-dioxo-3-piperidyl)­isoindoline-1,3-dione (60 mg, 0.207 mmol) and triethylamine (0.10 mL, 0.717 mmol) in *N,N*-dimethylformamide (0.5 mL) and the mixture was stirred at 100 °C for 17 h. The mixture was concentrated *in vacuo* to remove the solvent, then redissolved in EtOAc (5 mL) and washed with 1 M aq. HCl solution (5 mL). The phases were separated, and the aqueous layer was re-extracted with DCM (3 × 5 mL). The combined organic layers were passed through a hydrophobic frit and concentrated *in vacuo* to afford a yellow solid. Ethyl acetate (10 mL) was added to the crude material and the mixture heated to 100 °C for 10 min. The mixture was then cooled in a freezer for 2 h, then filtered. The crude precipitate obtained was purified by reverse phase preparative HPLC eluting with 5–95% MeCN in water + 0.1% v/v TFA to afford 2-(1-methyl-2,6-dioxo-3-piperidyl)-5-[4-[[4-[4-[4-methyl-5-(3,4,5-trimethoxyphenyl)-3-pyridyl]­phenyl]­piperazin-1-yl]­methyl]-1-piperidyl]­isoindoline-1,3-dione (**19**) (20 mg, 0.0254 mmol, 16%) as a light green solid. ^1^H NMR (500 MHz, CDCl_3_): δ 8.52 (s, 1H), 8.49 (s, 1H), 7.62 (d, *J* = 8.4 Hz, 1H), 7.25 (d, *J* = 8.7 Hz, 2H), 7.20 (d, *J* = 2.4 Hz, 1H), 7.00–6.97 (m, 3H), 6.45 (s, 2H), 4.87 (dd, *J* = 12.4, 5.4 Hz, 1H), 3.89 (d, *J* = 13.3 Hz, 2H), 3.86 (s, 3H), 3.82 (s, 6H), 3.68–3.59 (m, 4H), 3.58–3.48 (m, 4H), 3.14 (s, 3H), 2.95 (d, *J* = 6.4 Hz, 2H), 2.92 (d, *J* = 10.8 Hz, 2H), 2.90–2.89 (m, 1H), 2.75–2.65 (m, 2H), 2.32 (s, 3H), 2.16–2.13 (m, 1H), 2.05–2.01 (m, 1H), 1.97 (d, *J* = 12.2 Hz, 2H), 1.43 (q, *J* = 11.6 Hz, 2H) ppm. ^13^C NMR (126 MHz, CDCl_3_): δ 171.2, 169.0, 168.0, 167.3, 155.1, 153.8, 152.3, 149.9, 147.7, 141.4, 140.7, 139.0, 136.0, 134.5, 130.5, 130.2, 125.4, 120.0, 118.5, 116.9, 110.8, 109.0, 106.6, 62.9, 61.0, 56.4, 52.6, 50.0, 47.6, 46.2, 32.0, 31.6, 29.8, 27.2, 22.1, 19.3 ppm. HRMS: Calculated for C_45_H_51_N_6_O_7_ [M + H]^+^ 787.3819. Found: 787.3809. Error <2 ppm. HPLC: Retention time: 5.048 min, peak area: 95.29%.

#### 2-(2,6-Dioxopiperidin-3-yl)-5-(4-((4-(4-(5-(4-fluoro-3,5-dimethoxyphenyl)-4-methylpyridin-3-yl)­phenyl)­piperazin-1-yl)­methyl)­piperidin-1-yl)­isoindoline-1,3-dione (**20**)

2-(2,6-Dioxo-3-piperidyl)-5-fluoroisoindoline-1,3-dione (110 mg, 0.398 mmol) was combined with 1-[4-[5-(4-fluoro-3,5-dimethoxyphenyl)-4-methyl-3-pyridyl]­phenyl]-4-(4-piperidylmethyl)­piperazine (**42**) (150 mg, 0.297 mmol) and triethylamine (0.23 mL, 1.62 mmol) in *N,N*-dimethylformamide (1 mL) and the mixture was stirred at 100 °C for 17 h. The mixture was concentrated *in vacuo* to remove the solvent, then redissolved in DCM (10 mL) and washed with 1 M aq. HCl solution (10 mL). The organic layer was passed through a hydrophobic frit and concentrated *in vacuo*. The crude material was purified by reverse phase preparative HPLC eluting with 20–80% MeCN in water + 0.1% v/v formic acid to afford 2-(2,6-dioxopiperidin-3-yl)-5-(4-((4-(4-(5-(4-fluoro-3,5-dimethoxyphenyl)-4-methylpyridin-3-yl)­phenyl)­piperazin-1-yl)­methyl)­piperidin-1-yl)­isoindoline-1,3-dione (**20**) (25 mg, 0.033 mmol, 11%) as a yellow solid. ^1^H NMR (400 MHz, CDCl_3_): δ 8.43 (s, 1H), 8.38 (s, 1H), 8.32 (s, 1H), 7.68 (d, *J* = 8.5 Hz, 1H), 7.30 (d, *J* = 2.4 Hz, 1H), 7.28 (d, *J* = 8.9 Hz, 2H), 7.06 (dd, *J* = 8.5, 2.4 Hz, 1H), 7.01 (d, *J* = 8.9 Hz, 2H), 6.58 (d, *J* = 6.9 Hz, 2H), 4.94 (dd, *J* = 12.5, 5.3 Hz, 1H), 3.98 (d, *J* = 12.5 Hz, 2H), 3.91 (s, 6H), 3.32 (t, *J* = 5.2 Hz, 4H), 2.99 (t, *J* = 11.4 Hz, 2H), 2.93–2.81 (m, 2H), 2.81–2.71 (m, 2H), 2.69 (t, *J* = 5.2 Hz, 4H), 2.36 (d, *J* = 6.8 Hz, 2H), 2.19 (s, 3H), 2.16–2.09 (m, 1H), 1.95 (d, *J* = 12.5 Hz, 2H), 1.34 (q, *J* = 10.1 Hz, 2H) ppm. ^13^C NMR (101 MHz, CDCl_3_): δ 171.1, 168.4, 168.1, 167.3, 155.4, 150.5, 148.9, 148.3 (d, ^3^ *J*_C–F_ = 8.1 Hz), 147.8, 143.3 (d, ^1^ *J*_C–F_ = 246.4 Hz), 142.6, 137.8 (d, ^2^ *J*_C–F_ = 18 Hz), 134.4, 133.6 (d, ^4^ *J*_C–F_ = 5.1 Hz), 133.6, 130.3, 128.8, 125.5, 118.6, 117.8, 115.6, 108.6, 107.2, 64.1, 56.7, 53.5, 49.1, 48.5, 48.0, 33.1, 31.5, 30.1, 22.8, 18.1 ppm. ^19^F­{^1^H} NMR (376 MHz, CDCl_3_): δ −159.5 ppm. HRMS: Calculated for C_43_H_46_FN_6_O_6_ [M + H]^+^ 761.3463, found 761.3462. Error <1 ppm. UPLC-MS: Retention time: 3.22 min, peak area: 100%.

#### 2-(2,6-Dioxopiperidin-3-yl)-5-(4-(4-(4-(5-(4-fluoro-3,5-dimethoxyphenyl)-4-methylpyridin-3-yl)­phenyl)­piperazine-1-carbonyl)­piperidin-1-yl)­isoindoline-1,3-dione (**21**)

2-(2,6-Dioxo-3-piperidyl)-5-fluoroisoindoline-1,3-dione (45 mg, 0.163 mmol) was combined with [4-[4-[5-(4-fluoro-3,5-dimethoxyphenyl)-4-methyl-3-pyridyl]­phenyl]­piperazin-1-yl]-(4-piperidyl)­methanone (**43**) (70 mg, 0.135 mmol) and triethylamine (0.10 mL, 0.717 mmol) in *N,N*-dimethylformamide (0.5 mL) and the mixture was stirred at 100 °C for 17 h. The mixture was concentrated *in vacuo* to remove the solvent, then redissolved in THF (10 mL) and washed with 1 M aq. HCl solution (10 mL). The THF layer was discarded, and the aqueous layer was extracted with DCM (3 × 5 mL). The combined organic layers were passed through a hydrophobic frit and concentrated *in vacuo*. The crude material was purified by reverse phase preparative HPLC eluting with 5–95% MeCN in water + 0.1% v/v TFA to afford 2-(2,6-dioxo-3-piperidyl)-5-[4-[4-[4-[5-(4-fluoro-3,5-dimethoxyphenyl)-3-pyridyl]­phenyl]­piperazine-1-carbonyl]-1-piperidyl]­isoindoline-1,3-dione (**21**) (12 mg, 0.0150 mmol, 11%) as a light green solid. ^1^H NMR (500 MHz, CDCl_3_): δ 8.63 (s, 1H), 8.60 (s, 1H), 8.15 (s, 1H), 7.72 (d, *J* = 8.4 Hz, 1H), 7.33 (d, *J* = 8.7 Hz, 2H), 7.31 (d, *J* = 2.6 Hz, 1H), 7.10 (dd, *J* = 8.4, 2.6 Hz, 1H), 7.08 (d, *J* = 8.7 Hz, 2H), 6.60 (d, *J* = 6.6 Hz, 2H), 4.99–4.96 (m, 1H), 4.02 (d, *J* = 13.0 Hz, 2H), 3.95 (s, 6H), 3.89–3.87 (m, 2H), 3.80–3.79 (m, 2H), 3.40–3.38 (m, 2H), 3.35–3.31 (m, 2H), 3.11 (t, *J* = 10.8 Hz, 2H), 2.95–2.85 (m, 2H), 2.84–2.71 (m, 2H), 2.45 (s, 3H), 2.20–2.09 (m, 1H), 2.02 (q, *J* = 11.1 Hz, 2H), 1.92 (d, *J* = 11.1 Hz, 2H) ppm. ^13^C NMR (126 MHz, CDCl_3_): δ 172.9, 171.1, 168.3, 167.9, 167.2, 161.1, 155.3, 153.8, 151.2, 149.0 (d, ^3^ *J*_C–F_ = 7.6 Hz), 143.0 (d, ^1^ *J*_C–F_ = 250.1 Hz), 141.5 (d, ^2^ *J*_C–F_ = 40.3 Hz), 140.0, 139.1, 134.4, 130.3, 129.7 (d, ^4^ *J*_C–F_ = 6.3 Hz), 125.5, 125.4, 119.3, 118.2, 116.2, 108.8, 107.1, 56.9, 49.2, 47.4, 46.2, 37.9, 31.4, 28.0, 27.8, 22.8, 19.6 ppm. ^19^F NMR (471 MHz, CDCl_3_): δ −159.5 (t, *J* = 9.4 Hz) ppm. HRMS: Calculated for C_43_H_44_FN_6_O_7_ [M + H]^+^ 775.3256. Found: 775.3222. Error <5 ppm. HPLC: Retention time: 6.263 min, peak area: 95.68%.

#### 2-(2,6-Dioxopiperidin-3-yl)-5-(3-((4-(4-(5-(4-fluoro-3,5-dimethoxyphenyl)-4-methylpyridin-3-yl)­phenyl)­piperazin-1-yl)­methyl)­pyrrolidin-1-yl)­isoindoline-1,3-dione (**22**)

2-(2,6-Dioxo-3-piperidyl)-5-fluoroisoindoline-1,3-dione (33 mg, 0.121 mmol) was combined with 1-[4-[5-(4-fluoro-3,5-dimethoxyphenyl)-4-methyl-3-pyridyl]­phenyl]-4-(pyrrolidin-3-ylmethyl)­piperazine (**44**) (50 mg, 0.102 mmol) and triethylamine (0.077 mL, 0.553 mmol) in *N,N*-dimethylformamide (1 mL) and the mixture was stirred at 100 °C for 17 h. The mixture was concentrated *in vacuo* to remove the solvent, then redissolved in DCM (10 mL) and washed with 1 M aq. HCl solution (10 mL). The organic layer was passed through a hydrophobic frit and concentrated *in vacuo* to afford a green oil. The crude material was purified by reverse phase preparative HPLC eluting with 40–100% MeOH in water + 0.1% v/v formic acid to afford 2-(2,6-dioxo-3-piperidyl)-5-[3-[[4-[4-[5-(4-fluoro-3,5-dimethoxyphenyl)-4-methyl-3-pyridyl]­phenyl]­piperazin-1-yl]­methyl]­pyrrolidin-1-yl]­isoindoline-1,3-dione (**22**) (16 mg, 0.0214 mmol, 21%) as a light yellow solid. ^1^H NMR (400 MHz, CDCl_3_): δ 8.43 (s, 1H), 8.38 (s, 1H), 8.16 (s, 1H), 7.66 (d, *J* = 8.5 Hz, 1H), 7.27 (d, *J* = 8.9 Hz, 2H), 7.02 (d, *J* = 8.9 Hz, 2H), 6.97 (d, *J* = 2.3 Hz, 1H), 6.70 (dd, *J* = 8.5, 2.3 Hz, 1H), 6.58 (d, *J* = 7.0 Hz, 2H), 4.94 (dd, *J* = 12.4, 5.3 Hz, 1H), 3.91 (s, 6H), 3.61 (dd, *J* = 10.1, 7.3 Hz, 1H), 3.52 (td, *J* = 9.1, 4.3 Hz, 1H), 3.48–3.40 (m, 1H), 3.30 (t, *J* = 5.1 Hz, 4H), 3.24 (dd, *J* = 10.1, 7.3 Hz, 1H), 2.94–2.79 (m, 2H), 2.79–2.66 (m, 4H), 2.64 (dd, *J* = 10.5, 5.4 Hz, 2H), 2.55–2.43 (m, 2H), 2.25 (dt, *J* = 11.4, 5.4 Hz, 1H), 2.19 (s, 3H), 2.16–2.09 (m, 1H), 1.91–1.83 (m, 1H) ppm. ^13^C NMR (101 MHz, CDCl_3_): δ 171.1, 168.4, 168.3, 167.6, 157.3, 152.1, 150.6, 149.3, 148.2 (d, ^3^ *J*_C–F_ = 8.1 Hz), 148.1, 143.3 (d, ^1^ *J*_C–F_ = 246.4 Hz), 142.3, 137.7 (d, ^2^ *J*_C–F_ = 20.2 Hz), 134.5, 133.8 (d, ^4^ *J*_C–F_ = 5.1 Hz), 130.3, 128.9, 125.5, 116.6, 115.5, 115.1, 107.3, 106.2, 61.6, 56.7, 53.6, 52.7, 49.1, 48.7, 47.5, 36.3, 31.5, 29.8, 22.8, 18.0 ppm. ^19^F­{^1^H} NMR (376 MHz, CDCl_3_): δ −159.6 ppm. HRMS: Calculated for C_42_H_44_FN_6_O_6_ [M + H]^+^ 747.3306, found 747.3301. Error <1 ppm. UPLC-MS: Retention time: 3.23 min, peak area: 100.00%.

#### 2-(2,6-Dioxopiperidin-3-yl)-5-(3-(4-(4-(5-(4-fluoro-3,5-dimethoxyphenyl)-4-methylpyridin-3-yl)­phenyl)­piperazine-1-carbonyl)­pyrrolidin-1-yl)­isoindoline-1,3-dione (**23**)

2-(2,6-Dioxo-3-piperidyl)-5-fluoroisoindoline-1,3-dione (65 mg, 0.235 mmol) was combined with [4-[4-[5-(4-fluoro-3,5-dimethoxyphenyl)-4-methyl-3-pyridyl]­phenyl]­piperazin-1-yl]­pyrrolidin-3-ylmethanone (**45**) (100 mg, 0.198 mmol) and triethylamine (0.15 mL, 1.08 mmol) in *N,N*-dimethylformamide (1 mL) and the mixture was stirred at 100 °C for 17 h. The mixture was concentrated *in vacuo* to remove the solvent, then redissolved in DCM (10 mL) and washed with 1 M aq. HCl solution (10 mL). The organic layer was passed through a hydrophobic frit and concentrated *in vacuo* to afford a green oil. The crude material was purified by reverse phase preparative HPLC eluting with 40–100% MeOH in water + 0.1% v/v formic acid to afford 2-(2,6-dioxo-3-piperidyl)-5-[3-[4-[4-[5-(4-fluoro-3,5-dimethoxyphenyl)-4-methyl-3-pyridyl]­phenyl]­piperazine-1-carbonyl]­pyrrolidin-1-yl]­isoindoline-1,3-dione (**23**) (30 mg, 0.0394 mmol, 20%) as a light yellow solid. ^1^H NMR (400 MHz, CDCl_3_): δ 8.43 (s, 1H), 8.40 (s, 1H), 7.99 (s, 1H), 7.68 (d, *J* = 8.4 Hz, 1H), 7.31 (d, *J* = 8.8 Hz, 2H), 7.04 (d, *J* = 8.8 Hz, 2H), 6.97 (d, *J* = 2.2 Hz, 1H), 6.72 (dd, *J* = 8.4, 2.2 Hz, 1H), 6.58 (d, *J* = 7.0 Hz, 2H), 4.94 (dd, *J* = 12.3, 5.3 Hz, 1H), 3.92 (s, 6H), 3.87 (t, *J* = 5.0 Hz, 2H), 3.78 (t, *J* = 5.0 Hz, 2H), 3.74 (d, *J* = 6.8 Hz, 1H), 3.69 (d, *J* = 7.7 Hz, 1H), 3.65–3.61 (m, 1H), 3.55–3.49 (m, 2H), 3.35–3.32 (m, 2H), 3.29 (t, *J* = 5.0 Hz, 2H), 2.94–2.82 (m, 2H), 2.84–2.71 (m, 2H), 2.45–2.40 (m, 1H), 2.37–2.31 (m, 1H), 2.18 (s, 3H) ppm.^13^C NMR (101 MHz, CDCl_3_): δ 171.0, 170.4, 168.3, 168.1, 167.5, 151.7, 150.1, 149.2, 148.3 (d, ^3^ *J*_C–F_ = 9.1 Hz), 143.3 (d, ^4^ *J*_C–F_ = 246.0 Hz), 142.3, 137.6 (d, ^2^ *J*_C–F_ = 19.2 Hz), 134.5, 133.6 (d, ^2^ *J*_C–F_ = 6.0 Hz), 130.5, 130.0, 125.5, 121.2, 118.8, 117.2, 116.3, 115.4, 107.3, 106.3, 56.7, 50.9, 49.6, 49.1, 47.8, 45.6, 40.2, 31.5, 29.0, 22.8, 18.0 ppm. ^19^F­{^1^H} NMR (376 MHz, CDCl_3_): δ −159.5 ppm. HRMS: Calculated for C_42_H_42_FN_6_O_7_ [M + H]^+^ 761.3099, found 761.3102. Error <1 ppm. UPLC-MS: Retention time: 3.298 min, peak area: 100.00%.

#### 1-(5-(4-(4-(5-(4-Fluoro-3,5-dimethoxyphenyl)-4-methylpyridin-3-yl)­phenyl)­piperazine-1-carbonyl)-2-methoxyphenyl)­dihydropyrimidine-2,4­(1*H*,3*H*)-dione (**24**)

3-(2,4-Dioxohexahydropyrimidin-1-yl)-4-methoxybenzoic acid (35 mg, 0.132 mmol) was stirred with *N,N*-diisopropylethylamine (0.10 mL, 0.574 mmol) and propylphosphonic anhydride solution 50% in EtOAc (50%, 0.15 mL, 0.252 mmol) in *N,N*-dimethylformamide (0.5 mL) at 50 °C for 30 min, then 1-[4-[5-(4-fluoro-3,5-dimethoxyphenyl)-4-methyl-3-pyridyl]­phenyl]­piperazine (**7**) (50 mg, 0.123 mmol) was added. The mixture was stirred at 50 °C for 17 h. The mixture was concentrated *in vacuo* to remove the solvent, then redissolved in ethyl acetate (5 mL) and washed with sat. aq. Na_2_CO_3_ (5 mL). The organic phase was passed through a hydrophobic frit and concentrated *in vacuo*. The crude material was purified by reverse phase preparative HPLC eluting with 5–95% MeCN in water + 0.1% v/v TFA to afford 1-[5-[4-[4-[5-(4-fluoro-3,5-dimethoxyphenyl)-4-methyl-3-pyridyl]­phenyl]­piperazine-1-carbonyl]-2-methoxyphenyl]­hexahydropyrimidine-2,4-dione (**24**) (17 mg, 0.0260 mmol, 21%) as a white solid. ^1^H NMR (500 MHz, CDCl_3_): δ 8.59 (s, 1H), 8.57 (s, 1H), 7.91 (s, 1H), 7.52 (dd, *J* = 8.5, 2.3 Hz, 1H), 7.47 (d, *J* = 2.3 Hz, 1H), 7.30 (d, *J* = 8.7 Hz, 2H), 7.08–7.06 (m, 3H), 6.59 (d, *J* = 6.6 Hz, 2H), 3.94 (s, 9H), 3.91–3.89 (m, 4H), 3.76–3.74 (m, 2H), 3.36–3.34 (m, 4H), 2.85 (t, *J* = 6.6 Hz, 2H), 2.42 (s, 3H) ppm. ^13^C NMR (126 MHz, CDCl_3_): δ 169.9, 169.5, 156.3, 153.2, 152.3, 151.3, 148.9 (d, ^3^ *J*_C–F_ = 8.8 Hz), 143.0 (d, ^1^ *J*_C–F_ = 249.5 Hz), 141.4 (d, ^2^ *J*_C–F_ = 50.4 Hz), 140.3, 139.3, 130.3, 130.0 (d, ^4^ *J*_C–F_ = 5.0 Hz), 129.2, 129.1, 128.7, 127.5, 127.4, 125.4, 116.2, 112.1, 107.1, 56.9, 56.8, 56.1, 48.7, 44.7, 31.4, 19.5 ppm. ^19^F NMR (471 MHz, CDCl_3_): δ −159.5 (t, *J* = 4.7 Hz) ppm. HRMS: Calculated for C_36_H_37_FN_5_O_6_ [M + H]^+^ 654.2728. Found: 654.2701, error <5 ppm. HPLC: Retention time: 5.339 min, peak area: 95.63%.

### HEK-293 and U87-MG Cell Culture and Transfection

Culturing and passaging of adherent cell lines were carried out using an aseptic technique in biological safety cabinets. All cells were incubated in a 37 °C incubator with 5% CO_2_. Cell lines were regularly tested for mycoplasma contamination. Cells were seeded in PDL-coated plates, and maintained in Dulbecco’s modified Eagle medium (DMEM, Gibco) supplemented with 10% fetal bovine serum (FBS) (Thermo Fisher). HEK-293 cells were transfected with the protein expression or reporter constructs (ACVR1-HiBiT, VectorBuilder: VB210721–1110mun) using FuGENE HD (Promega: E2311) according to the manufacturer’s instructions. Briefly, DNA was diluted into phenol red-free Opti-MEM (Gibco) at a concentration of 10 μg/mL. Without coming in contact with the sides of the container, 3 μL of FuGENE HD was added for each microgram of DNA used. After thorough mixing by inversion, FuGENE HD/DNA complexes were allowed to form by incubation at room temperature for 20 min. The transfection mixture (1 part) was added to 20 parts of the HEK-293 cell suspension and the HEK-293 cells were incubated in a humidified, 37 °C incubator with 5% CO_2_ for 24 h. All cells were treated with the indicated compounds such that the final concentration of DMSO was 0.1%.

### Patient-Derived PDHGG Cell Culture

Cells were grown in stem cell media consisting of 250 mL DMEM/F12 (Thermo Fisher Scientific, 11330-038), 250 mL Neurobasal-A Medium (Thermo Fisher Scientific, 10888-022), 10 mM HEPES Buffer Solution (Thermo Fisher Scientific, 15630-080), 1 mM MEM Sodium Pyruvate Solution (Thermo Fisher Scientific, 11360-070), 0.1 mM MEM Non-Essential Amino Acids Solution (Thermo Fisher Scientific, 11140-050) and 1× Glutamax-I Supplement (Thermo Fisher Scientific, 35050-061). The media was supplemented with B-27 Supplement Minus Vitamin A 1:50 (Thermo Fisher Scientific, 12587-010), 20 ng/mL recombinant Human-EGF (2B Scientific LTD, Oxford, UK, 100-26), 20 ng/mL recombinant Human-FGF (2B Scientific LTD, 100-146), 10 ng/mL recombinant Human-PDGF-AA (2B Scientific LTD, 100-16), 10 ng/mL recombinant Human-PDGF-BB (2B Scientific LTD, 100-18), and 2 μg/mL Heparin Solution (Stem Cell Technologies, Cambridge, UK, 07980). For 2D cultures, cell culture flasks and plates were precoated with 2 μg/mL Cultrex laminin I (Bio-techne, 3446-005-01) for >2 h at 37 °C prior to seeding. All cells were incubated in a humidified 37 °C incubator with 5% CO_2_. Cell lines were regularly STR profiled and tested for mycoplasma contamination.

### Concentration–Response Curves and GI_50_ Determinations

Cells were seeded at optimum seeding densities in 384-well plates in 40 μL media and incubated for 72 h at 37 °C. Cells were then treated with a range of drug concentrations (0.0002–30 μM) using the automated ECHO acoustic dispenser (Beckman Coulter, UK) and incubated for 192 h at 37 °C. Cell viability was determined using CellTiter-Glo reagent (Promega, G7573) and the percentage cell viability normalized to DMSO vehicle control was calculated. GI_50_ values were determined in Prism using the [Inhibitor] vs response — Variable slope (four parameters) curve fitting (*n* ≥ 2).

### Western Blotting

Following the treatment of cells with compounds at indicated concentrations and time periods, the media was removed, and the cells were washed with PBS and lysed in RIPA buffer, supplemented with complete EDTA-free protease inhibitor cocktail (Roche) and benzonase nuclease (Sigma-Aldrich). The cells were then placed on ice and scraped into Eppendorf tubes. Cell lysates were centrifuged at 15,000*g* at 4 °C for 15 min, and the supernatant/soluble fraction was collected. Cell lysates were diluted with 4x sample buffer and heated at 95 °C for 5 min. Equal amounts of protein were then loaded onto precast Bolt Bis-Tris Plus 4–12% Mini Protein Gels (Thermo Fisher) and resolved at 225 V for 30 min using 1× Bolt MOPS-SDS Running Buffer (Thermo Fisher). Proteins were electrophoretically transferred onto a nitrocellulose membrane at 10 V for 60 min in 1× Bolt Transfer Buffer. The transferred membrane was blocked with 5% (w/v) skim milk powder dissolved in TBS-Tween for 45 min at room temperature, then washed three times with TBS-Tween and incubated in primary antibodies (Anti-HiBiT mouse monoclonal antibody, Promega: N7200, 1:1000 dilution; Anti-GAPDH rabbit monoclonal antibody, Thermo Fisher: 4A9L6, 1:3000 dilution) overnight at 4 °C. The membrane was washed three times with TBS-Tween then incubated with secondary antibodies (IRDye 800CW donkey antimouse IgG secondary antibody, LI-COR: 926-32212, 1:3000 dilution; IRDye 680CW donkey antirabbit IgG secondary antibody, LI-COR: 926-68073, 1:3000 dilution) for 1 h at room temperature. The membrane was then washed three times with TBS-Tween and was imaged, and bands representing the target proteins were quantified using an Odyssey Li-Cor fluorescent scanner. Protein expression was normalized by comparing with the loading controls (GAPDH) and data was plotted in GraphPad Prism 9. The apparent DC_50_ values of test compounds were estimated using the [Inhibitor] versus response (four-parameter) nonlinear regression curve fitting function of GraphPad Prism 9.

### NanoBRET Target Engagement Assay

20,000 HEK-293 cells were seeded in 96-well plates in Dulbecco’s modified Eagle medium (DMEM, Gibco). The cells were transfected with ACVR1-NL or TGFBR1-NL plasmids using Fugene HD and Promega Carrier DNA and incubated overnight. The media was replaced with OPTIMEM (phenol-free) containing serial diluted compounds starting at 10 μM, and 0.078 μM Tracer K11 for ACVR1-NL plate or 0.5 μM Tracer K14 for TGFBR1-NL plate, which were then incubated for 2 h, followed by addition of 50 μL Nano-Glo substrate mixture to each well. Luminescence was measured at 460 and 610 nm on a Varioskan plate reader (ThermoScientific) and BRET ratio was calculated and normalized response was plotted using GraphPad Prism 9. Three biologically independent replicates were conducted for each sample. The apparent IC_50_ values of test compounds were estimated using the [Inhibitor] versus response (four-parameter) nonlinear regression curve fitting function of GraphPad Prism 9.

### NanoBRET Protein–Protein Interaction (PPI) Assay

HEK-293 cells were maintained in DMEM supplemented with 10% FBS at 37 °C and 5% CO_2_ and seeded at 4.4 × 10^5^ cells/mL. After a 4 h attachment period, cells were transiently cotransfected with C-terminal ALK2–NanoLuc (pFC32K, Promega) and N-terminal HaloTag–CRBN (pFC27K, Promega) expression constructs at a 1:100 donor:acceptor plasmid ratio. Transfections were performed using FuGENE at a 1:3 DNA:reagent ratio, and cells were incubated for 20 h prior to assay. Compounds were serially diluted in DMSO to generate 100× stock solutions and subsequently diluted into Opti-MEM (phenol-free) containing MG132 to prepare 10× intermediate working solutions. Intermediates were predispensed into 384-well white plates and, upon addition of cells, yielded a final 3-fold dilution series with a top concentration sufficient to define the dose–response curve. MG132 was maintained at a final concentration of 10 μM and the DMSO concentration at approximately 1%. Vehicle and HaloTag-ligand-free control wells were included. Following transfection, cells were harvested, resuspended in Opti-MEM at 2.2 × 10^5^ cells/mL, and labeled with HaloTag 618 ligand (Promega; final concentration 1 μM) or DMSO control prior to dispensing onto the compound-containing plate. Cells were incubated with compounds for 4 h. Immediately prior to reading, 10 μL NanoBRET NanoGlo substrate (1:100 dilution in Opti-MEM) was added to each well. Luminescence was measured at 460 nm (donor) and 610 nm (acceptor) using a PHERAstar FSX plate reader (BMG Labtech). BRET ratios were calculated as milli-BRET units (mBU) with background subtraction using the mean mBU of HaloTag-ligand-free controls. Technical triplicates were averaged, and background-subtracted mBU values were plotted against compound concentration and fitted using a four-parameter logistic model (log­[agonist] vs response, variable slope) in GraphPad Prism 9 to derive EC_50_ values. The pEC_50_ was calculated as −log_10_(EC_50_ in M).

### Quantitative Proteomics

Following the treatment of cells with compounds at indicated concentrations and time periods, the media was removed, and the cells were washed with PBS and lysed in RIPA buffer, supplemented with complete EDTA-free protease inhibitor cocktail and Benzonase nuclease. Cell lysates were centrifuged at 15,000*g* at 4 °C for 15 min and the supernatant collected. The protein concentration was measured using BCA protein assay. To 20 μg of protein sample 20% SDS was added to make up 5% SDS final concentration. Protein digestion was performed using an S-Trap micro column (Protifi) according to the manufacturer’s protocol. Briefly, 20 μg protein in lysis buffer was reduced and alkylated using 10 mM dithiothreitol at 37 °C for 20 min, and 20 mM iodoacetamide at r.t. for 30 min. 1/10 volume of 27.5% phosphoric acid was added to the sample, which was vortexed, and subsequently 6-fold volume of binding buffer (90%, v/v, MeOH in 100 mM TEAB) was added to the protein solution which was vortexed. The solution was loaded into an S-Trap micro column. The solution was removed by spinning the column at 4,000 *g* for 30 s. The column was washed with 150 μL binding buffer three times. Finally, 25 μL of digestion solution (1 μg trypsin in 50 mM TEAB per sample) was added to the column and incubated at 37 °C overnight. The digested peptide was eluted using 40 μL of the following buffers consecutively: (1) 50 mM TEAB, (2) 0.2% (v/v) formic acid (FA) in H_2_O, and (3) 50% (v/v) acetonitrile. Elution solutions were collected in a tube and dried under vacuum. The peptides were reconstituted in 0.1% formic acid prior to loading on a mass spectrometer. The peptide samples were analyzed in an *Orbitrap Exploris* 240 (Thermo Fisher Scientific) mass spectrometer coupled to a Vanquish Neo UHPLC liquid chromatography system (Thermo Fisher Scientific). The peptides were loaded on a precolumn PepMap Neo, C_18_, 5 μm, 300 μm × 5 mm, Trap 3PK 1500 bar, Thermo Fisher Scientific) and LC separation was carried out on a commercial 50 cm PepMap C_18_ column (PepMap Neo, 1500 bar, 75 μm × 500 mm, C_18_, 2 μm, 100 Å, Thermo Fisher Scientific) at a flow rate of 250 nL/min with buffer A (0.1% (v/v) formic acid in water) and buffer B (0.1% (v/v) FA in 80% acetonitrile) using the following gradient: 5–25% buffer B for 55 min, 25–40% buffer B for 15 min, 40–99% buffer B for 1 min, 99% buffer B for 10 min. The full MS resolution was set to 120,000 with a scan range of 375–1500 *m*/*z*. The normalized automatic gain control target was 300%, and the maximum injection time for the full scans was set to 50 ms. For DIA scans 75 windows at 8m/z were acquired at resolution 30,000. Precursor mass range was 400–1000 with HCD collision energy 30%. Automatic gain control target value for fragment spectra was set to 1000%. The raw files were analyzed in DIA-NN v1.8 in library-free mode. Precursor ion generation settings were as follows: Carbamidomethyl on Cysteine was set as a fixed modification and Methionine oxidation, *N*-terminal acetylation, and *N*-terminal Methionine excision were set as variable modifications; Trypsin/P with maximum 1 missed cleavage; peptide length from 7 to 30. The differential expression analyses were performed in R (v. 4.0.3) and the global p-values and fold changes were calculated via the Bioconductor package Limma7 (v 3.46.0).

### Kinetic Solubility Assay

The aqueous buffer used was fed state simulated intestinal fluid (FeSSIF, from FFF powder and FeSSIF buffer concentrate, Biorelevant, London, UK), adjusted to pH 5.0. Using a 10 mM stock solution of each test and control compound (hydrocortisone, imipramine, and nicardipine) in 100% DMSO, dilutions were prepared to a theoretical concentration of 200 μM in both FeSSIF, to a 2% final DMSO content, and in 100% DMSO. All dilutions (*n* = 2, in 96-well plates) were allowed to equilibrate at RT on an orbital shaker for 2 h. The aqueous buffer dilutions were filtered using a MultiScreen HTS solubility filter plate (Millipore), and the filtrate was analyzed by LC-UV with confirmation of the peak of interest by mass spectrometry. The concentration of the compound in filtrate was determined by comparing the UV absorbance peak with that of the 100% DMSO dilutions as single point calibration standards.

### Liver Microsomal Stability Assay

Incubations of test and control compounds (chlorpromazine, dextromethorphan, and midazolam) were prepared at 1 μM, *n* = 2, incubated at 37 °C in pooled liver microsomes (Xenotech H0610 or M1000, 0.5 mg protein/mL in 0.1 M phosphate buffer pH 7.4) and were initiated with the addition of NADPH (1 mM). Samples (25 μL) were obtained at 0, 5, 10, 20, and 40 min and added to 75 μL of acetonitrile containing tolbutamide as the analytical internal standard (IS), centrifuged, and the supernatant was removed and analyzed by LC MS/MS (optimized methods for each analyte). The percentage parent remaining at each time point was calculated from the IS normalized peak heights:

$$\begin{array}{l} Parent\;Remaining\;\left(\%\right)=100\times \frac{Compound\;Peak\;Area\;at\;selected\;Timepoint}{Compound\;Peak\;Area\;at\;Time\;0}\\ Parent\;Turnover\;\left(\%\right)=100-Parent\;Remaining\;\left(\%\right) \end{array}$$

Half-life was calculated as

$$\mathrm{Half}‐\mathrm{life}\;\left(\min\right)=\frac{-0.693}{\lambda }$$

Where λ was the slope of the linear portion of Ln percentage remaining vs time. And intrinsic clearance was calculated using

$$Intrinsic\;Clearance\;\left(\mu L/\min/mg\right)=\frac{0.693}{Mean\;Half-life\;\left(\min\right)}\times \frac{Incubation\;Volume\;\left(\mu L\right)}{mg\;microsomes\;\mathrm{in}\;incubation}$$

### U87-MG CellTiter-Glo Assay

U87-MG cells were plated at a density of 2000 cells/well using a Thermo matrix multichannel pipet (Thermo Scientific), in 25 μL of cell culture medium in a 384-well microplate (white for CTG, revity#6007680), in all wells excluding columns 1, 2, 23, and 24, and rows A, B, O, and P where 25 μL of cell media only was added (cells excluded to mitigate edge effect). Plates were then incubated overnight at 37 °C and 5% CO_2_. Compound plates were prepared on the D300e Digital Dispenser (Tecan), containing compound response curves with final concentration of 30 μM top in a 1/2 Log dilution series, with a final DMSO concentration of 0.5%. Compound plates were diluted with 40 μL of cell media. Using a Thermo matrix multichannel pipet (Thermo Scientific), 25 μL of each compound was added to each relevant column of the cell plate. The assay plates were incubated for 96 h before adding 30 μL of CTG reagent (Promega), which produces light in direct proportion to the amount of adenosine triphosphate and viable cells present. The plates were shaken at room temperature for 20 min. The luminescence was measured in relative light units using a Pherastar Microplate reader (BMG Labtech).

### MOLM13 CellTiter-Glo Assay

Compound plates were prepared using the D300e Digital Dispenser (Tecan), generating compound response curves with a final top concentration of 30 μM and 10-point, 1:3 serial dilutions. The final DMSO concentration was maintained at 0.5% across all wells. MOLM-13 cells were seeded at a density of 1,000 cells per well in 40 μL of cell culture medium in 384-well plates (Greiner 781090). Media alone was added to blank control wells. Plates were gently agitated on a shaker for 2–5 min, before incubating at 37 °C with 5% CO_2_ for 4 days. Following incubation, 30 μL of CellTiter-Glo 2.0 reagent (Promega) was added to each well, and plates were shaken at room temperature for 10–15 min. Luminescence was then measured in relative light units (RLU) using a PHERAstar plate reader (BMG Labtech).

### MV4–11, U-937, and SK-OV-3 CellTiter-Glo Assays

These assays were performed at Reaction Biology (Freiburg, Germany). Compound plates were prepared using the D300e Digital Dispenser (Tecan), generating compound response curves with a final top concentration of 30 μM and 8-point serial dilutions. The final DMSO concentration was maintained at 0.1% across all wells. Cells were seeded in white cell culture-treated flat and clear bottom 384-well plates and incubated at 37 °C overnight before compounds were added. After incubation for 72 h at 37 °C at 5% CO_2_, cell plates were equilibrated to room temperature for 1 h, CellTiter-Glo reagent (Promega) was added, and luminescence was measured approximately 1 h later using a luminometer (EnVision, PerkinElmer). Raw data were converted into percent cell viability relative to the high (DMSO) and low (10 μM staurosporine) controls, which were set to 100% and 0%, respectively. Absolute IC_50_ values were calculated by GraphPad Prism software with a variable slope sigmoidal response fitting model using 0% viability as bottom constraint and 100% viability as top constraint.

## Supplementary Material





## Data Availability

All proteomics data is available to access through the PRoteomics IDEntifications Database (PRIDE).

## Notes

^1^H, ^13^C, and ^19^F NMR, HPLC, UPLC-MS, and HRMS spectra of key intermediates and final compounds, additional figures, immunoblot quantification, and statistical analysis (PDF)
Molecular formula strings of final compounds (CSV)

## Abbreviations Used

ABL1: ABL proto-oncogene 1
ACVR1: activin A receptor type I
ALK2: activin receptor-like kinase 2
ALK5: activin receptor-like kinase 5
BMP: bone morphogenetic protein
CCR2: CC chemokine receptor 2
ChromLogD: chromatographic logD
CK1α: casein kinase 1 alpha
CPME: cyclopentyl methyl ether
CRBN: cereblon
CTG: CellTiter-Glo
DCM: dichloromethane
DIPEA: *N,N*-diisopropylethylamine
DIPG: diffuse intrinsic pontine glioma
DMF: *N,N*-dimethylformamide
DMP: Dess-Martin periodinane
DMSO: dimethyl sulfoxide
EGFR: epidermal growth factor receptor
EPSA: exposed polar surface area
Et_3_N: triethylamine
EtOH: ethanol
EtOAc: ethyl acetate
FESSIF: fed-state simulated intestinal fluid
FIZ1: FLT3-interacting zinc finger 1
FOP: fibrodysplasia ossificans progressiva
GPCR: G-protein coupled receptor
GS: glycine-serine
GSPT1: G1 To S Phase Transition 1
HATU: hexafluorophosphate azabenzotriazole tetramethyl uronium
HO: heterotopic ossification
HLM: human liver microsomes
ID1: inhibitor of DNA binding 1
JAK: janus kinase
KO*t*Bu: potassium *tert*-butoxide
MeCN: acetonitrile
MeOH: methanol
MINK: misshapen like kinase 1
MLM: mouse liver microsomes
MOA: mechanism of action
MsCl: methanesulfonyl chloride
PDE6D: phosphodiesterase 6 subunit delta
PDHGG: pediatric diffuse high-grade glioma
PEG: polyethylene glycol
RIPK2: receptor-interacting serine/threonine-protein kinase 2
SIK3: salt-inducible kinase 3
SMI: small molecule inhibitor
S_N_Ar: nucleophilic aromatic substitution
STAB: sodium triacetoxyborohydride
T3P: Propylphosphonic anhydride
TGF-β: transforming growth factor-beta
THF: tetrahydrofuran
TNIK: TRAF2 and NCK-interacting protein kinase
TPD: targeted protein degradation
VHL: von Hippel-Lindau
