# Supplementary material for: Targeting Activin Receptor-like Kinase 2 Using Heterobifunctional Protein Degraders
Source: J Med Chem. 2026 May 4;69(9):11524–46. doi: 10.1021/acs.jmedchem.6c00714 (PMC13181787; doi:10.1021/acs.jmedchem.6c00714)
Supplement: Supplementary file 1 [file jm6c00714_si_001.pdf]

Supporting Information

Targeting Activin Receptor-like Kinase 2 Using Heterobifunctional Protein Degraders

Daniel T. Webb[£,$], Katherine L. Jones[$]*, Natsuko Macabuag[$], Ruzica Bago[$], Joshua Betts[$], Sumit Bhattacharyya[$], Steve Clifton[$], Ryan A. J. Tinson[$], Chigozie Achara[$], Simon Gilbert[$], Stefanie Howell[€], David H. Drewry[€], Rebecca Rogers[&], Chris Jones[&], Kyle R. Ferguson[^], Alex N. Bullock[^], David M. Lindsay[£], William J. Kerr[£]* and William Esmieu[$].

[£]Department of Pure and Applied Chemistry, University of Strathclyde, Glasgow, G1 1XL, United Kingdom.

[$]Discovery from Charles River, Charles River, Chesterford Research Park, Saffron Walden CB10 1XL, United Kingdom.

[€]Structural Genomics Consortium (SGC) and Division of Chemical Biology and Medicinal Chemistry, Eshelman School of Pharmacy and Lineberger Comprehensive Cancer Center, University of North Carolina at Chapel Hill, Chapel Hill, North Carolina 27599, United States.

[&]Division of Molecular Pathology and Division of Cancer Therapeutics, The Institute of Cancer Research, London, SM2 5NG, United Kingdom.

[^]Centre for Medicines Discovery, Nuffield Department of Medicine, University of Oxford, Oxford OX3 7FZ, United Kingdom.

Corresponding Authors

Katherine L. Jones - Discovery from Charles River, Charles River, Chesterford Research Park, Saffron Walden CB10 1XL, United Kingdom; Email: Katherine.jones@crl.com
William J. Kerr - Department of Pure and Applied Chemistry, University of Strathclyde, Glasgow, G1 1XL, United Kingdom; Email: w.kerr@strath.ac.uk

Contents

1. Supplementary Figures……S2
2. Uncropped Immunoblots, Quantification, and Statistical Analysis……S6
3. Analytical Spectra for Key Intermediates and Final Compounds……S13
4. References……S50

1. Supplementary figures

Figure S1: ALK2 NanoBRET Assay Results

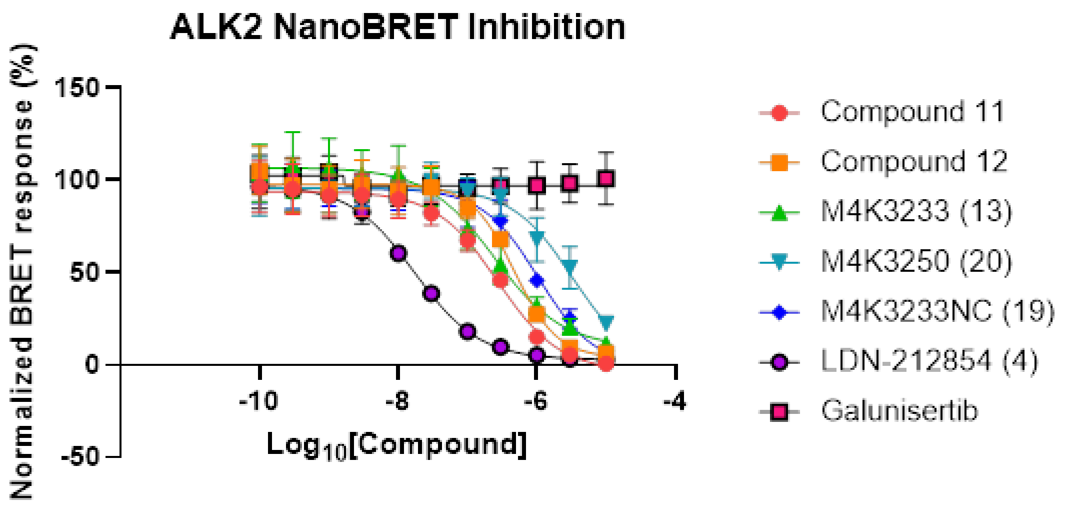

Figure S1: ALK2 NanoBRET data in HEK-293 cells for selected ALK2 degraders, as well as the negative control degrader compound **19**, the selective ALK2 inhibitor LDN-212854 (**4**)[1], and the selective ALK5 inhibitor Galunisertib[2]. Data plotted is the mean ± SEM of three biologically independent replicates using GraphPad Prism 9.

Figure S2: ALK5 NanoBRET Assay Results

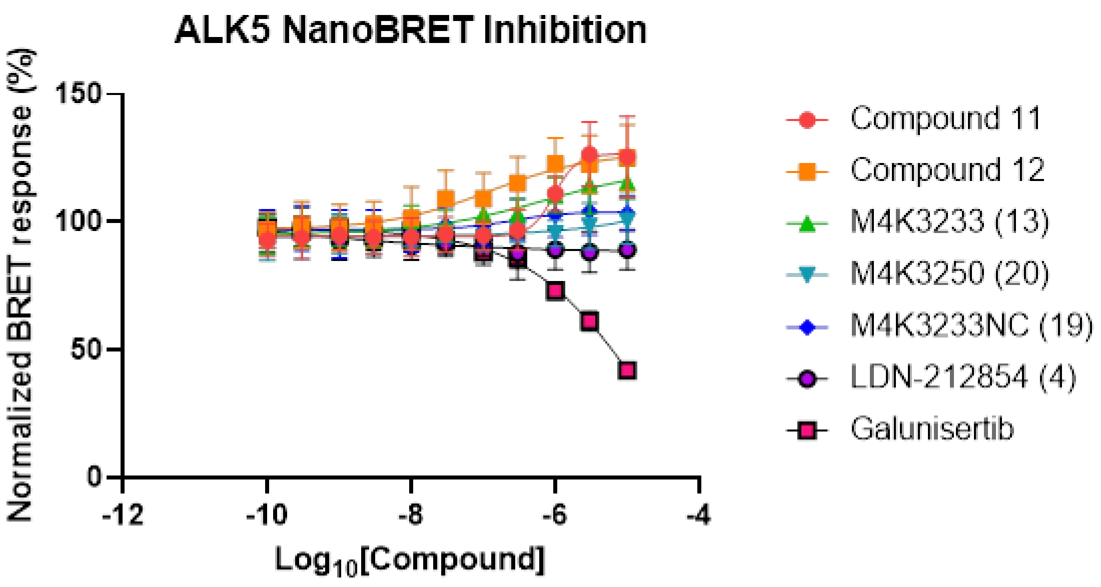

Figure S2: ALK5 NanoBRET data in HEK-293 cells for selected ALK2 degraders, as well as the negative control degrader compound **19**, the selective ALK2 inhibitor LDN-212854 (**4**)[1], and the selective ALK5 inhibitor Galunisertib[2]. Data plotted is the mean ± SEM of three biologically independent replicates using GraphPad Prism 9.

Figure S3: Western Blot Screening of Second and Third Generation ALK2 Degraders

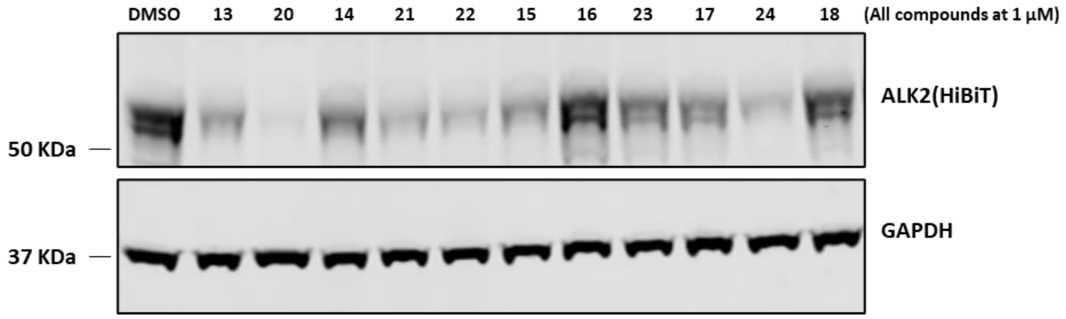

Figure S3: Western blot screening of second and third generation ALK2 degraders. ALK2-HiBiT transfected HEK-293 cells were treated for 24 h with each compound at a concentration of 1 μM. The cells were lysed and normalised ALK2(HiBiT) expression was measured using GAPDH as a loading control. Two biologically independent experiments were conducted per compound treatment.

Figure S4: Paediatric Diffuse High-grade Glioma Cell Line Information

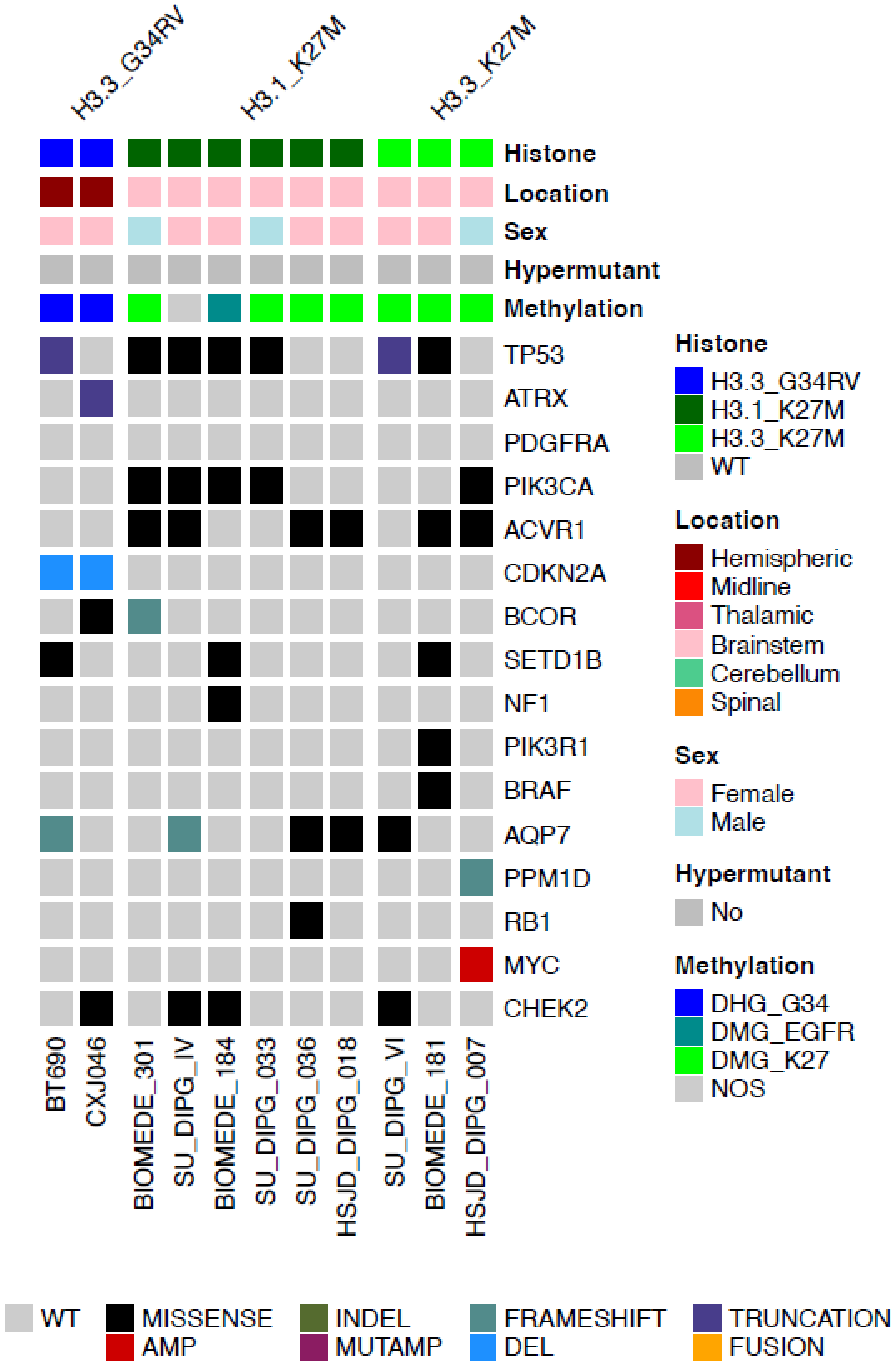

Figure S5: Summarized Cell Viability Data for **M4K3233** (**13**) in Different Cell Lines

**Cell Type**	**Cell Line**	**GI_{50} (μM)**
***ACVR1*-mutant diffuse midline glioma**	**ICR-B181**	**0.35**
	**HSJD-DIPG-018**	**5.2**
	**ICR-B301**	**0.39**
	**SU-DIPG-IV**	**0.86**
	**SU-DIPG-36**	**0.45**
	**HSJD-DIPG-007**	**0.31**
***ACVR1*-wt diffuse midline glioma**	**SU-DIPG-VI**	**0.90**
	**SU-DIPG-33**	**1.1**
	**ICR-B184**	**0.49**
**G34 diffuse high-grade glioma**	**BT690**	**0.67**
	**CXJ046**	**4.1**
**Glioblastoma**	**U87-MG**	**1.3**
**Acute myeloid leukaemia**	**MOLM13**	**0.33**
	**MV4-11**	**16**
	**U-937**	**>30**
**Ovarian cancer**	**SK-OV-3**	**18**

2. Uncropped Immunoblots, Quantification and Statistical Analysis

Quantification of Figure 2:

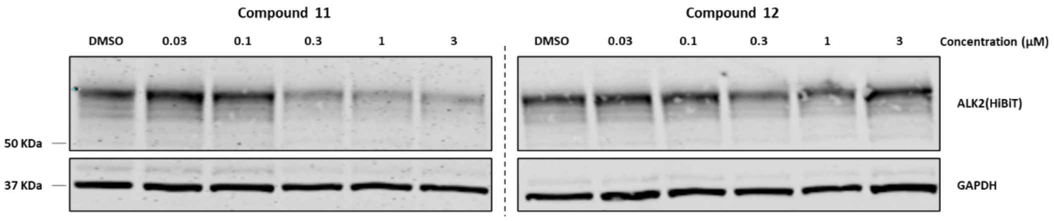

Compound 11						
n=1	DMSO	0.03	0.1	0.3	1	3
ALK2(HiBiT)	129000	117000	78242	76700	77700	59860
GAPDH	290000	337000	266000	331000	386000	312000
ALK2(HiBiT)/GAPDH	0.444828	0.347181	0.294143	0.231722	0.201295	0.191859
%DMSO	100%	78%	66%	52%	45%	43%
n=2	DMSO	0.03	0.1	0.3	1	3
ALK2(HiBiT)	1250000	1080000	980000	598000	567000	501000
GAPDH	2840000	3190000	3580000	2580000	2830000	2750000
ALK2(HiBiT)/GAPDH	0.440141	0.338558	0.273743	0.231783	0.200353	0.182182
%DMSO	100%	77%	62%	53%	46%	41%
**Unpaired two-tailed Student's t-test**		0.03	0.1	0.3	1	3
P-Value:		0.0005	0.0031	0.0001	<0.0001	0.0003
Significance:		***	**	***	****	***

Compound 12						
n=1	DMSO	0.03	0.1	0.3	1	3
ALK2(HiBiT)	580000	331953	381695	310356	247830	261255
GAPDH	1960837	1614467	1430373	1476208	1405973	1516959
ALK2(HiBiT)/GAPDH	0.295792	0.205612	0.26685	0.210239	0.176269	0.172223
%DMSO	100%	70%	90%	71%	60%	58%
n=2	DMSO	0.03	0.1	0.3	1	3
ALK2(HiBiT)	1827646	1752756	1504656	1125413	998697	1966273
GAPDH	2715780	3143144	2988377	2660232	2390427	4558775
ALK2(HiBiT)/GAPDH	0.672973	0.557644	0.503503	0.423051	0.41779	0.431316
%DMSO	100%	83%	75%	63%	62%	64%
**Unpaired two-tailed Student's t-test**		0.03	0.1	0.3	1	3
P-Value:		0.0687	0.1448	0.0144	0.0007	0.0059
Significance:		ns	ns	*	***	**

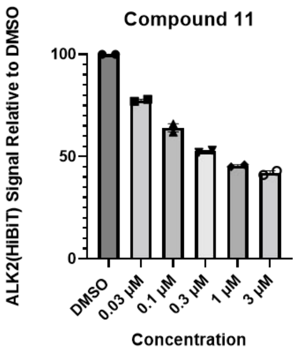

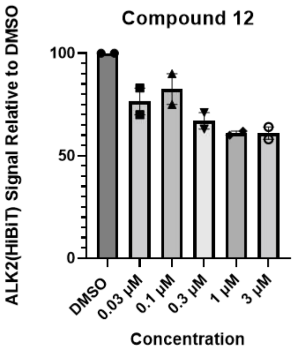

Compound 11

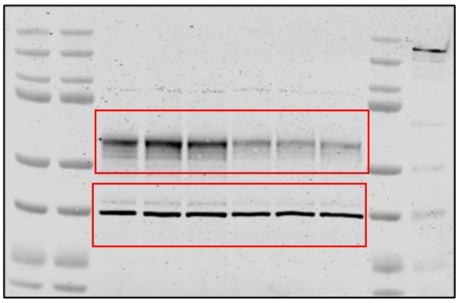

Compound 12

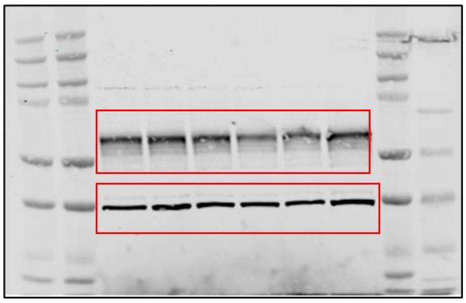

Quantification of Figure S3:

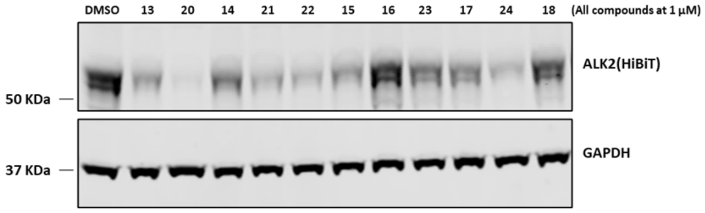

	ALK2(HiBiT) Western Blot Screening											
n=1	DMSO	13	20	14	21	22	15	16	23	17	24	18
ALK2(HiBiT)	5190000	1260000	267000	2540000	1470000	753000	2010000	3570000	2500000	3580000	2670000	2360000
GAPDH	4420000	3870000	4180000	3720000	3410000	3360000	3420000	3760000	3650000	3400000	3560000	3430000
ALK2(HiBiT)/GAPDH	1.174208	0.325581	0.063876	0.682796	0.431085	0.224107	0.587719	0.949468	0.684932	1.052941	0.75	0.688047
%DMSO	100%	28%	5%	58%	37%	19%	50%	81%	58%	90%	64%	59%
n=2	DMSO	13	20	14	21	22	15	16	23	17	24	18
ALK2(HiBiT)	1470000	336000	57100	581000	250000	225000	366000	1200000	559000	535000	232000	903000
GAPDH	3540000	3200000	3720000	2970000	3050000	2980000	3000000	3160000	2920000	2900000	2910000	3240000
ALK2(HiBiT)/GAPDH	0.415254	0.105	0.015349	0.195623	0.081967	0.075503	0.122	0.379747	0.191438	0.184483	0.079725	0.278704
%DMSO	100%	25%	4%	47%	20%	18%	29%	91%	46%	44%	19%	67%
**Unpaired two-tailed Student's t-test**		13	20	14	21	22	15	16	23	17	24	18
P-value:		0.0004	<0.0001	0.0131	0.0138	<0.0001	0.0288	0.1074	0.0153	0.2878	0.1215	0.0115
Significance:		***	****	*	*	****	*	ns	*	ns	ns	*

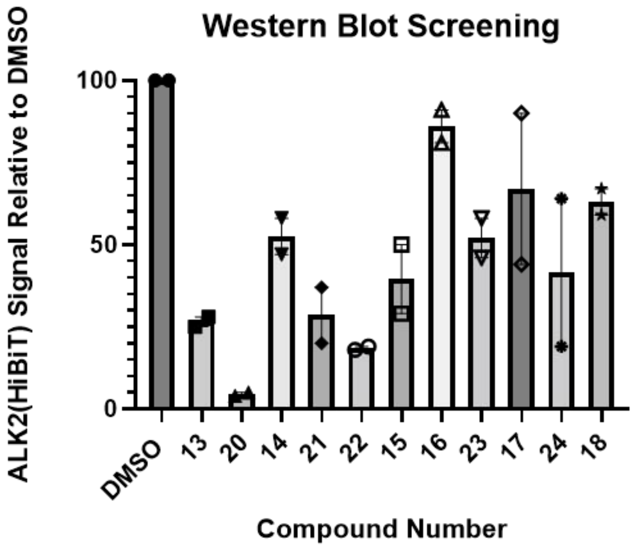

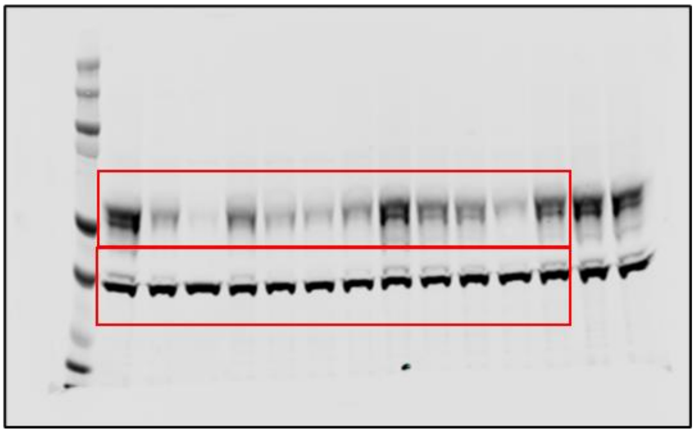

Quantification of Figure 4:

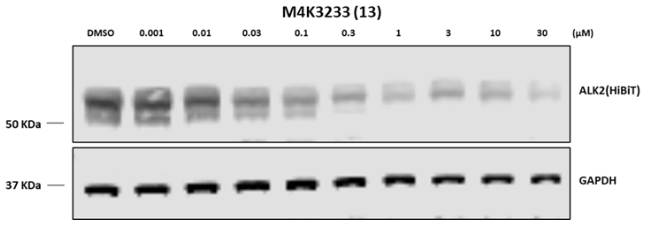

Compound 13										
n=1	DMSO	1 nM	10 nM	30 nM	100 nM	300 nM	1000 nM	3000 nM	10000 nM	30000 nM
ALK2(HiBiT)	298000	247000	234000	182000	151000	120000	89400	109000	82300	68600
GAPDH	646000	664000	697000	721000	704000	632000	540000	490000	456000	410000
ALK2(HiBiT)/GAPDH	0.4613	0.371988	0.335725	0.252427	0.214489	0.189873	0.165556	0.222449	0.180482	0.167317
%DMSO	100%	81%	73%	55%	46%	41%	36%	48%	39%	36%
n=2	DMSO	1 nM	10 nM	30 nM	100 nM	300 nM	1000 nM	3000 nM	10000 nM	30000 nM
ALK2(HiBiT)	604000	593000	610000	389000	239000	322000	266000	155000	151000	198000
GAPDH	106000	100000	128000	130000	108000	116000	120000	78000	97300	92000
ALK2(HiBiT)/GAPDH	5.698113	5.93	4.765625	2.992308	2.212963	2.775862	2.216667	1.987179	1.551901	2.152174
%DMSO	100%	104%	84%	53%	39%	49%	39%	35%	27%	38%
n=3	DMSO	1 nM	10 nM	30 nM	100 nM	300 nM	1000 nM	3000 nM	10000 nM	30000 nM
ALK2(HiBiT)	138000	153000	126000	98600	90400	66200	49800	61400	56900	58600
GAPDH	230000	297000	309000	315000	401000	336000	255000	251000	226000	265000
ALK2(HiBiT)/GAPDH	0.6	0.515152	0.407767	0.313016	0.225436	0.197024	0.195294	0.244622	0.25177	0.221132
%DMSO	100%	86%	68%	52%	38%	33%	33%	41%	42%	37%
**Unpaired two-tailed Student's t-test**		1 nM	10 nM	30 nM	100 nM	300 nM	1000 nM	3000 nM	10000 nM	30000 nM
P-Value:		0.2385	0.0061	<0.0001	<0.0001	0.0002	<0.0001	<0.0001	0.0002	<0.0001
Significance:		ns	**	****	****	***	****	****	***	****

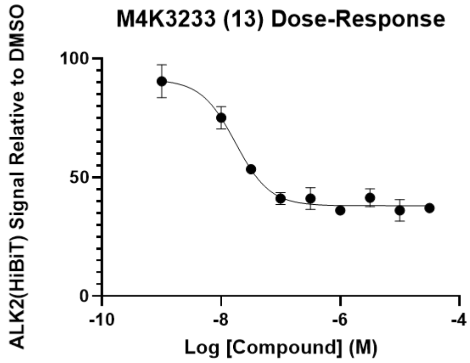

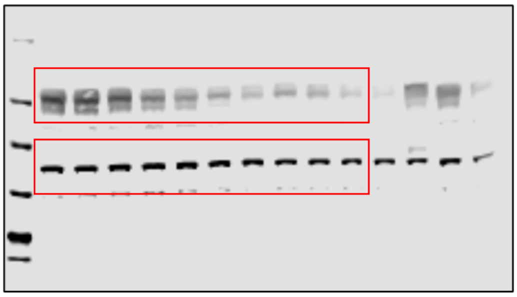

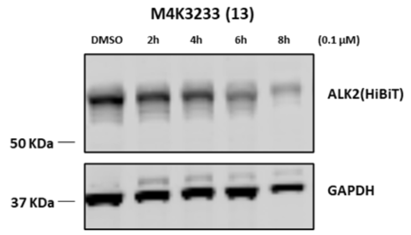

M4K3233 Time-Course					
n=1	DMSO	2 h	4 h	6 h	8 h
ALK2(HiBiT)	122000	63300	58600	48300	16900
GAPDH	419000	265000	254000	266000	116000
ALK2(HiBiT)/GAPDH	0.291169	0.238868	0.230709	0.181579	0.14569
%DMSO	100%	82%	79%	62%	50%
n=2	DMSO	2 h	4 h	6 h	8 h
ALK2(HiBiT)	110000	68400	59900	39700	44800
GAPDH	366000	269000	269000	217000	283000
ALK2(HiBiT)/GAPDH	0.300546	0.254275	0.222677	0.182949	0.158304
%DMSO	100%	85%	74%	61%	53%
**Unpaired two-tailed Student's t-test**		2 h	4 h	6 h	8 h
P-Value		0.0082	0.0111	0.0002	0.001
Significance		**	*	***	***

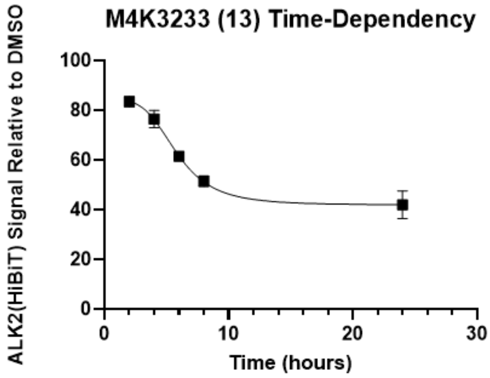

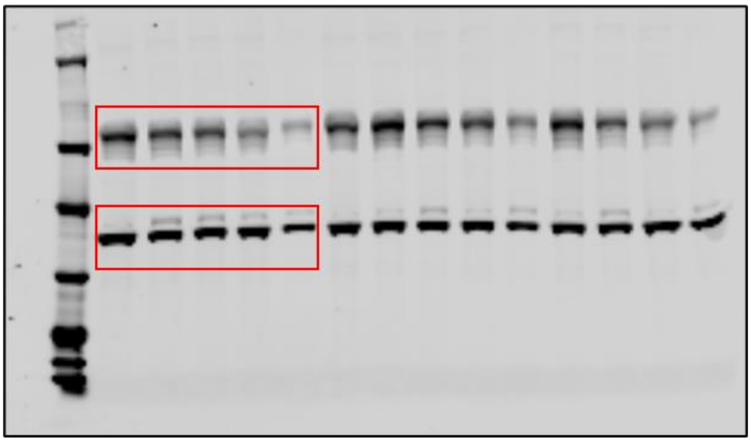

Quantification of Figure 6:

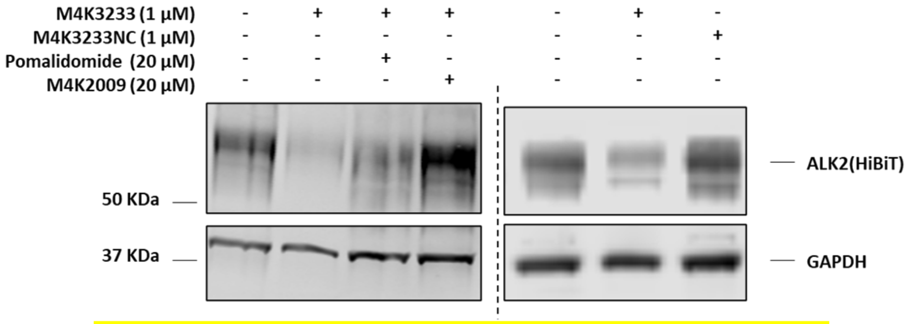

CRBN / ALK2 Dependency Experiments								
n=1	DMSO	M4K3233	M4K3233+ POM	M4K3233+ M4K2009		DMSO	M4K3233	M4K3233NC
ALK2(HiBiT)	380000	80600	302000	628000		146000	39800	158000
GAPDH	2040000	1690000	2660000	2890000		883000	786000	927000
ALK2(HiBiT)/GAPDH	0.186275	0.047692	0.113534	0.217301		0.165345	0.050636	0.170442287
%DMSO	100%	26%	61%	117%		100%	31%	103%
n=2	DMSO	M4K3233	M4K3233+ POM	M4K3233+ M4K2009		DMSO	M4K3233	M4K3233NC
ALK2(HiBiT)	385000	190000	271000	310000		171000	29400	209000
GAPDH	1980000	2760000	1770000	1980000		781000	609000	947000
ALK2(HiBiT)/GAPDH	0.194444	0.068841	0.153107	0.1565657		0.21895	0.048276	0.220696938
%DMSO	100%	35%	79%	81%		100%	22%	101%
**Unpaired two-tailed Student's t-test**		M4K3233	M4K3233+ POM	M4K3233+ M4K2009			M4K3233	M4K3233NC
P-Value:		0.0042	0.0794	0.9607			0.0037	0.1835
Significance:		**	ns	ns			**	ns

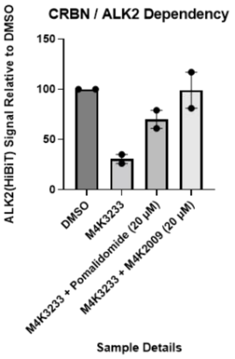

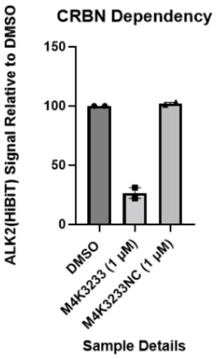

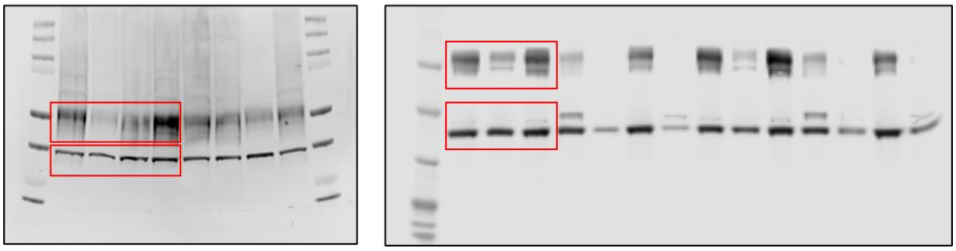

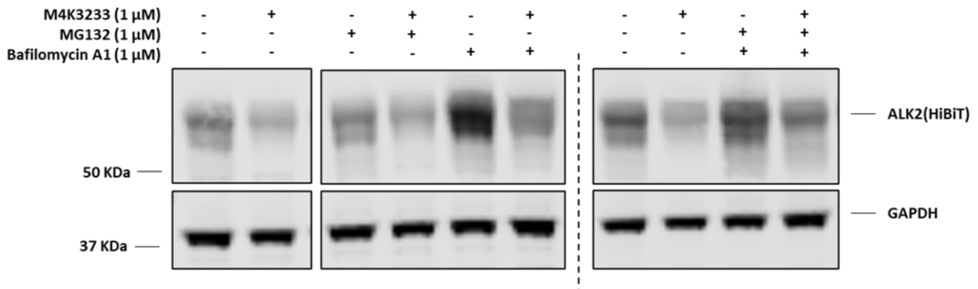

Proteasome / Lysosome Dependency Experiments											
n=1	DMSO	M4K3233	MG132	M4K3233 +MG132	Baf A1	M4K3233 +BafA1		DMSO	M4K3233	MG132+ Baf A1	M4K3233 +MG132+ Baf A1
ALK2(HiBiT)	104000	22600	71000	51700	181000	93900		374000	70600	516000	325000
GAPDH	140000	111000	89200	111000	171000	162000		696000	596000	653000	674000
ALK2(HiBiT)/GAPDH	0.742857	0.203604	0.795964	0.465766	1.05848	0.57963		0.537356	0.118456	0.7902	0.482196
%DMSO	100%	27%	107%	63%	142%	78%		100%	22%	147%	90%
n=2	DMSO	M4K3233	MG132	M4K3233 +MG132	Baf A1	M4K3233 +BafA1		DMSO	M4K3233	MG132+ Baf A1	M4K3233 +MG132+ Baf A1
ALK2(HiBiT)	112000	27700	97000	51900	216000	99600		377000	128000	387000	234000
GAPDH	312000	269000	246000	266000	342000	302000		1080000	838000	782000	694000
ALK2(HiBiT)/GAPDH	0.358974	0.102974	0.394309	0.195113	0.631579	0.329801		0.349074	0.152745	0.49488	0.337176
%DMSO	100%	29%	110%	54%	176%	92%		100%	44%	142%	97%
**Unpaired two-tailed Student's t-test**		M4K3233	MG132	M4K3233 +MG132	Baf A1	M4K3233 +BafA1			M4K3233	MG132+ Baf A1	M4K3233 +MG132+ Baf A1
P-Value:		0.0002	0.0298	0.0116	0.0739	0.1654			0.0259	0.0031	0.2044
Significance:		***	*	*	ns	ns			*	**	ns

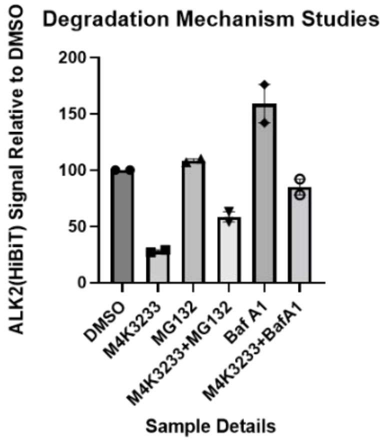

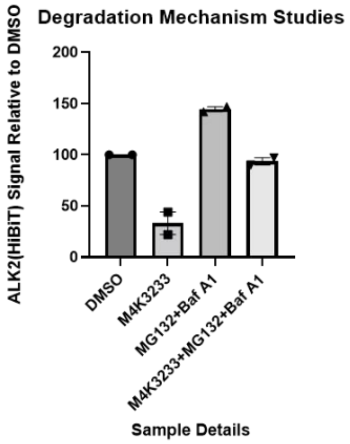

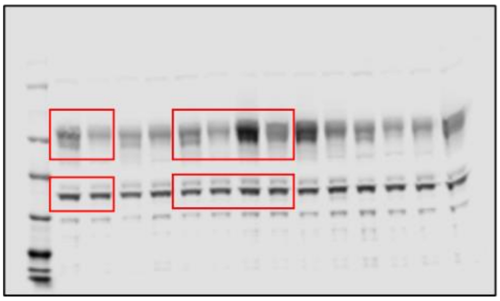

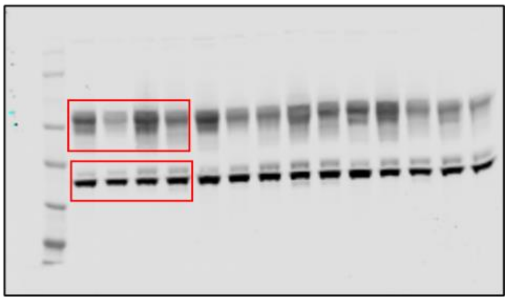

Quantification of Figure 8

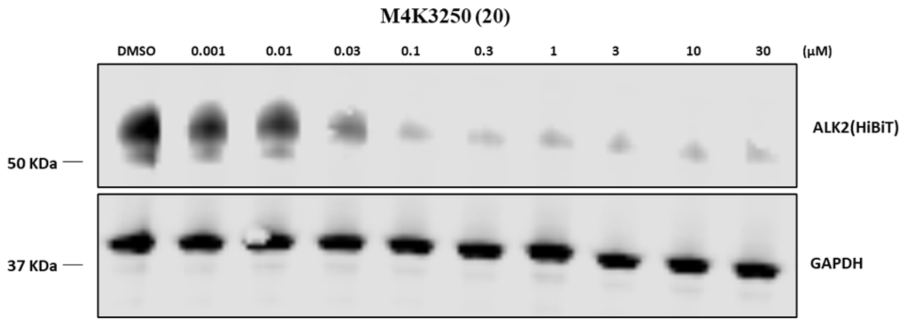

Compound 20										
n=1	DMSO	1 nM	10 nM	30 nM	100 nM	300 nM	1000 nM	3000 nM	10000 nM	30000 nM
ALK2(HiBiT)	1020000	820000	749000	453000	423000	301000	293000	273000	298000	256000
GAPDH	365000	307000	275000	317000	316000	318000	402000	275000	297000	296000
ALK2(HiBiT)/GAPDH	2.794521	2.67101	2.723636	1.429022	1.338608	0.946541	0.728856	0.992727	1.003367	0.864865
%DMSO	100%	96%	97%	51%	48%	34%	26%	36%	36%	31%
n=2	DMSO	1 nM	10 nM	30 nM	100 nM	300 nM	1000 nM	3000 nM	10000 nM	30000 nM
ALK2(HiBiT)	1080000	776000	855000	376000	359000	350000	281000	339000	315000	295000
GAPDH	200000	168000	220000	147000	153000	188000	178000	208000	200000	217000
ALK2(HiBiT)/GAPDH	5.4	4.619048	3.886364	2.557823	2.346405	1.861702	1.578652	1.629808	1.575	1.359447
%DMSO	100%	86%	72%	47%	43%	34%	29%	30%	29%	25%
n=3	DMSO	1 nM	10 nM	30 nM	100 nM	300 nM	1000 nM	3000 nM	10000 nM	30000 nM
ALK2(HiBiT)	153000	259000	80400	71600	85800	40100	36200	20700	26100	24900
GAPDH	376000	779000	536000	448000	384000	425000	384000	384000	533000	644000
ALK2(HiBiT)/GAPDH	0.406915	0.332478	0.15	0.159821	0.223438	0.094353	0.094271	0.053906	0.048968	0.038665
%DMSO	100%	82%	37%	39%	55%	23%	23%	13%	12%	10%
**Unpaired two-tailed Student's t-test**		1 nM	10 nM	30 nM	100 nM	300 nM	1000 nM	3000 nM	10000 nM	30000 nM
P-Value:		0.0449	0.1461	0.0001	0.0001	<0.0001	<0.0001	0.0004	0.0005	0.0002
Significance:		*	ns	***	***	****	****	***	***	***

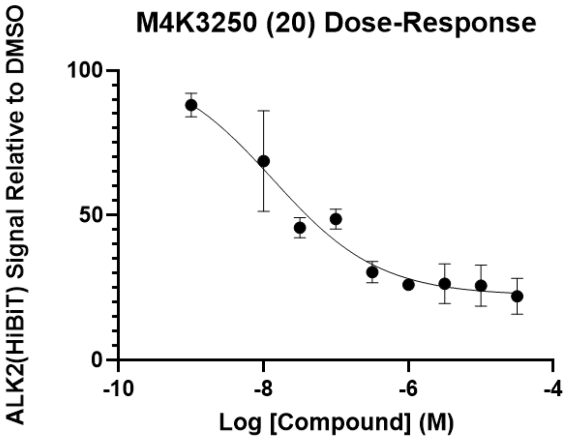

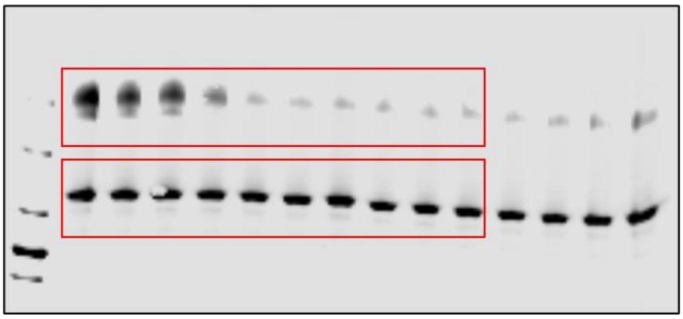

3. Analytical Spectra for Key Intermediates and Final Compounds:

Tert-butyl 4-(4-(5-bromo-4-methylpyridin-3-yl)phenyl)piperazine-1-carboxylate (**26**)

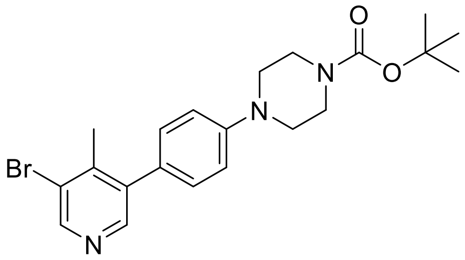

^{1}H NMR (500 MHz, CDCl$_3$)

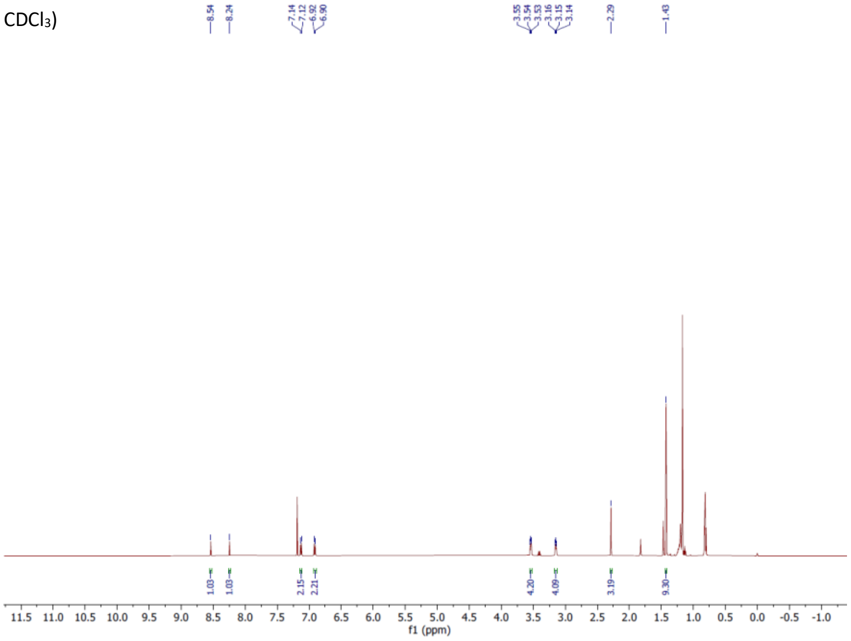

^{13}C NMR (126 MHz, CDCl$_3$)

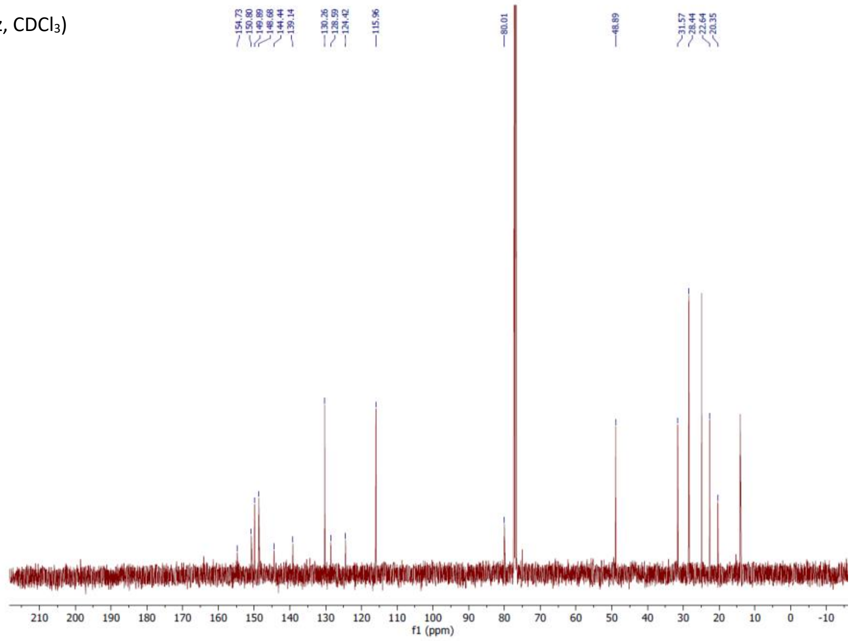

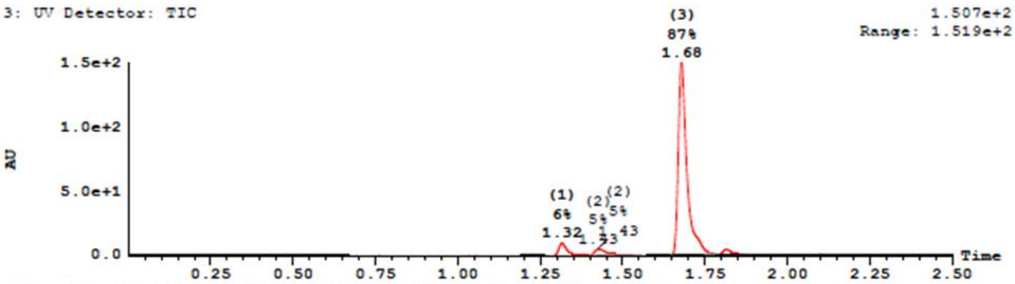

Peak Number	Compound	Time	AreaAbs	Area %Total	Width	Height	Mass Found
1		1.32	3e+005	5.96	0	1e+007	
2		1.43	2e+005	4.69	0	5e+006	
3		1.68	5e+006	86.85	0	2e+008	
4		1.81	1e+005	2.50	0	4e+006	

Peak ID	Compound	Time	Mass Found
3		1.68	

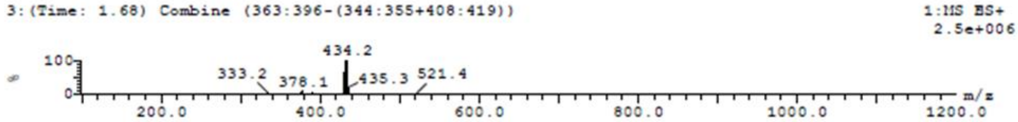

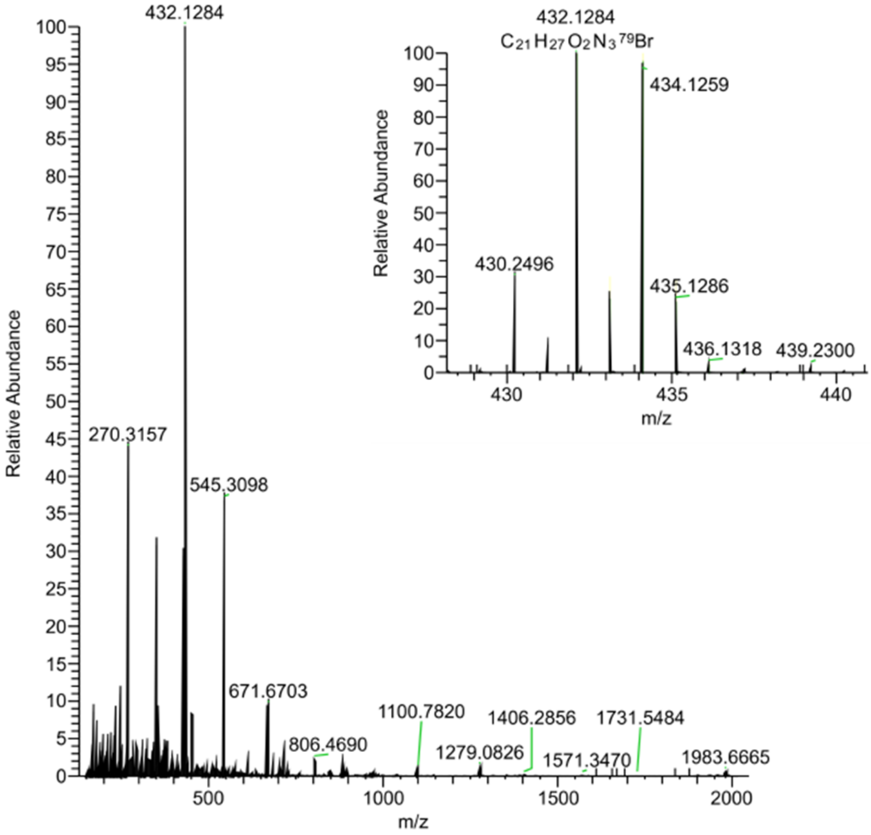

Tert-butyl 4-(4-(4-methyl-5-(3,4,5-trimethoxyphenyl)pyridin-3-yl)phenyl)piperazine-1-carboxylate (**27**)

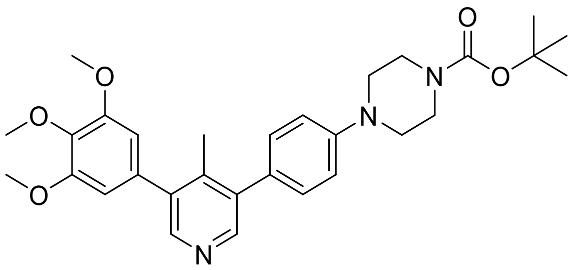

^{1}H NMR (400 MHz, d_6-DMSO)

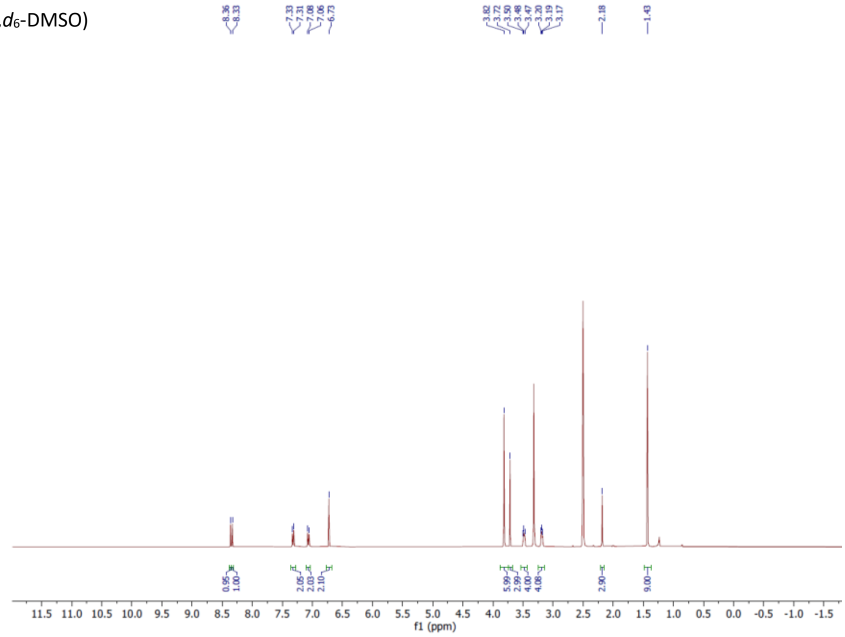

^{13}C NMR (101 MHz, d_6-DMSO)

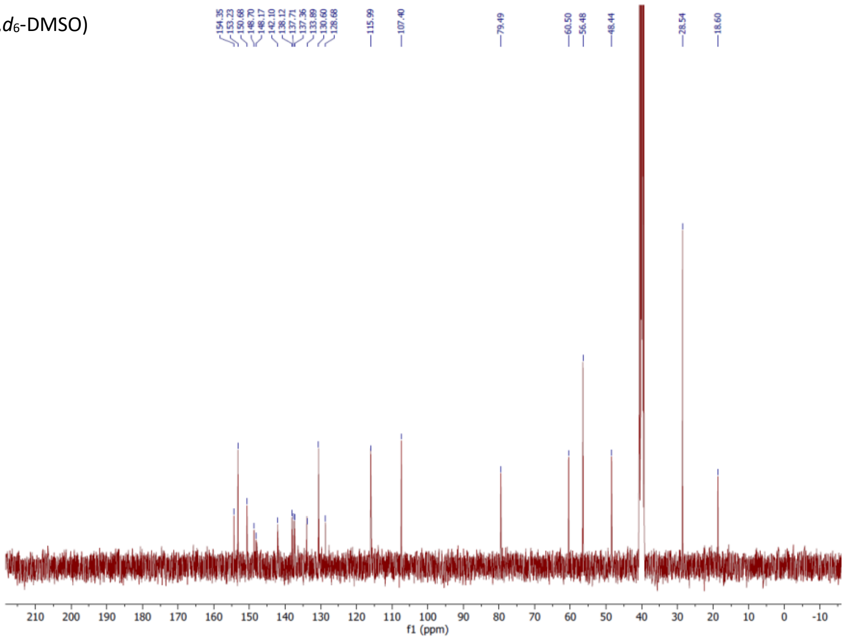

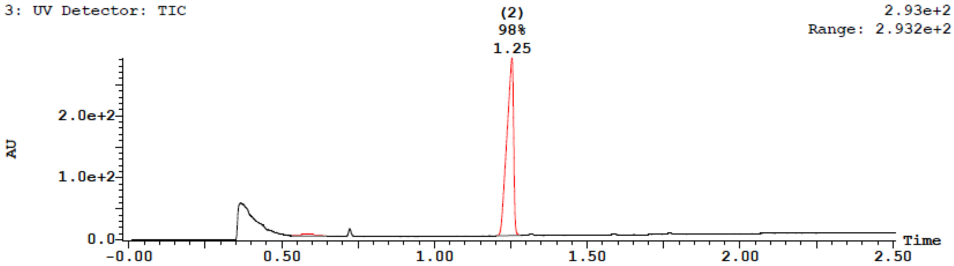

Peak Number	Compound	Time	AreaAbs	Area %Total	Width	Height	Mass Found
1		0.58	2e+005	2.49	0	4e+006	
2		1.25	7e+006	97.51	0	3e+008	

Peak ID	Compound	Time	Mass Found
1		0.58	

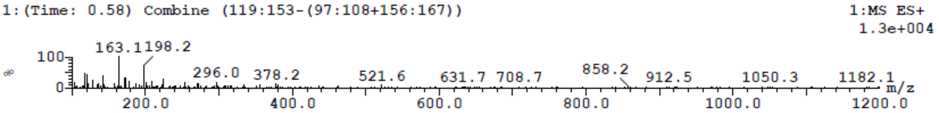

Peak ID	Compound	Time	Mass Found
2		1.25	

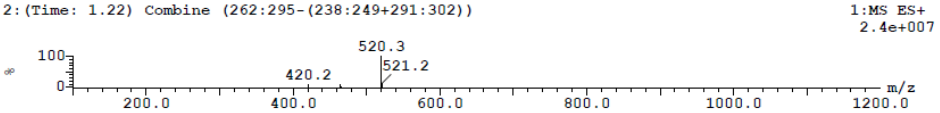

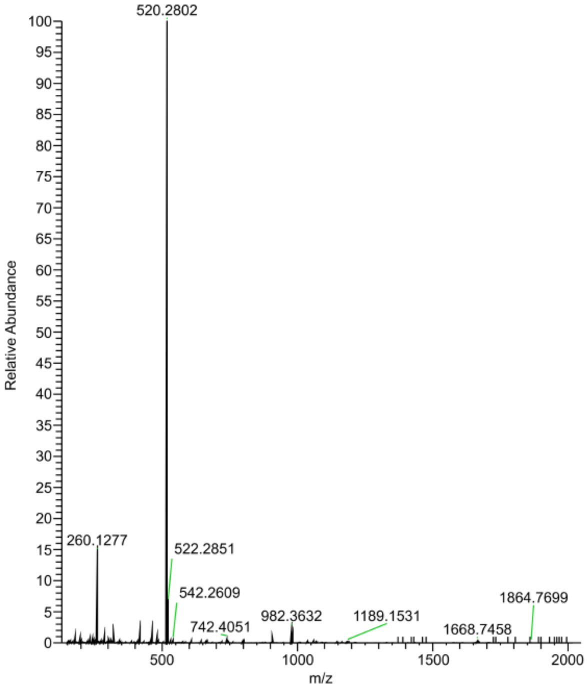

Tert-butyl 4-(4-(5-(4-fluoro-3,5-dimethoxyphenyl)-4-methylpyridin-3-yl)phenyl)piperazine-1-carboxylate (**28**)

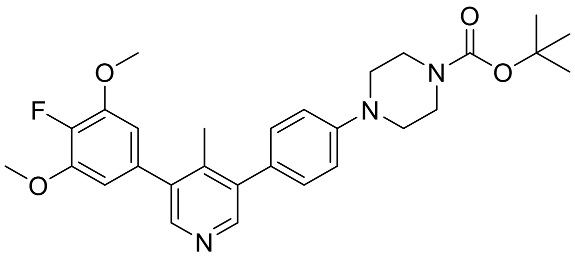

^{1}H NMR (400 MHz,CDCl$_3$)

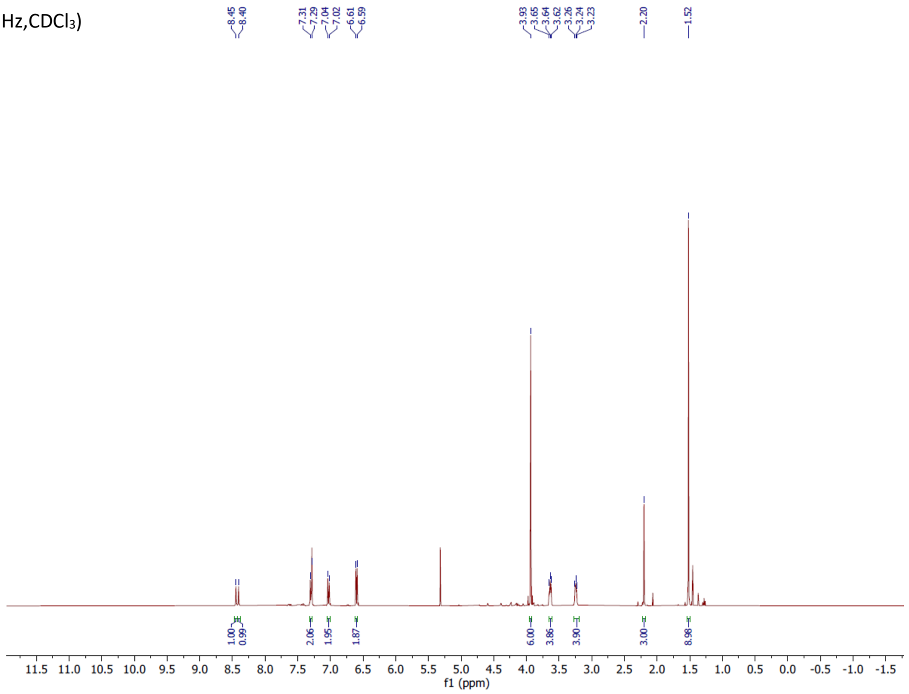

^{13}C NMR (101 MHz,CDCl$_3$)

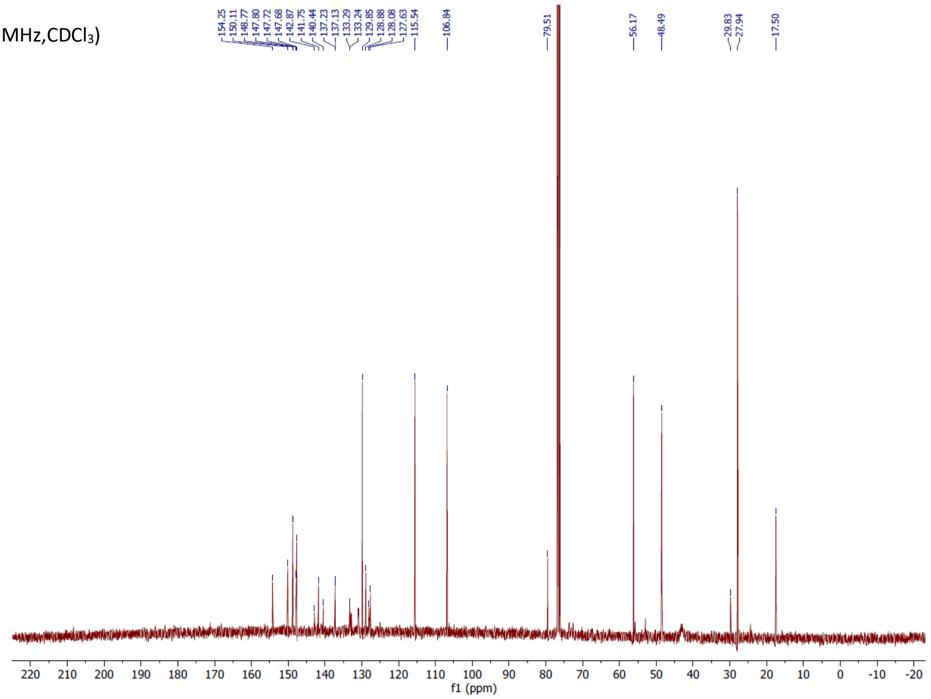

^{19}F NMR (376 MHz,$CDCl_3$)

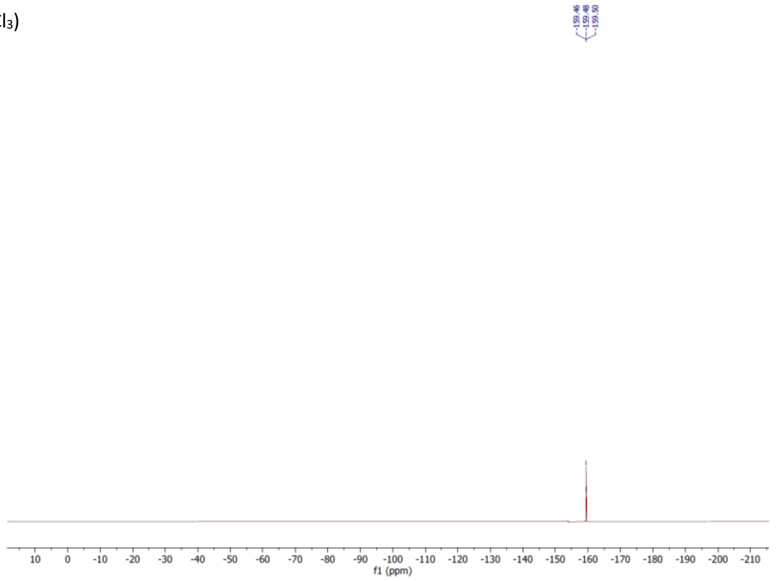

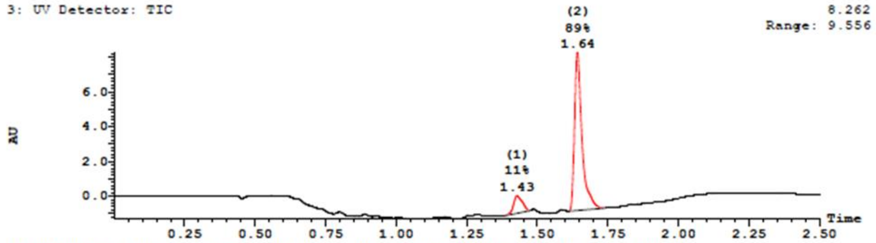

Peak Number	Compound	Time	AreaAbs	Area %Total	Width	Height	Mass Found
1		1.43	4e+004	11.39	0	1e+006	
2		1.64	3e+005	88.61	0	9e+006	

Peak ID	Compound	Time	Mass Found
2		1.64	

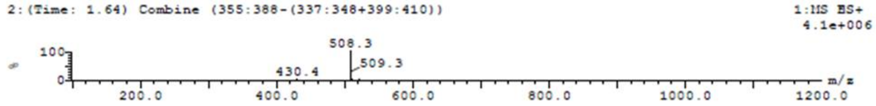

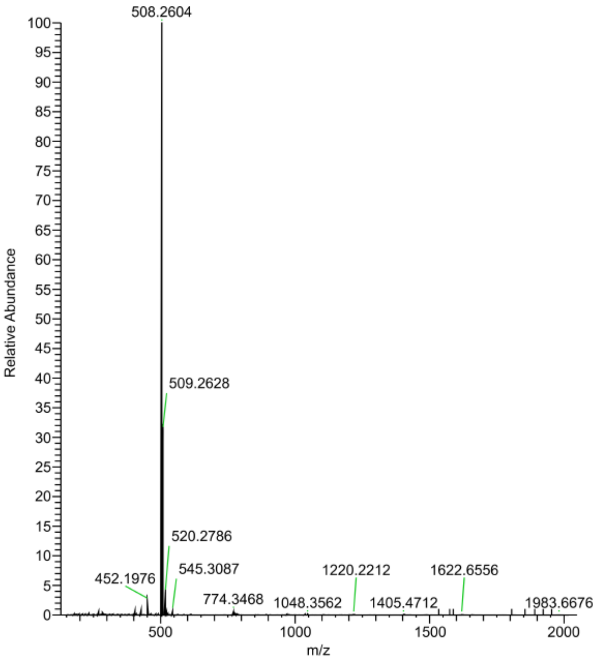

5-(2-(2-(2-(Benzyloxy)ethoxy)ethoxy)ethoxy)-2-(2,6-dioxopiperidin-3-yl)isoindoline-1,3-dione (**31**)

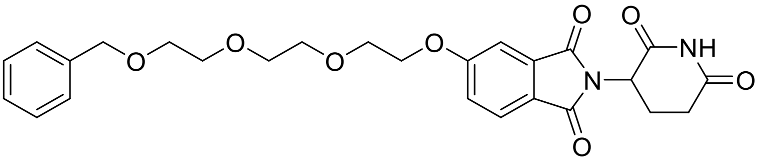

^{1}H NMR (400 MHz, $CDCl_3$)

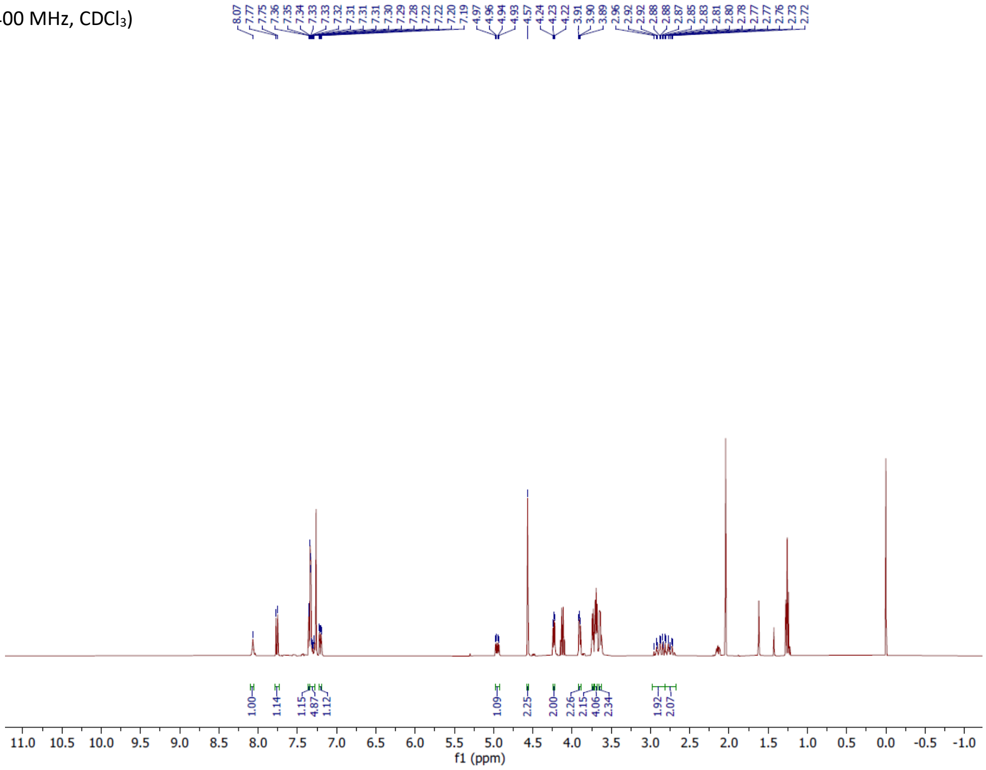

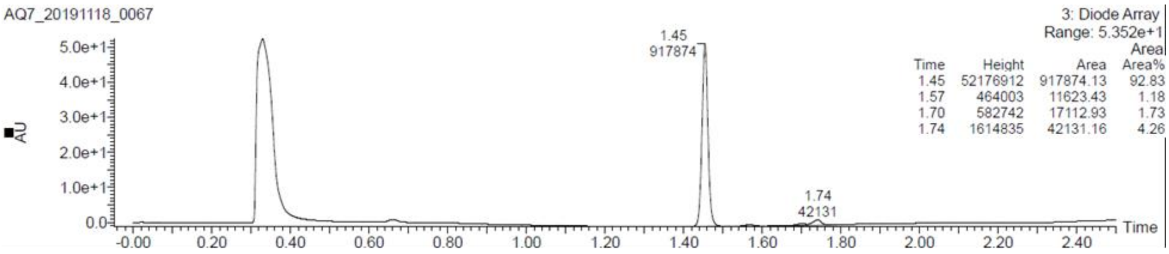

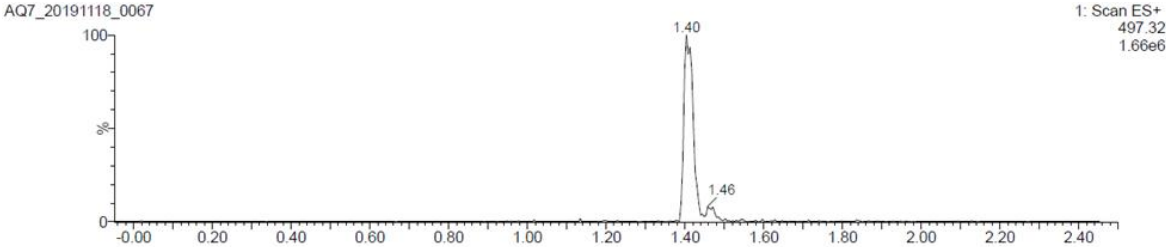

2-(2,6-Dioxopiperidin-3-yl)-5-(2-(2-(2-(4-(4-(4-methyl-5-(3,4,5-trimethoxyphenyl)20yridine-3-yl)phenyl)piperazin-1-yl)ethoxy)ethoxy)ethoxy)isoindoline-1,3-dione (**11**)

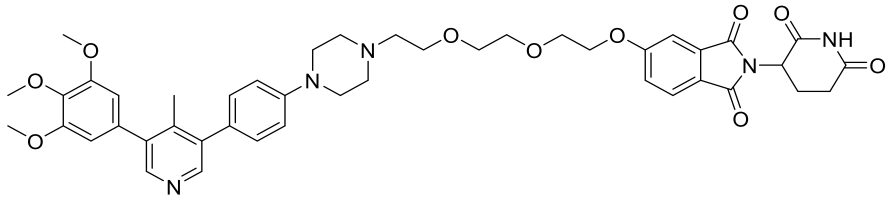

^{1}H NMR (400 MHz, d_6-DMSO)

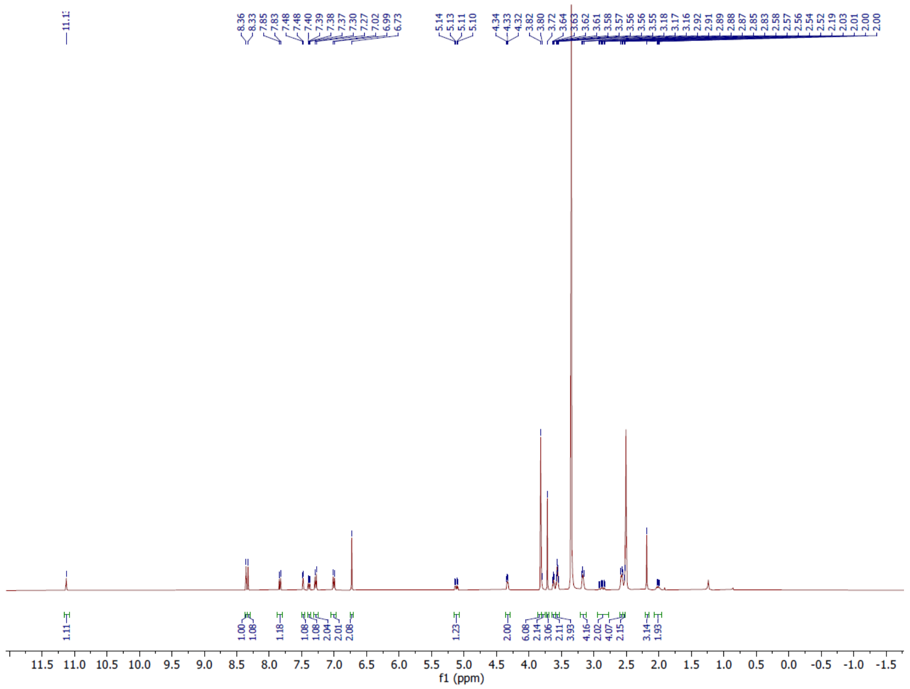

^{13}C NMR (101 MHz, d_6-DMSO)

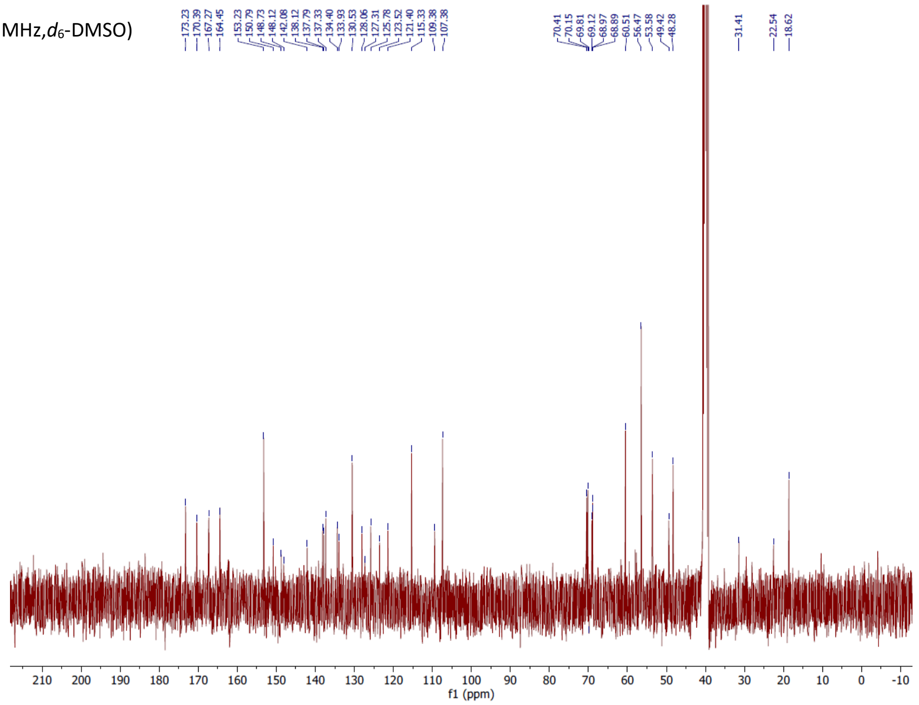

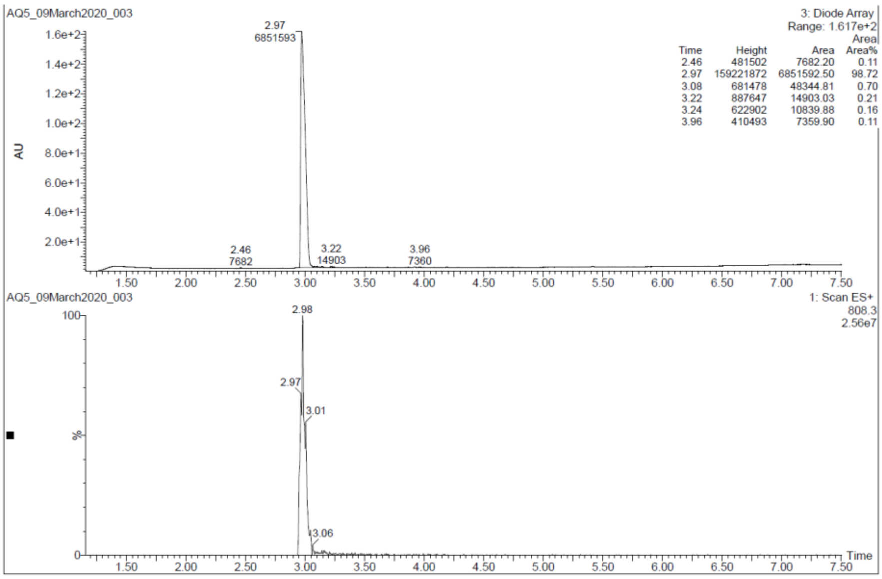
AQ5_09March2020_003
3: Diode Array
Range: 1.617e+2
Time Height Area Area%
2.46 481502 7682.20 0.11
2.97 159221872 6851592.50 98.72
3.08 681478 48344.81 0.70
3.22 887647 14903.03 0.21
3.24 622902 10839.88 0.16
3.96 410493 7359.90 0.11
AU
2.97
6851593
2.46
7682
3.22
14903
3.96
7360
AQ5_09March2020_003
1: Scan ES+
808.3
2.56e7
%
2.98
2.97
3.01
3.06
Time

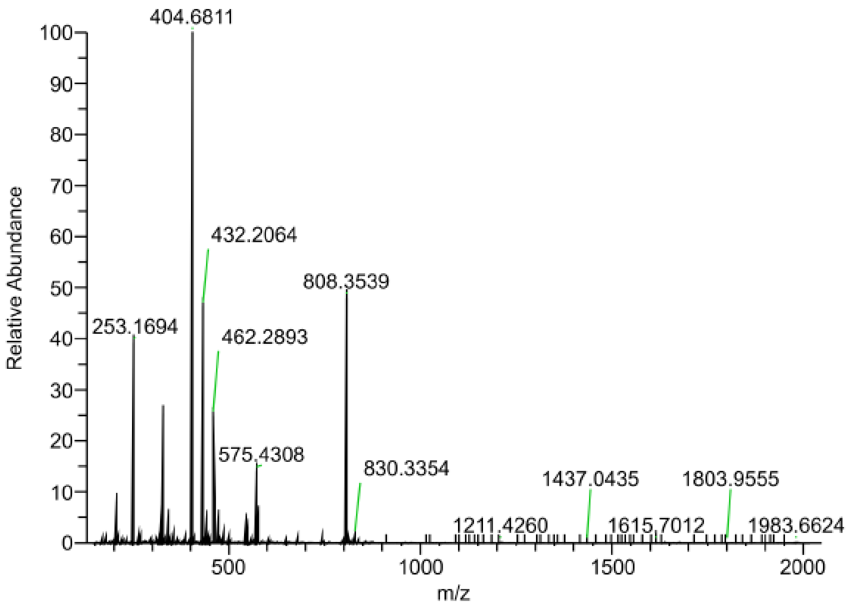
Relative Abundance
m/z
404.6811
432.2064
808.3539
253.1694
462.2893
575.4308
830.3354
1437.0435
1803.9555
1211.4260
1615.7012
1983.6624

Tert-butyl 2-[2-[2-(2-benzyloxyethoxy)ethoxy]ethoxy]acetate (**33**)

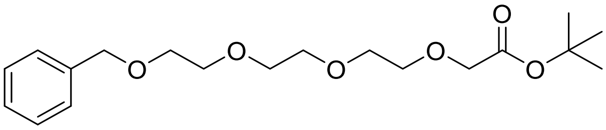

^{1}H NMR (400 MHz, d_6-DMSO)

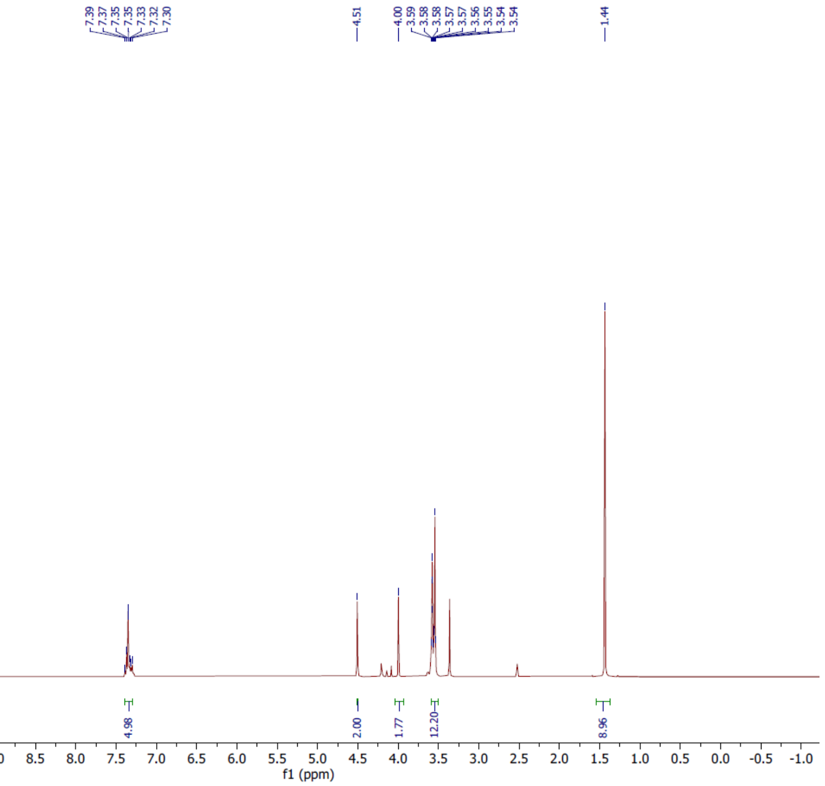

^{13}C NMR (400 MHz, d_6-DMSO)

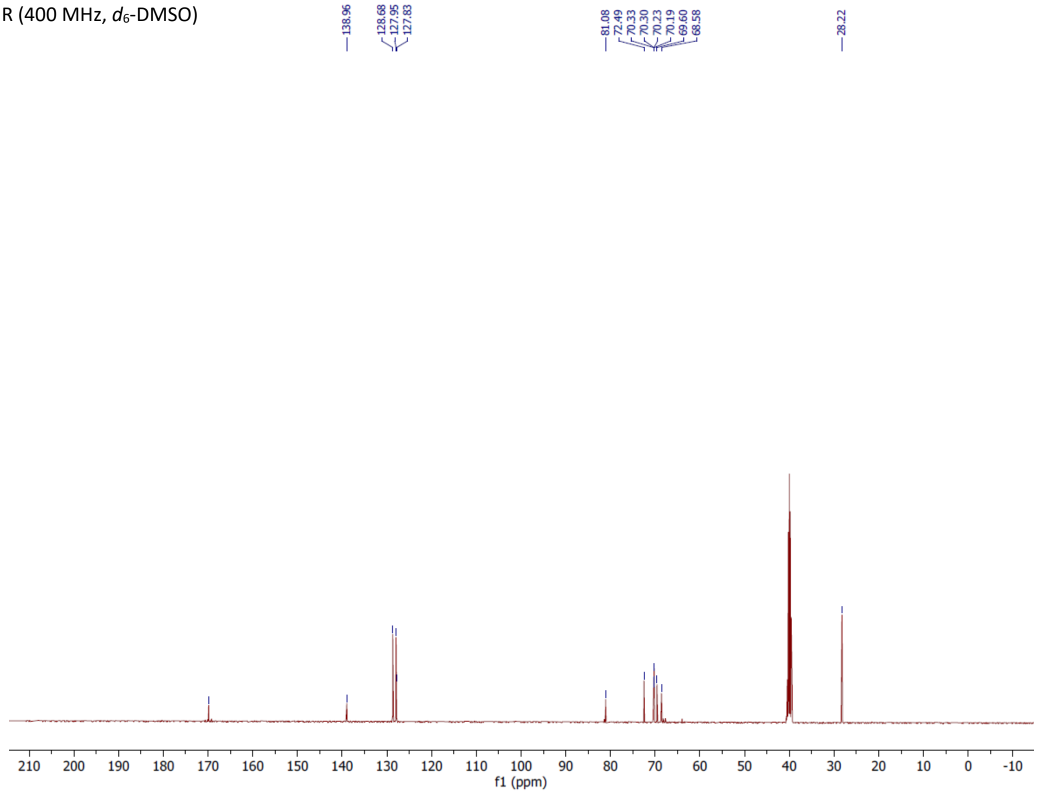

Tert-butyl 2-(2-(2-(2-(4-(4-(4-methyl-5-(3,4,5-trimethoxyphenyl)pyridin-3-yl)phenyl)piperazin-1-yl)ethoxy)ethoxy)ethoxy)acetate (**36**)

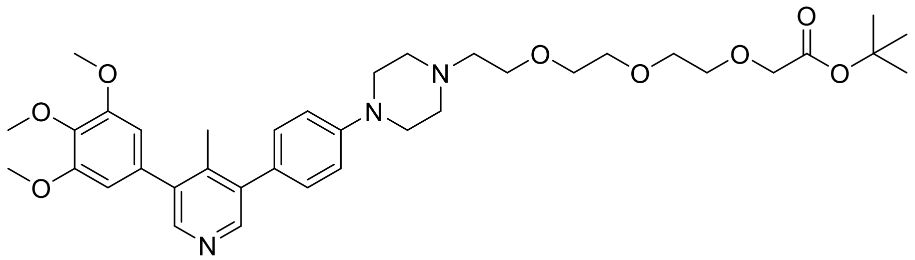

^{1}H NMR (400 MHz, $CDCl_3$)

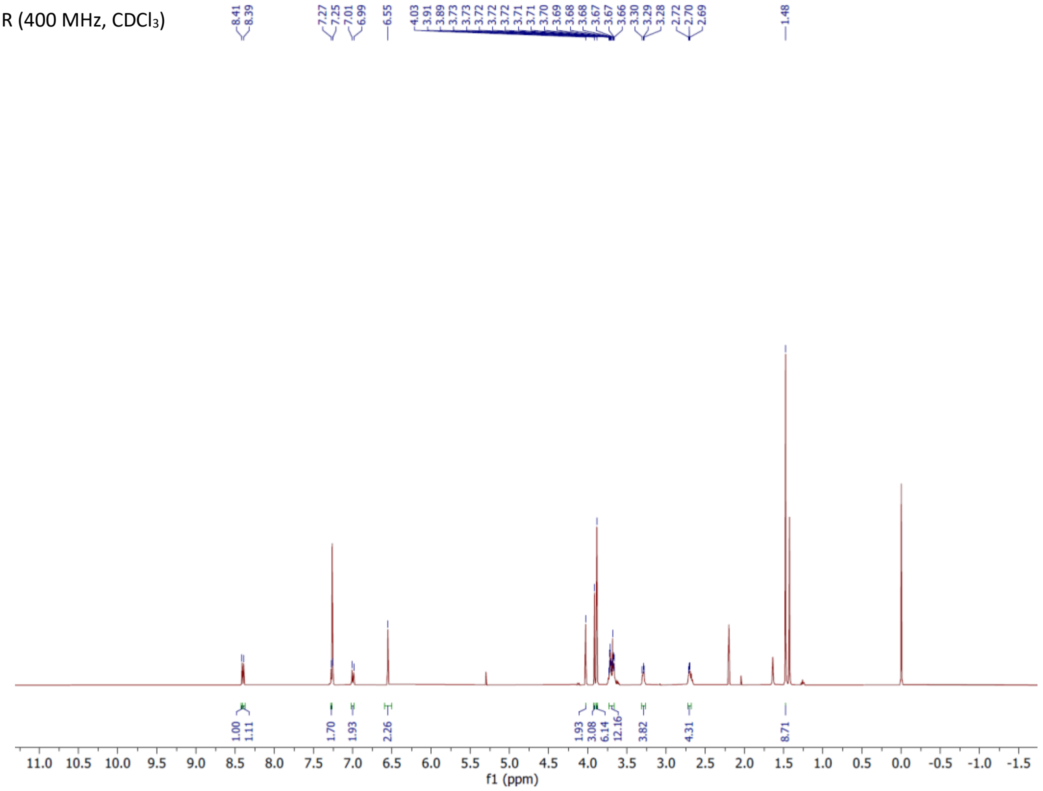

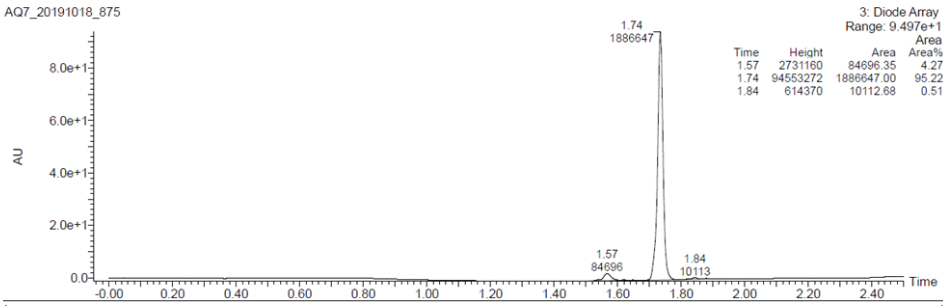

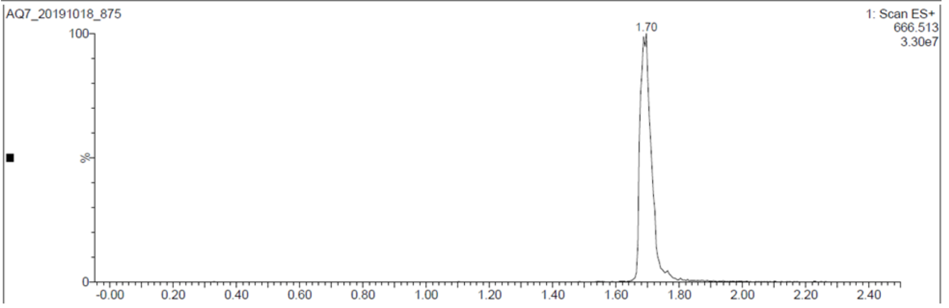

(2*S*,4*R*)-1-((*S*)-2-(*Tert*-butyl)-14-(4-(4-(4-methyl-5-(3,4,5-trimethoxyphenyl)pyridin-3-yl)phenyl)piperazin-1-yl)-4-oxo-6,9,12-trioxa-3-azatetradecanoyl)-4-hydroxy-N-(4-(4-methylthiazol-5-yl)benzyl)pyrrolidine-2-carboxamide (**12**)

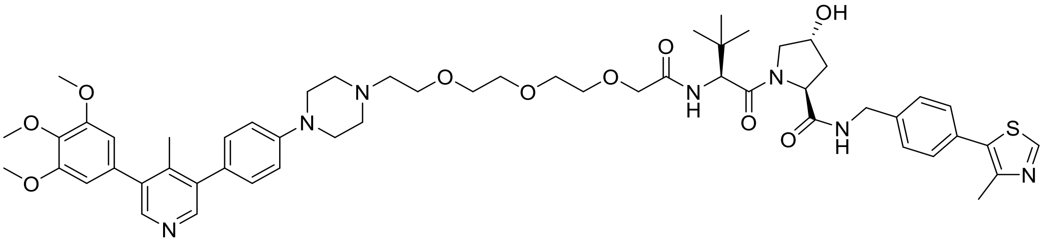

^{1}H NMR (400 MHz, d_6-DMSO)

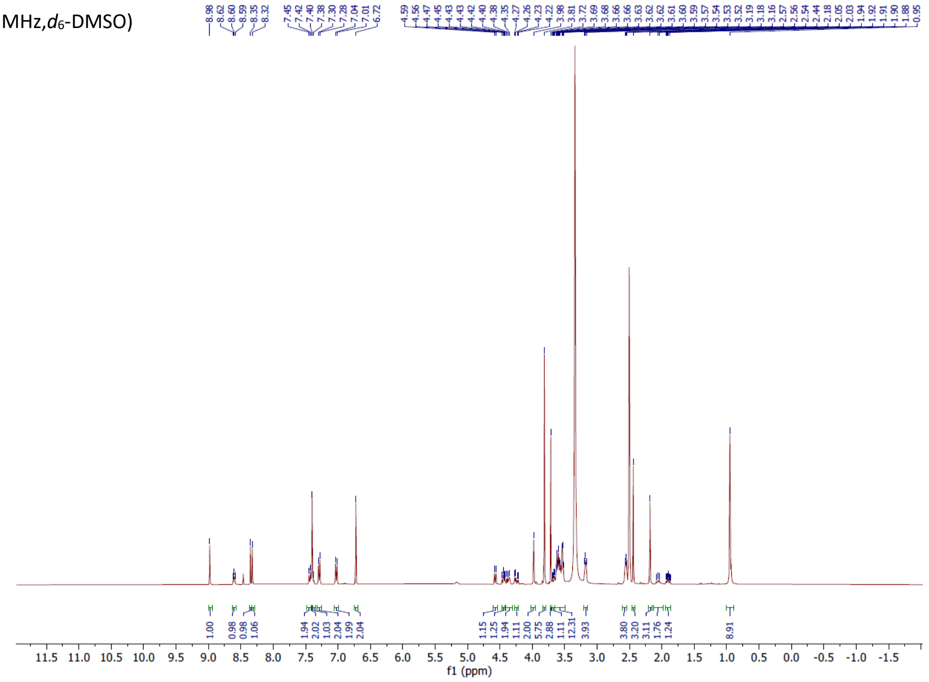

^{13}C NMR (101 MHz, d_6-DMSO)

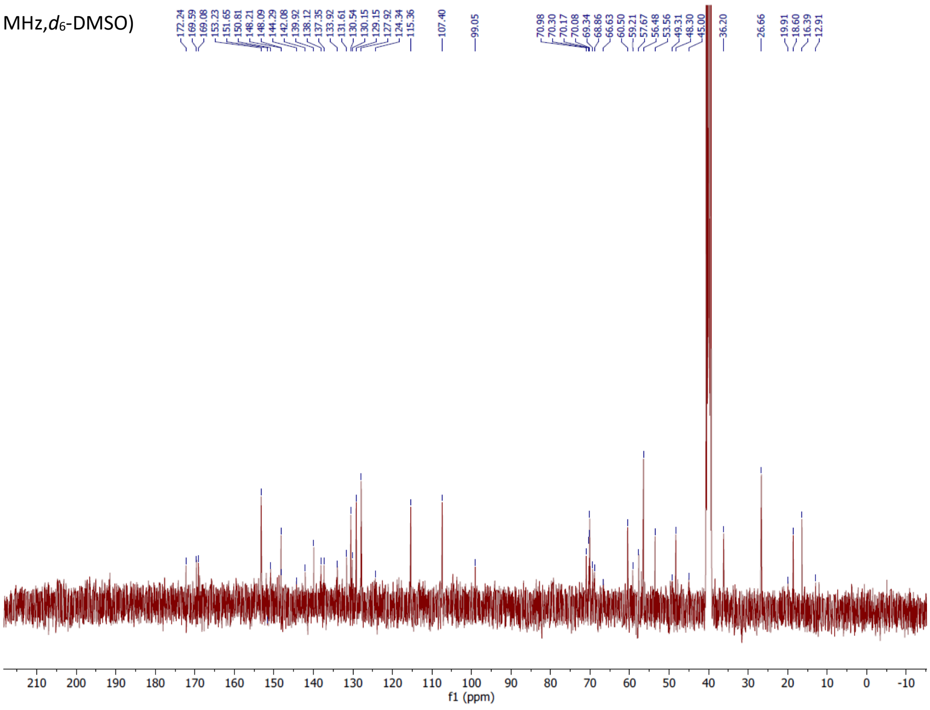

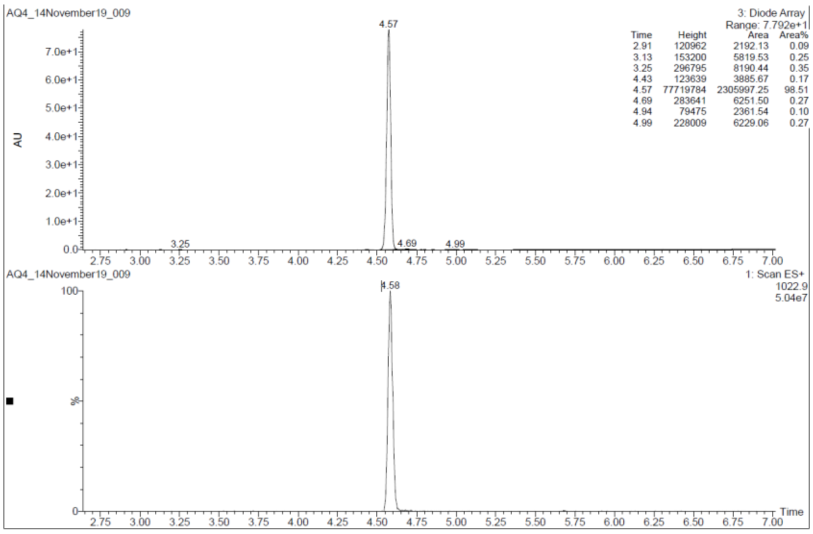
AQ4_14November19_009
3: Diode Array
Range: 7.792e+1
Time Height Area Area%
2.91 120962 2192.13 0.09
3.13 153200 5819.53 0.25
3.25 296795 8190.44 0.35
4.43 123639 3885.67 0.17
4.57 77719784 2305997.25 98.51
4.69 283641 6251.50 0.27
4.94 79475 2361.54 0.10
4.99 228009 6229.06 0.27
AU
4.57
3.25
4.69
4.99
AQ4_14November19_009
1: Scan ES+
1022.9
5.04e7
4.58
%
Time

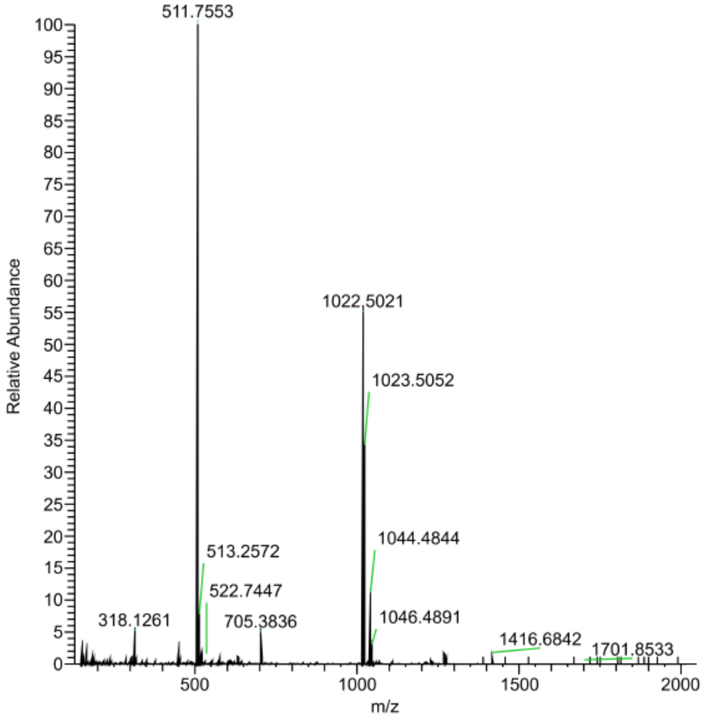
511.7553
1022.5021
1023.5052
1044.4844
513.2572
522.7447
318.1261
705.3836
1046.4891
1416.6842
1701.8533
Relative Abundance
m/z

2-(2,6-Dioxopiperidin-3-yl)-5-(4-((4-(4-(4-methyl-5-(3,4,5-trimethoxyphenyl)pyridin-3-yl)phenyl)piperazin-1-yl)methyl)piperidin-1-yl)isoindoline-1,3-dione (**13**)

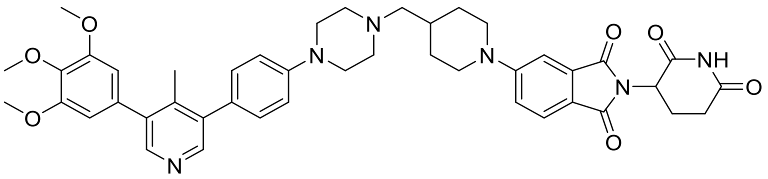

^{1}H NMR (400 MHz, $CDCl_3$)

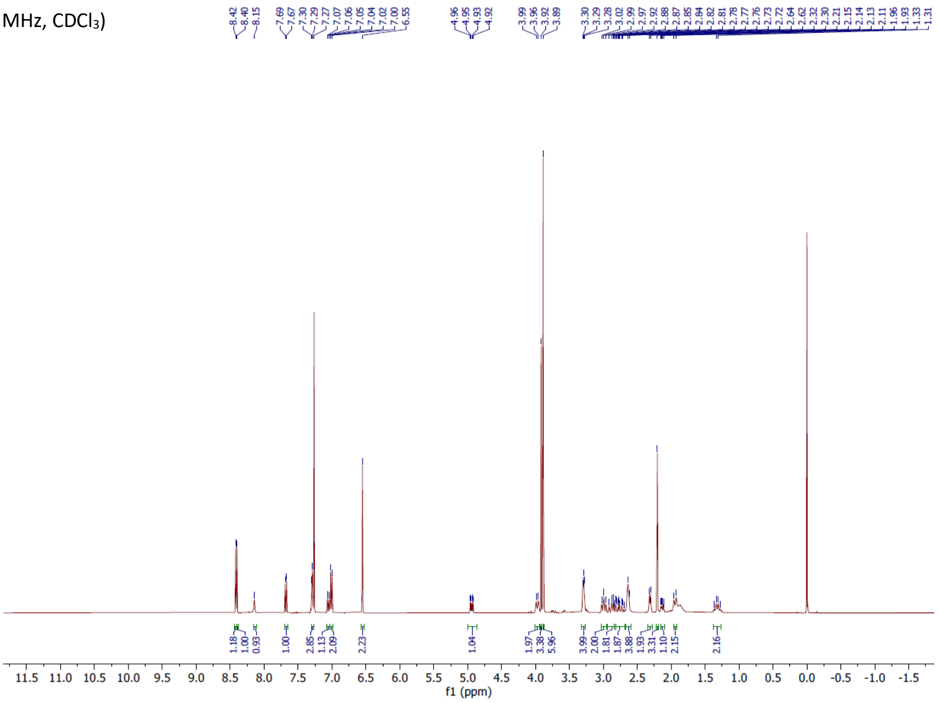

^{13}C NMR (101 MHz, $CDCl_3$)

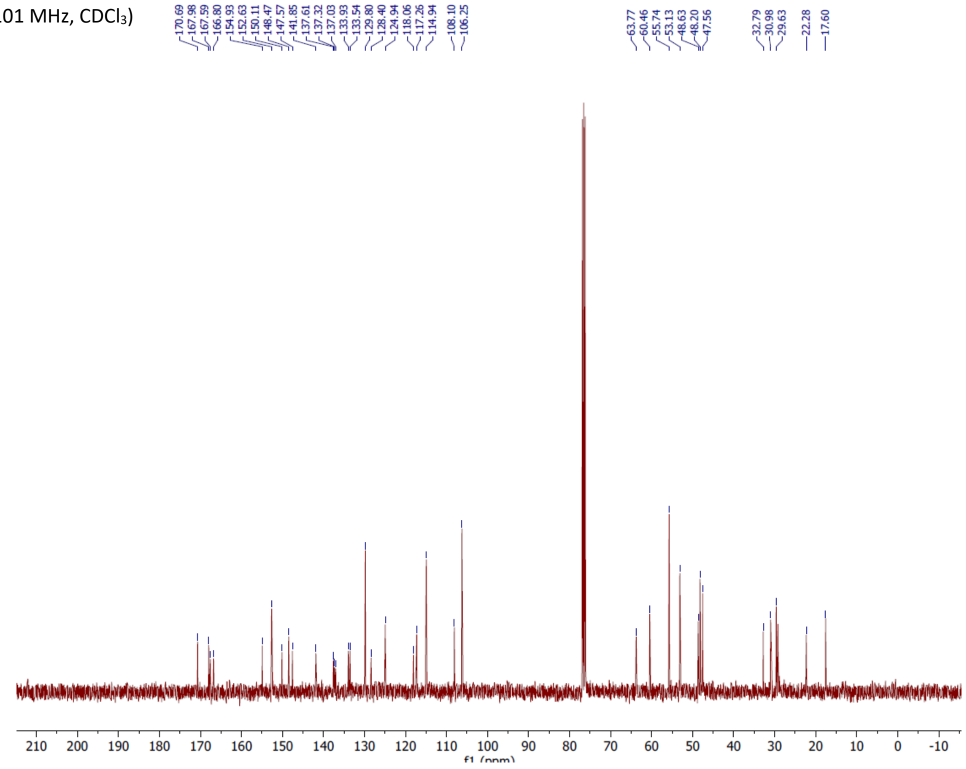

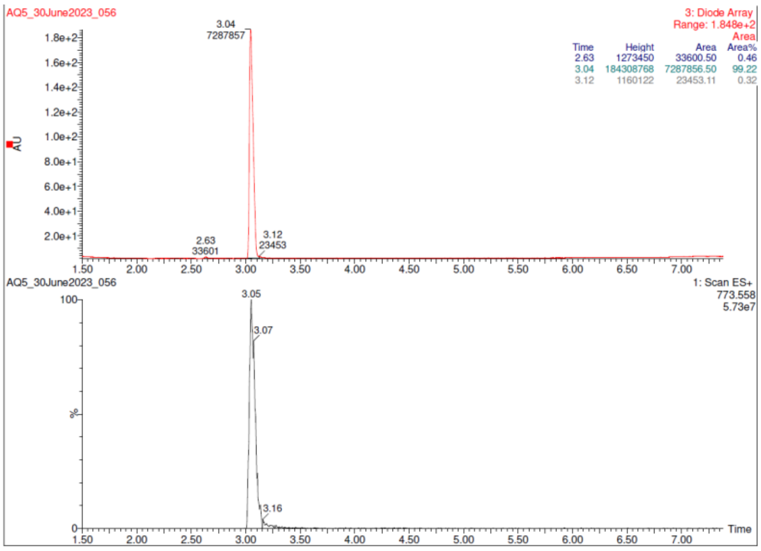
AQ5_30June2023_056
3: Diode Array
Range: 1.848e+2
Time Height Area Area%
2.63 1273450 33600.50 0.46
3.04 184308768 7287856.50 99.22
3.12 1160122 23453.11 0.32
3.04
7287857
2.63
33601
3.12
23453
AU
AQ5_30June2023_056
1: Scan ES+
773.558
5.73e7
3.05
3.07
3.16
%
Time

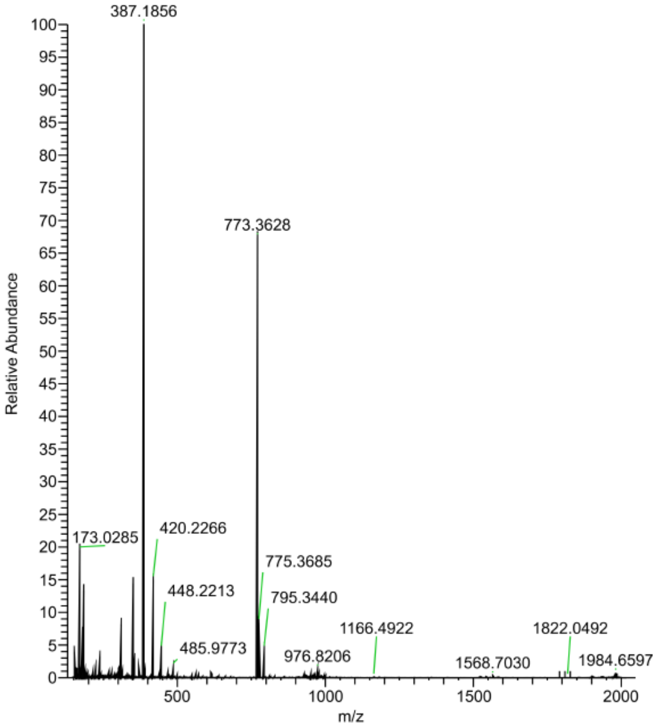
387.1856
773.3628
173.0285
420.2266
775.3685
448.2213
795.3440
1166.4922
1822.0492
485.9773
976.8206
1568.7030
1984.6597
Relative Abundance
m/z

2-(2,6-Dioxopiperidin-3-yl)-5-(4-(4-(4-(4-methyl-5-(3,4,5-trimethoxyphenyl)pyridin-3-yl)phenyl)piperazine-1-carbonyl)piperidin-1-yl)isoindoline-1,3-dione (**14**)

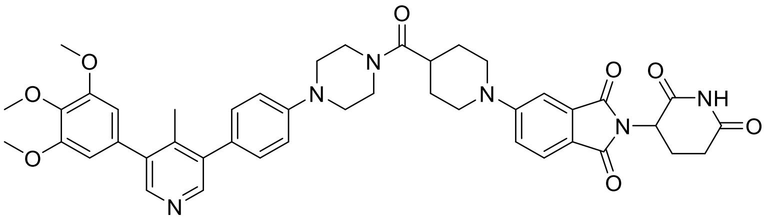

^{1}H NMR (400 MHz, $CDCl_3$)

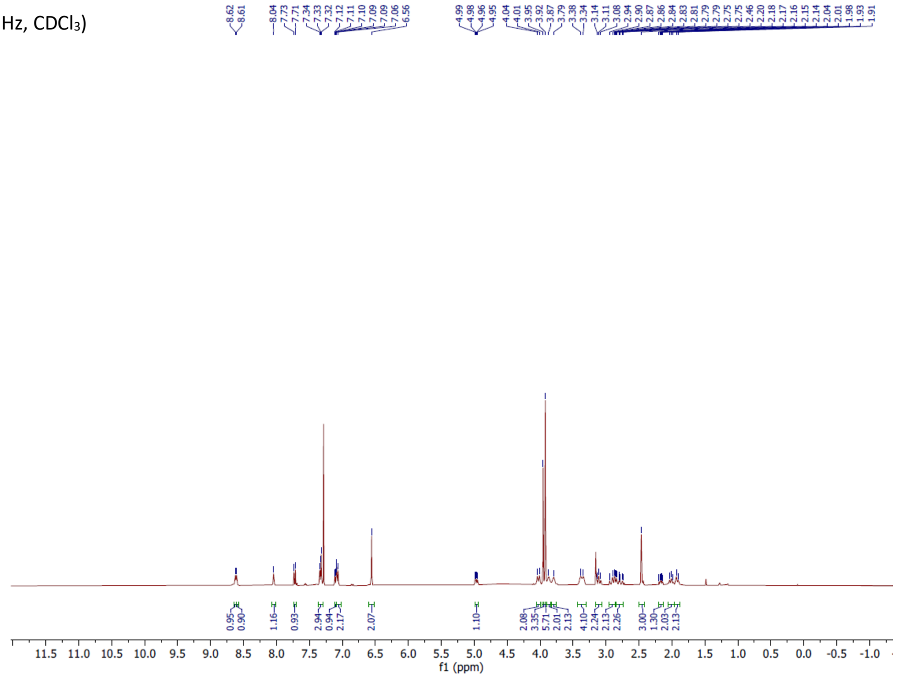

^{13}C NMR (101 MHz,$CDCl_3$)

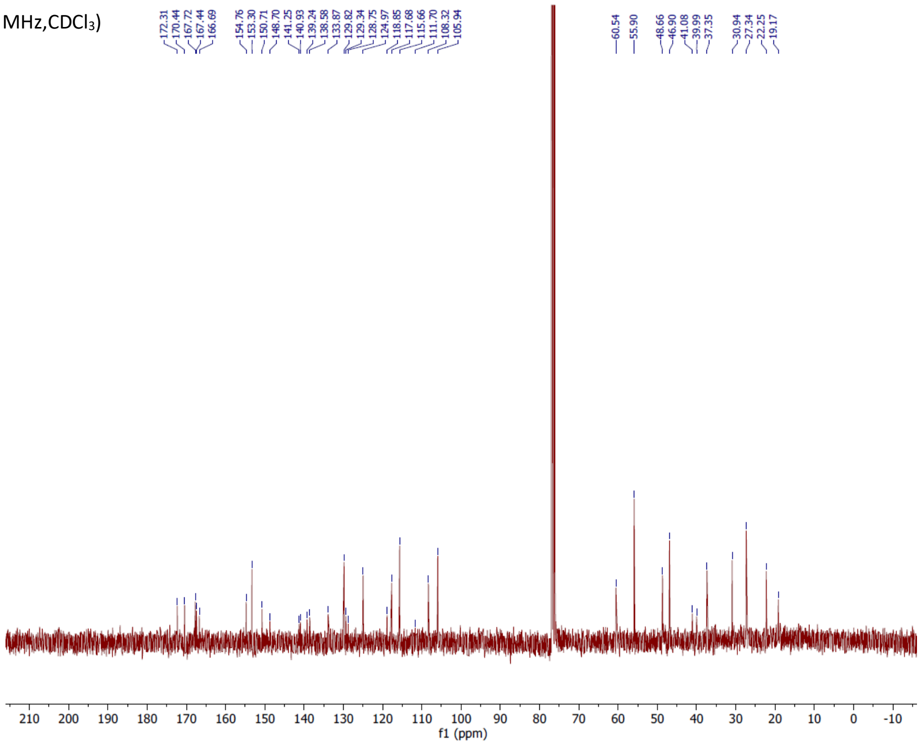

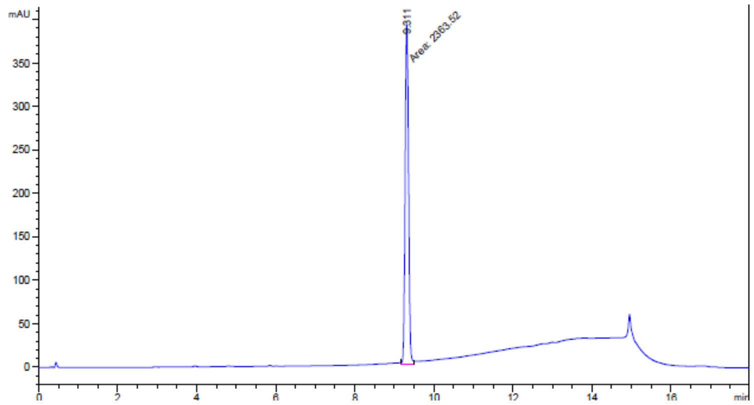

Signal 1: VWD1 A, Wavelength=250 nm

Peak #	RetTime [min]	Type	Width [min]	Area [mAU*s]	Height [mAU]	Area %
1	9.311	MM	0.1006	2363.52271	391.56064	100.0000
Totals :				2363.52271	391.56064	

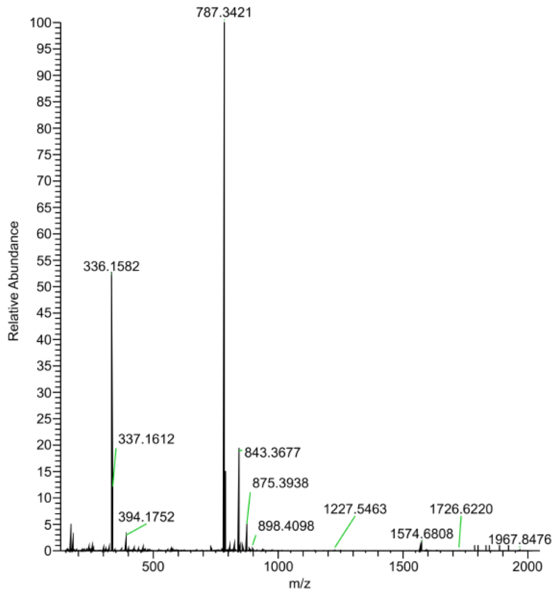

2-(2,6-Dioxopiperidin-3-yl)-5-(4-fluoro-4-(4-(4-(4-methyl-5-(3,4,5-trimethoxyphenyl)pyridin-3-yl)phenyl)piperazine-1-carbonyl)piperidin-1-yl)isoindoline-1,3-dione (**15**)

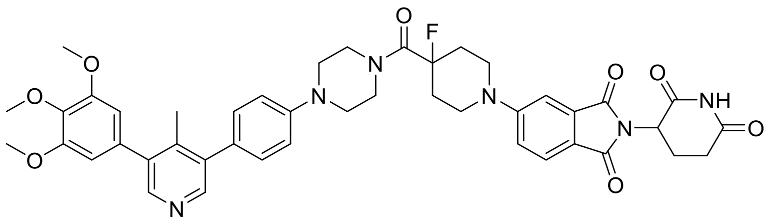

^{1}H NMR (400 MHz, CDCl$_3$)

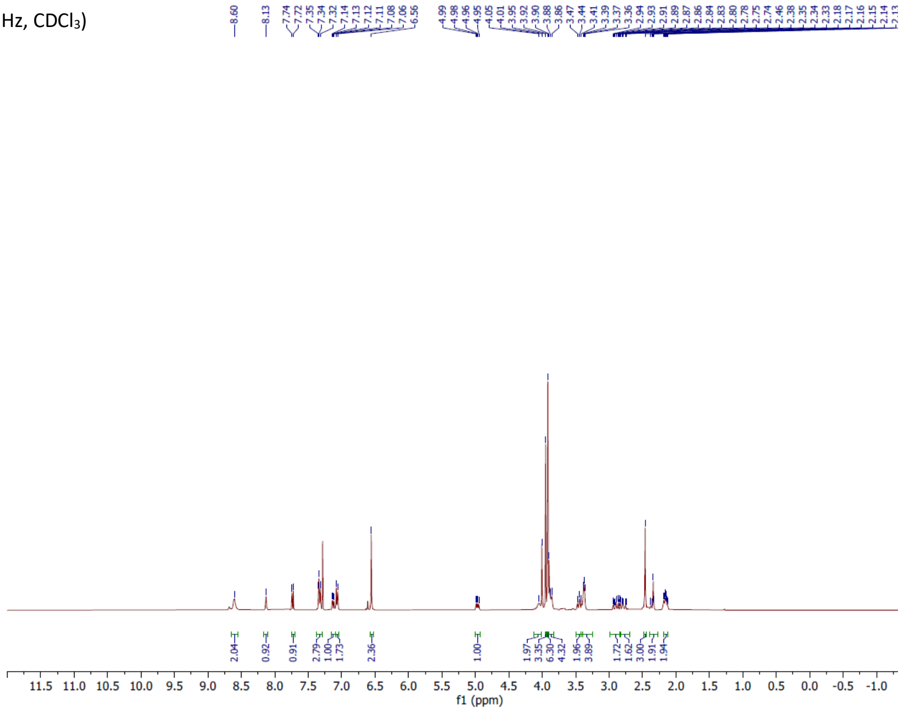

^{13}C NMR (101 MHz, CDCl$_3$)

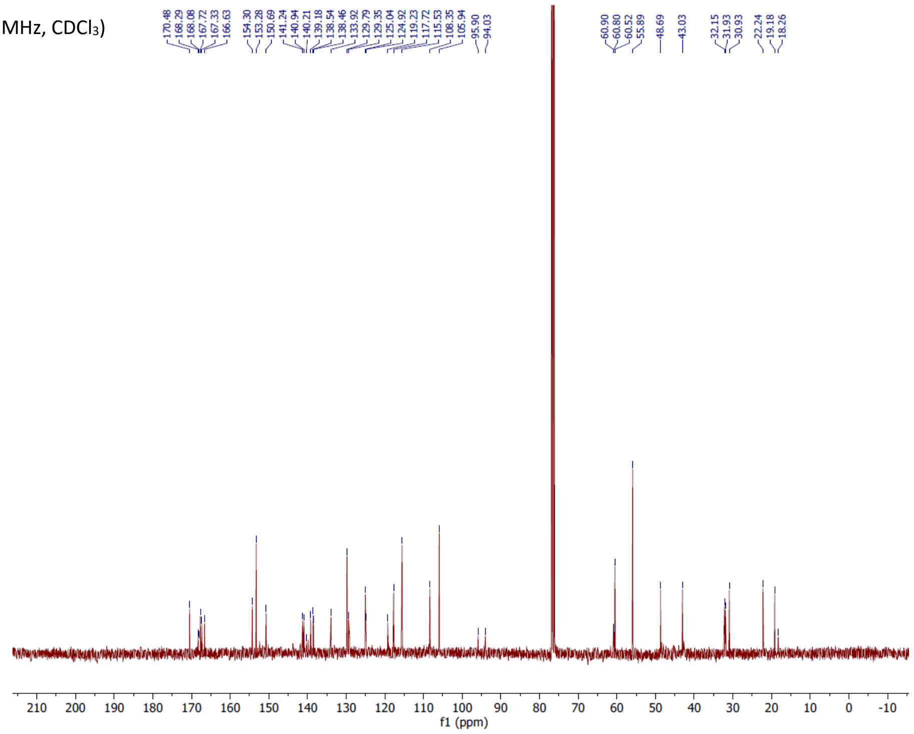

^{19}F NMR (376 MHz, $CDCl_3$)

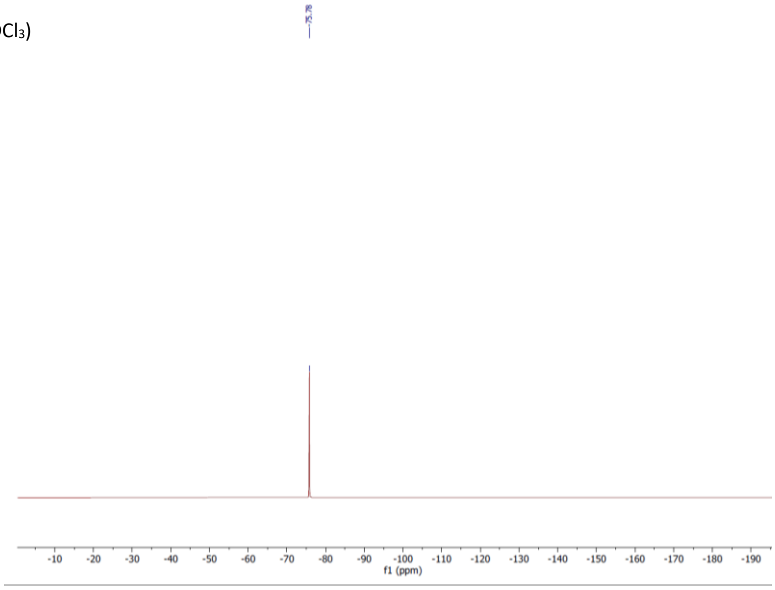

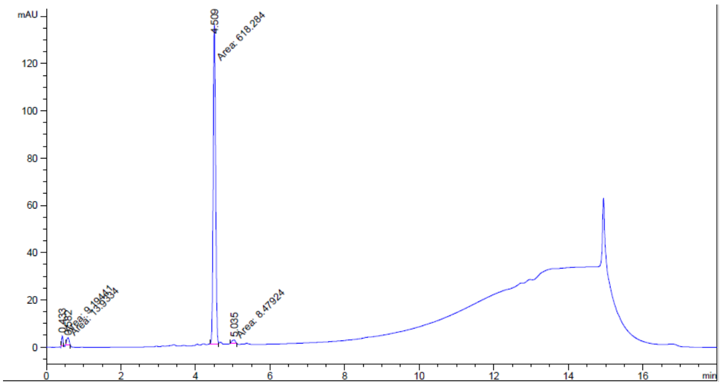


```
Signal 1: VWD1 A, Wavelength=250 nm
```

Peak #	RetTime [min]	Type	Width [min]	Area [mAU*s]	Height [mAU]	Area %
1	0.433	MM	0.0366	9.19441	4.18751	1.4148
2	0.582	MM	0.0743	13.93343	3.12539	2.1440
3	4.509	MM	0.0761	618.28406	135.41283	95.1366
4	5.035	PM	0.1024	8.47924	1.38012	1.3047
Totals :				649.89113	144.10584	

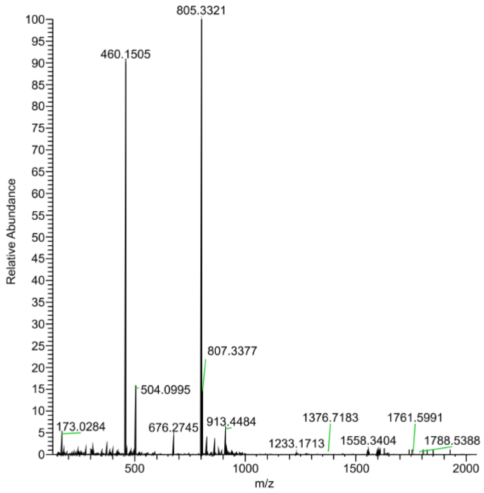

2-(2,6-Dioxopiperidin-3-yl)-5-(3-(4-(4-(4-methyl-5-(3,4,5-trimethoxyphenyl)pyridin-3-yl)phenyl)piperazine-1-carbonyl)azetidin-1-yl)isoindoline-1,3-dione (**16**)

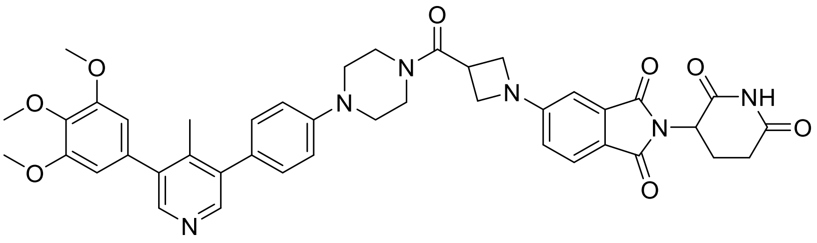

^{1}H NMR (400 MHz, $CDCl_3$)

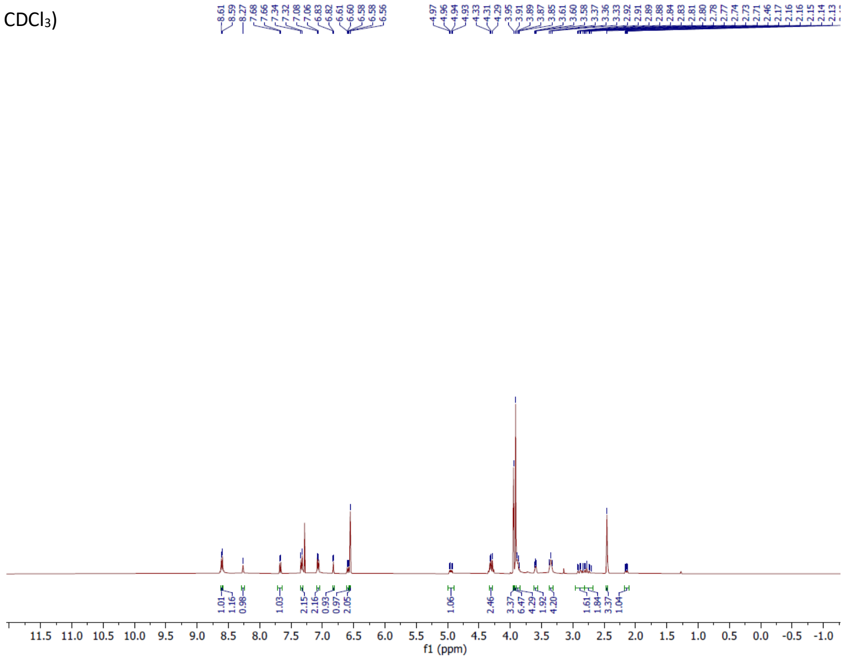

^{13}C NMR (101 MHz, $CDCl_3$)

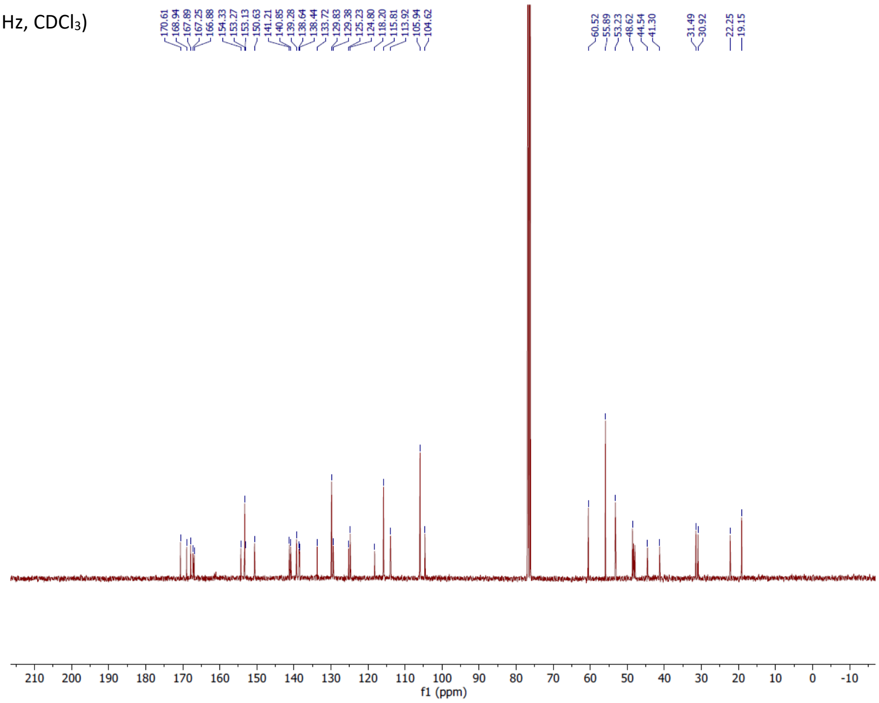

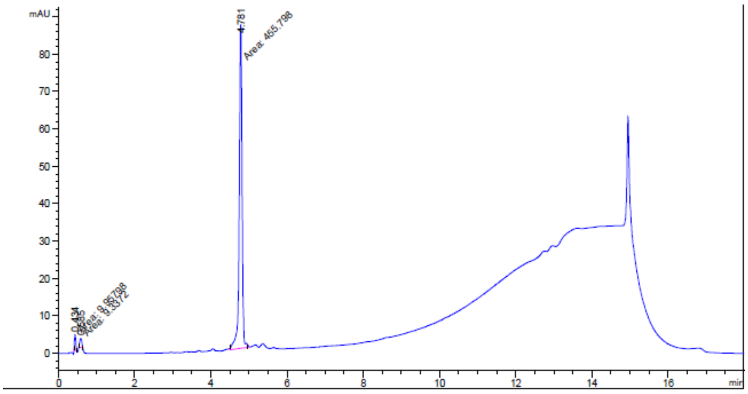

Signal 1: VWD1 A, Wavelength=250 nm

Peak #	RetTime [min]	Type	Width [min]	Area [mAU*s]	Height [mAU]	Area %
1	0.434	MM	0.0381	9.95798	4.35352	2.0960
2	0.585	MM	0.0651	9.33720	2.38901	1.9653
3	4.781	MM	0.0875	455.79791	86.82591	95.9387

Totals : 475.09309 93.56844

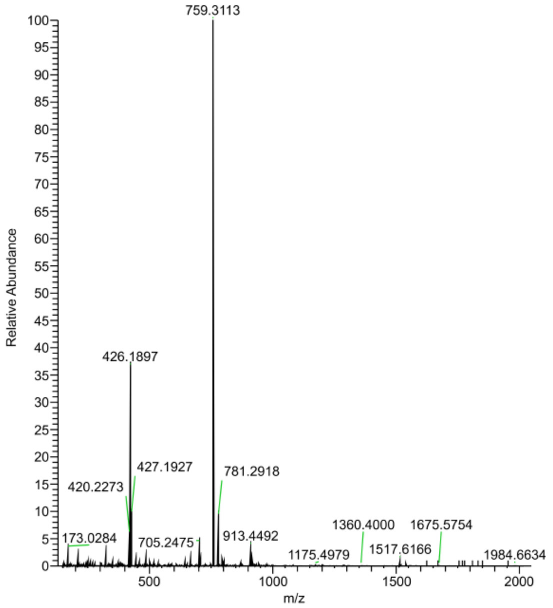

1-(2-Methoxy-5-(4-(4-(4-methyl-5-(3,4,5-trimethoxyphenyl)pyridin-3-yl)phenyl)piperazine-1-carbonyl)phenyl)dihydropyrimidine-2,4(1H,3H)-dione (**17**)

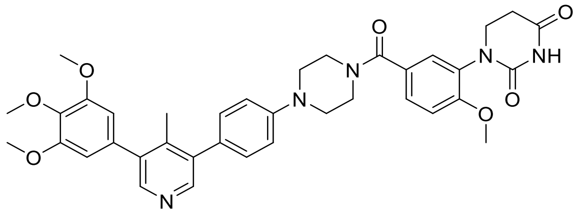

1H NMR (400 MHz, $CDCl_3$)

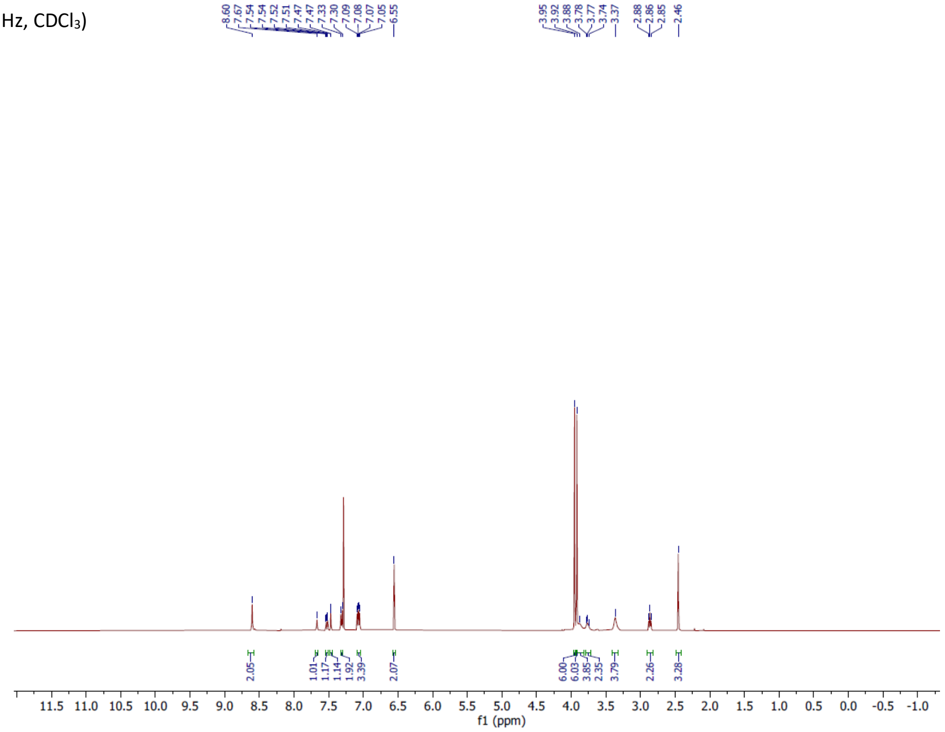

13C NMR (101 MHz, $CDCl_3$)

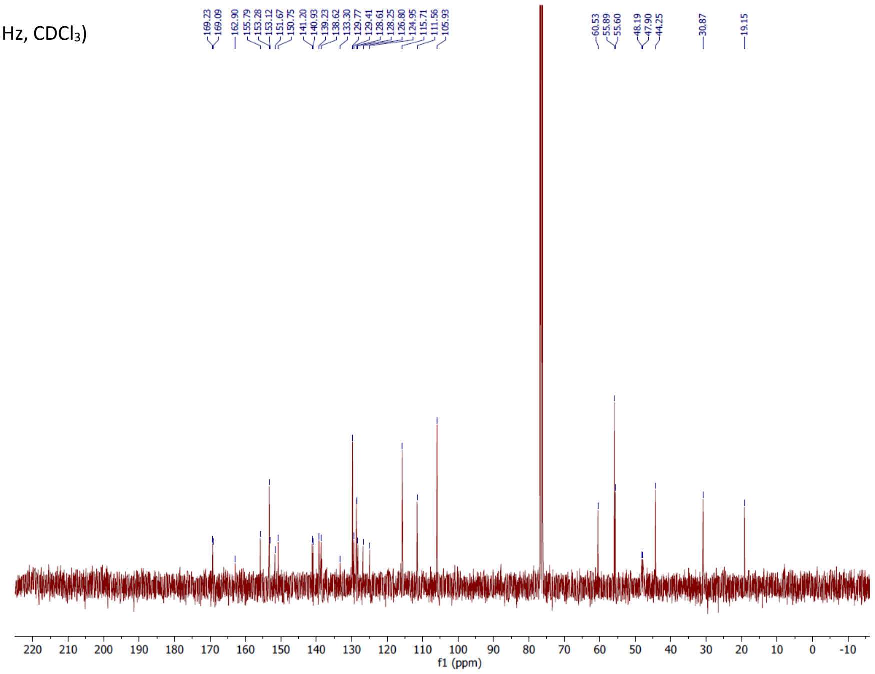

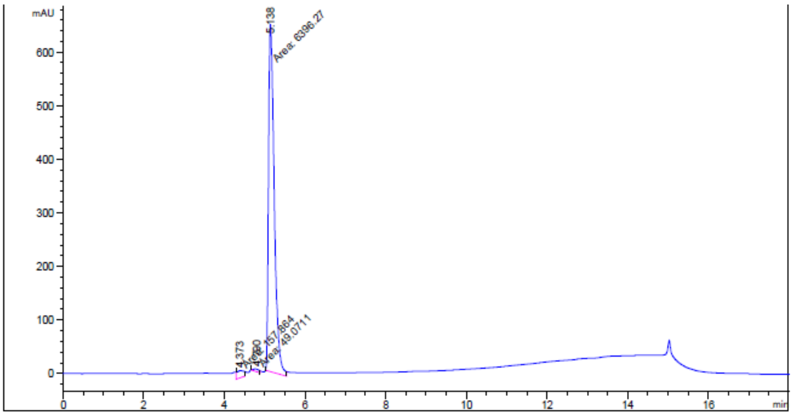

Signal 1: VWD1 A, Wavelength=250 nm

Peak #	RetTime [min]	Type	Width [min]	Area [mAU*s]	Height [mAU]	Area %
1	4.373	MM	0.1896	157.86430	13.87493	2.3907
2	4.790	PM	0.1730	49.07109	4.72843	0.7431
3	5.138	PM	0.1643	6396.26563	648.69495	96.8661

Totals : 6603.20102 667.29830

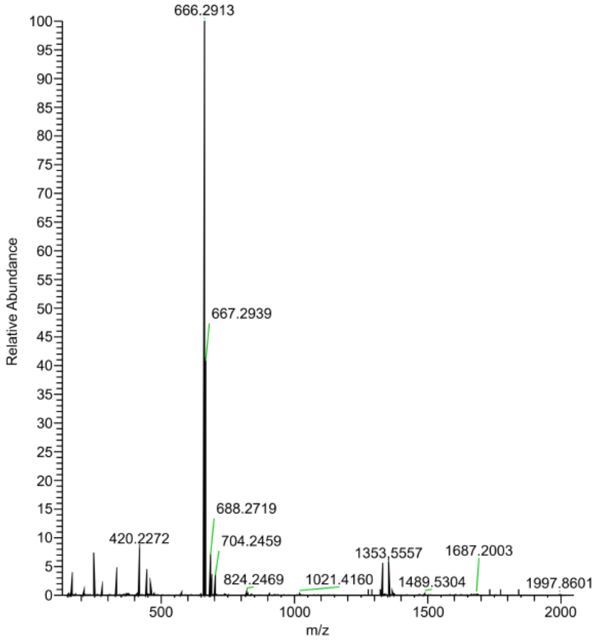

1-(2-Methoxy-5-(4-((4-(4-(4-methyl-5-(3,4,5-trimethoxyphenyl)pyridin-3-yl)phenyl)piperazin-1-yl)methyl)piperidine-1-carbonyl)phenyl)dihydropyrimidine-2,4(1H,3H)-dione (**18**)

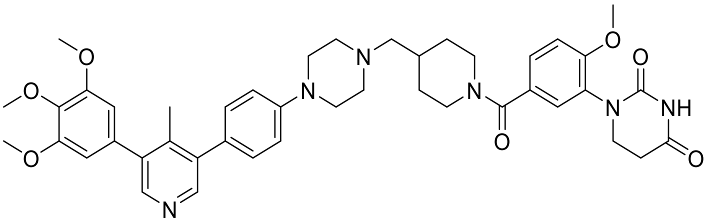

^{1}H NMR (400 MHz, $CDCl_3$)

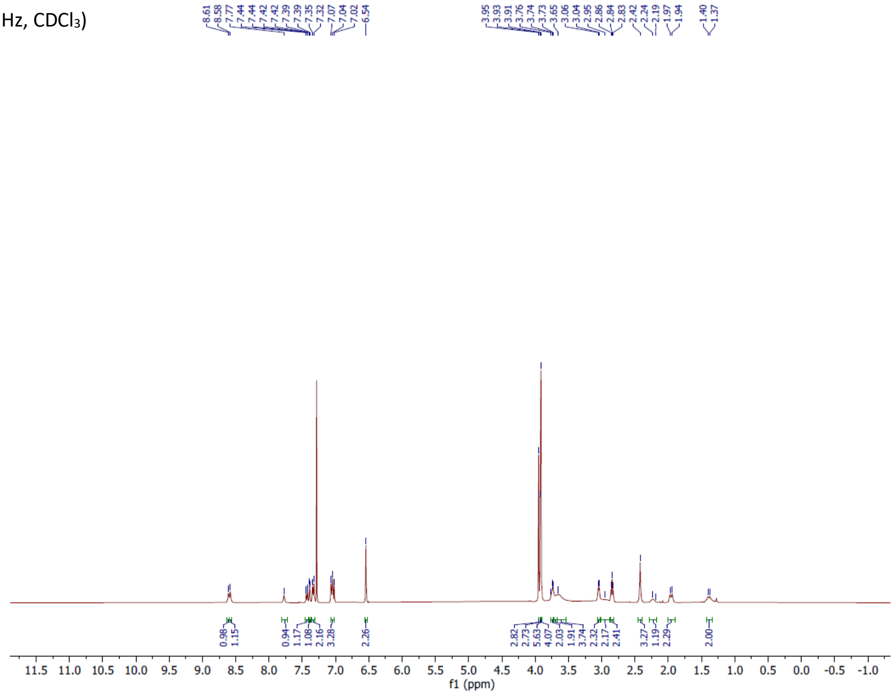

^{13}C NMR (101 MHz, $CDCl_3$)

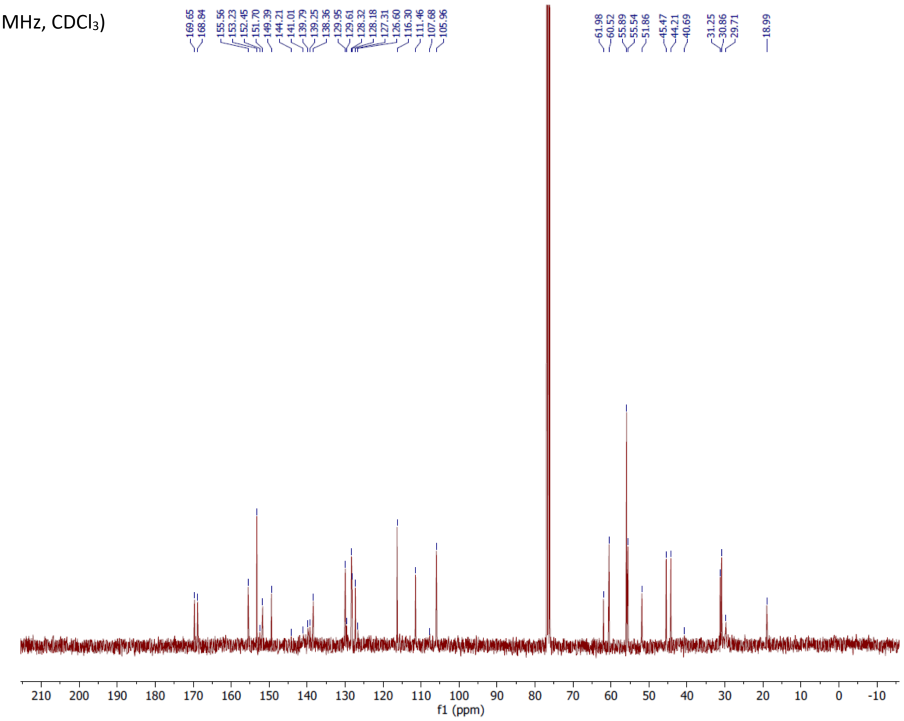

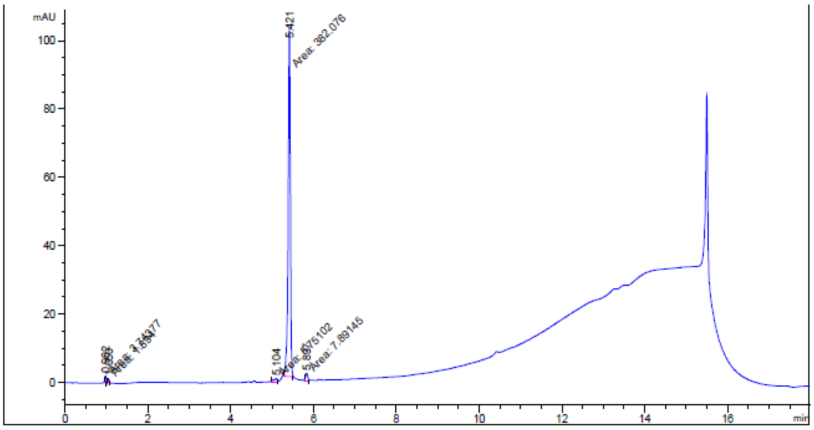

Signal 1: VWD1 A, Wavelength=250 nm

Peak #	RetTime [min]	Type	Width [min]	Area [mAU*s]	Height [mAU]	Area %
1	0.982	MM	0.0309	3.74377	2.02109	0.9328
2	1.050	MM	0.0284	1.89400	1.11116	0.4719
3	5.104	MM	0.0977	5.75102	9.80716e-1	1.4329
4	5.421	MM	0.0623	382.07553	102.14725	95.1962
5	5.837	MM	0.0599	7.89145	2.19395	1.9662

Totals : 401.35577 108.45417

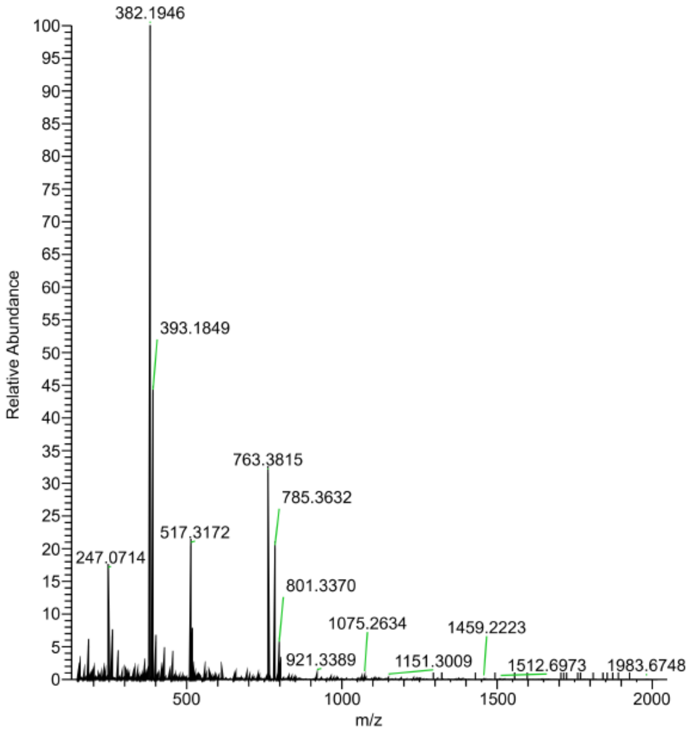

2-(1-Methyl-2,6-dioxopiperidin-3-yl)-5-(4-((4-(4-(4-methyl-5-(3,4,5-trimethoxyphenyl)pyridin-3-yl)phenyl)piperazin-1-yl)methyl)piperidin-1-yl)isoindoline-1,3-dione (**19**)

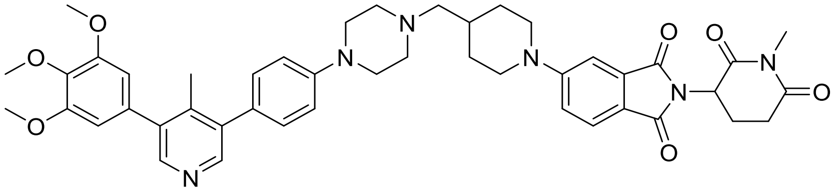

^{1}H NMR (400 MHz, $CDCl_3$)

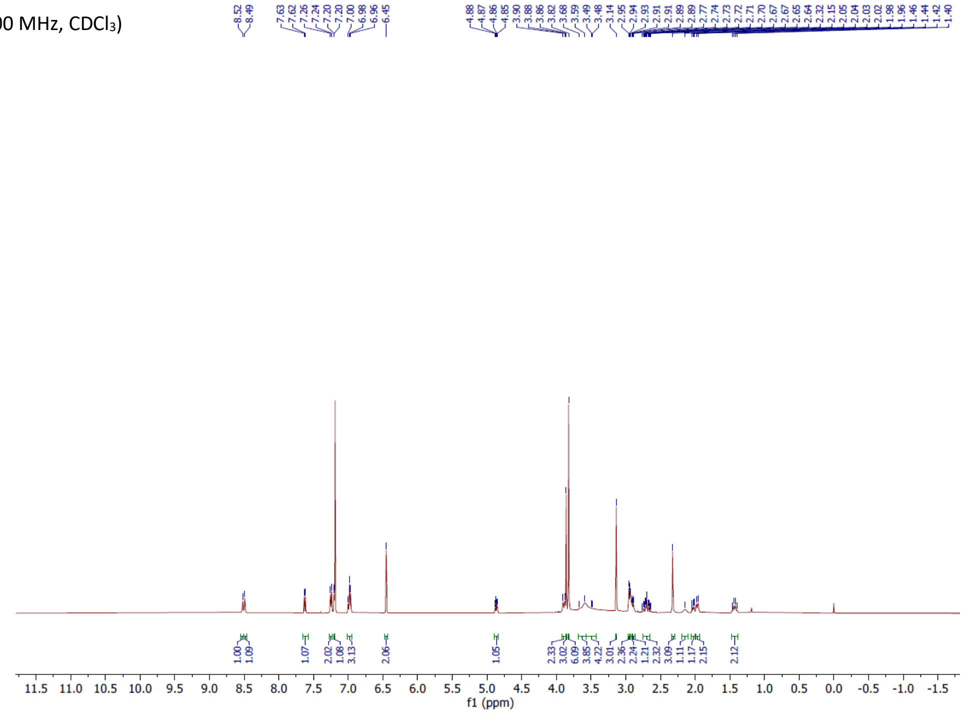

^{13}C NMR (101 MHz, $CDCl_3$)

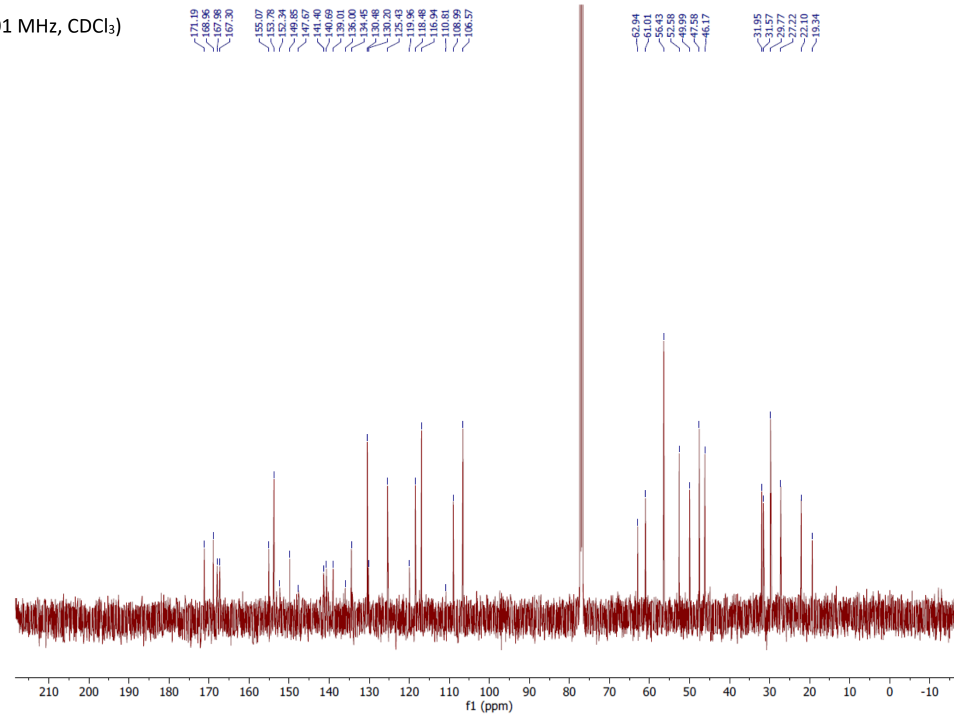

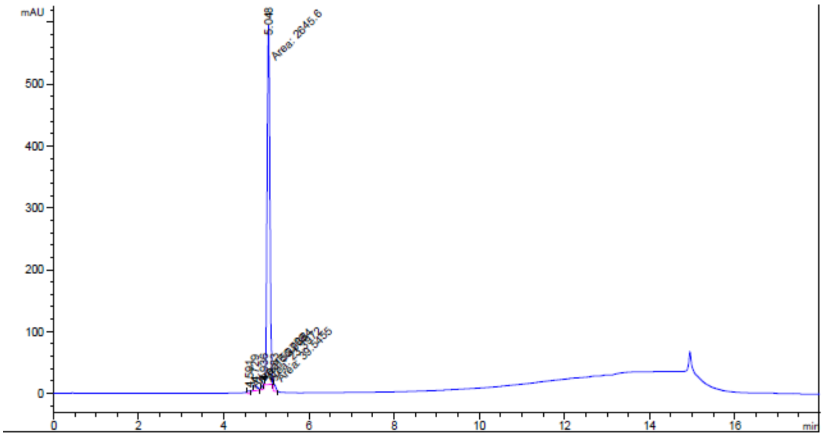

Peak #	RetTime [min]	Type	Width [min]	Area [mAU*s]	Height [mAU]	Area %
1	4.591	MM T	0.0645	16.97081	4.38645	0.6112
2	4.729	MM T	0.1054	50.98837	8.06480	1.8364
3	4.936	MM T	0.0539	23.39720	7.23910	0.8427
4	5.048	MM T	0.0757	2645.60498	582.41638	95.2854
5	5.183	MM T	0.0615	39.54554	10.71070	1.4243

Totals : 2776.50690 612.81742

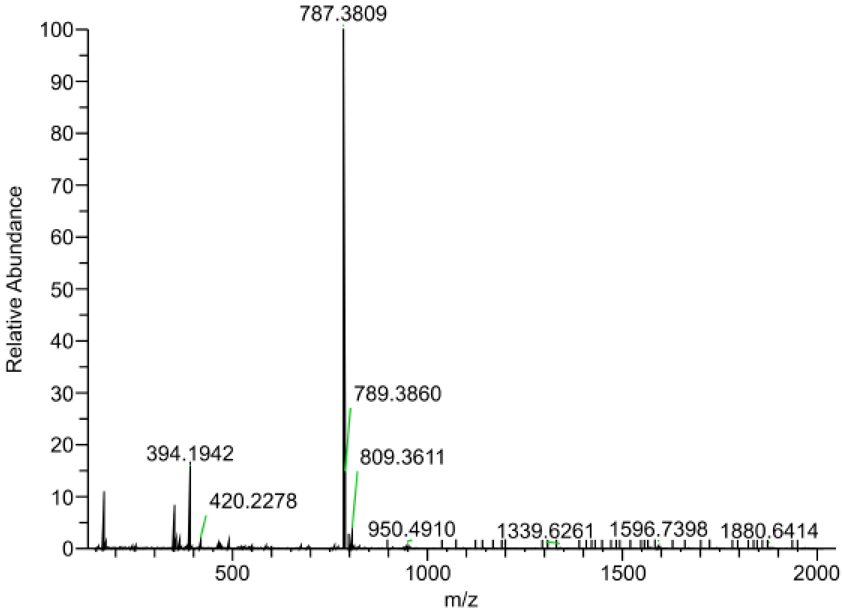

2-(2,6-Dioxopiperidin-3-yl)-5-(4-((4-(4-(5-(4-fluoro-3,5-dimethoxyphenyl)-4-methylpyridin-3-yl)phenyl)piperazin-1-yl)methyl)piperidin-1-yl)isoindoline-1,3-dione (**20**)

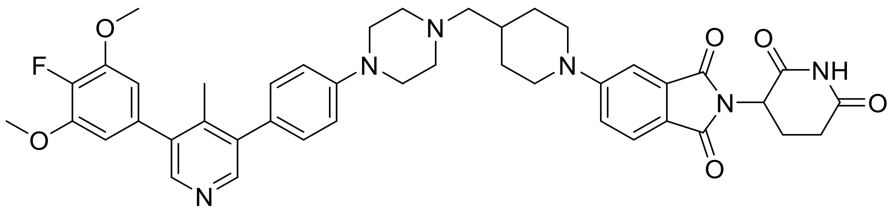

^{1}H NMR (400 MHz, $CDCl_3$)

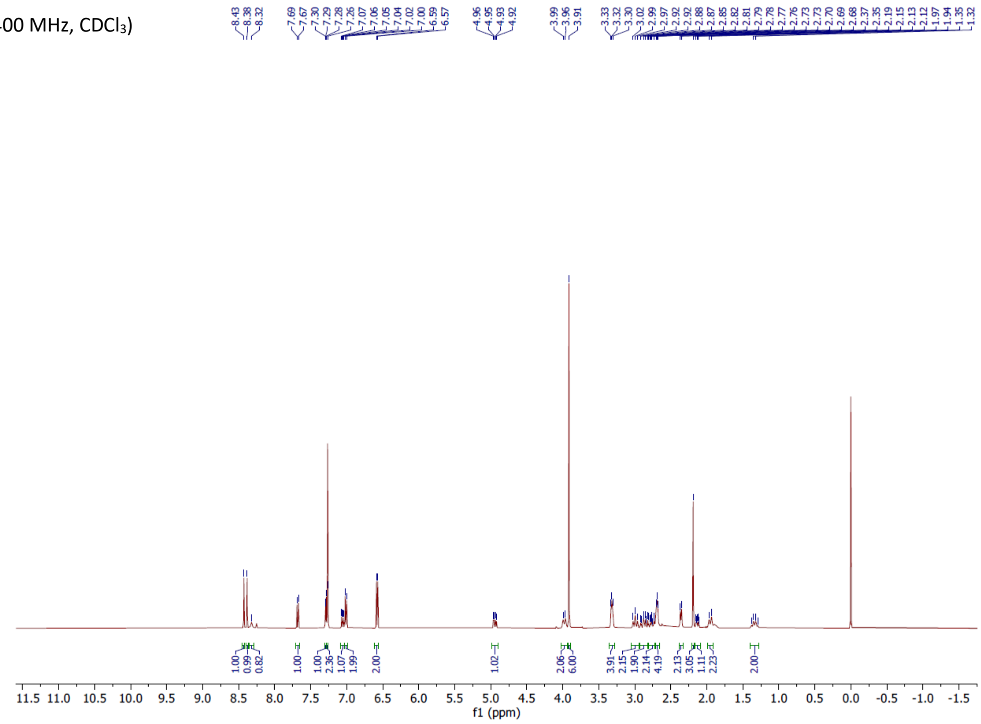

^{13}C NMR (101 MHz, $CDCl_3$)

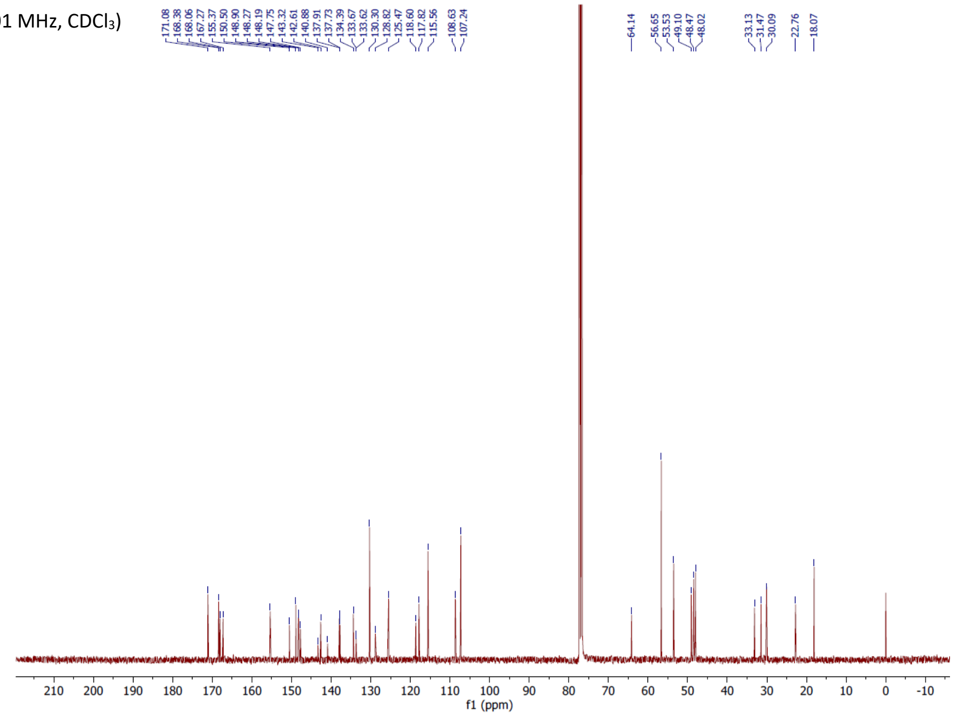

$^{19}F\{^1H\}$ NMR (376 MHz, $CDCl_3$)

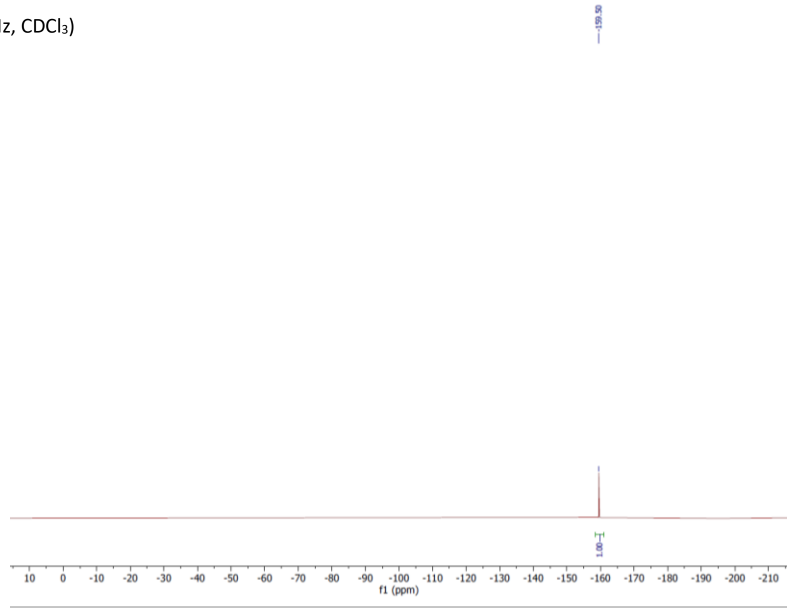

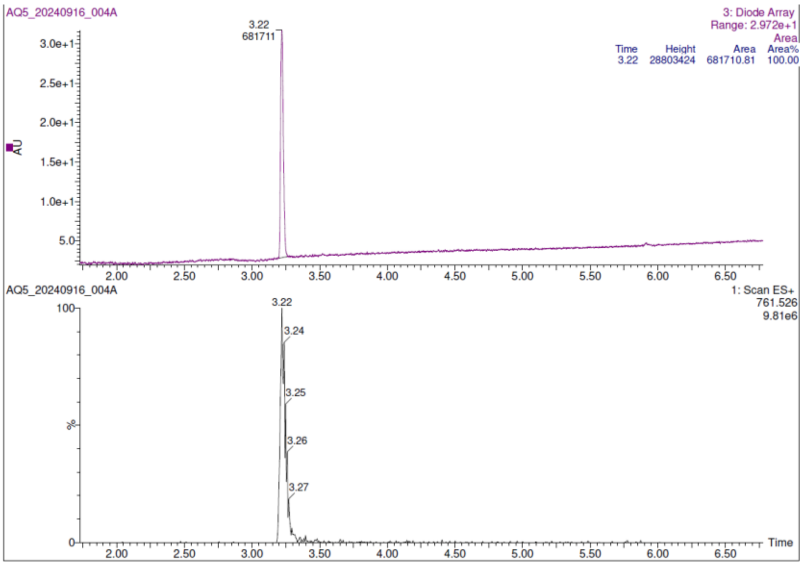

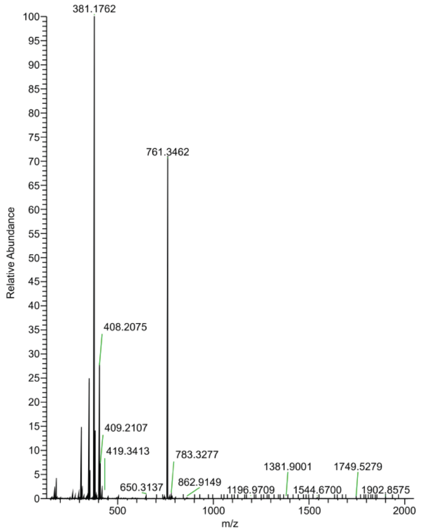

2-(2,6-Dioxopiperidin-3-yl)-5-(4-(4-(4-(5-(4-fluoro-3,5-dimethoxyphenyl)-4-methylpyridin-3-yl)phenyl)piperazine-1-carbonyl)piperidin-1-yl)isoindoline-1,3-dione (**21**)

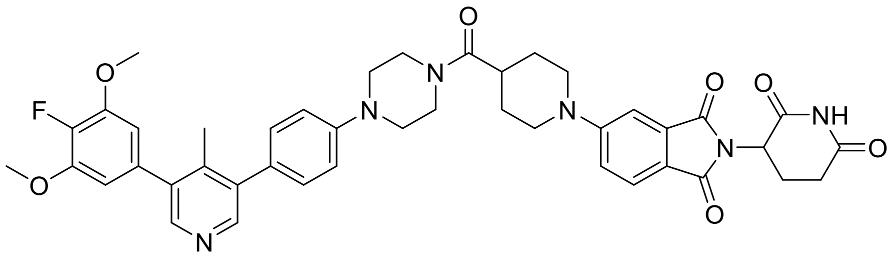

^{1}H NMR (500 MHz, $CDCl_3$)

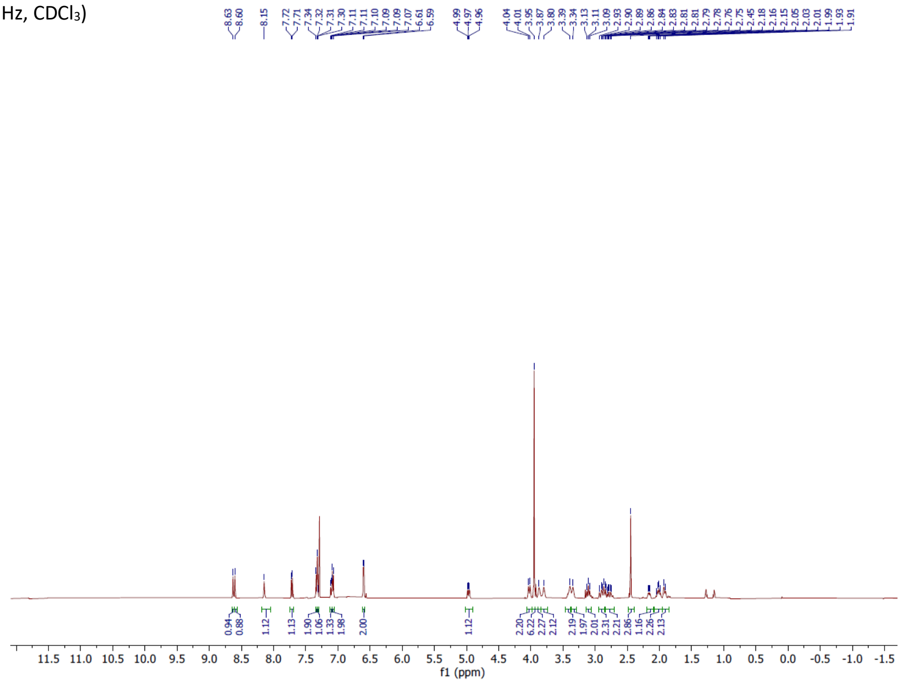

^{13}C NMR (126 MHz, $CDCl_3$)

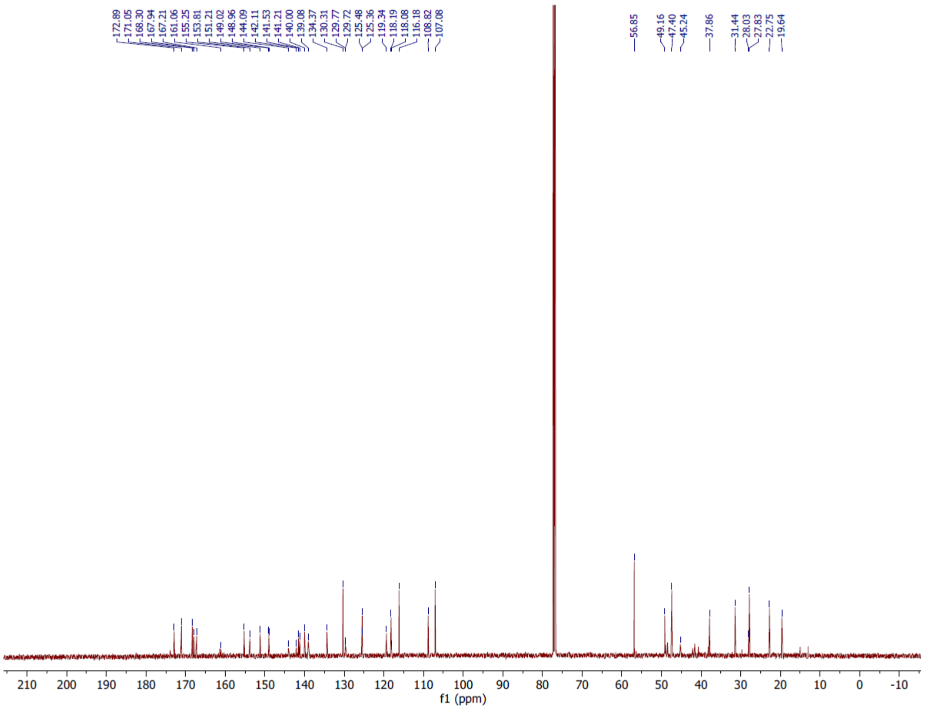

^{19}F NMR (471 MHz, $CDCl_3$)

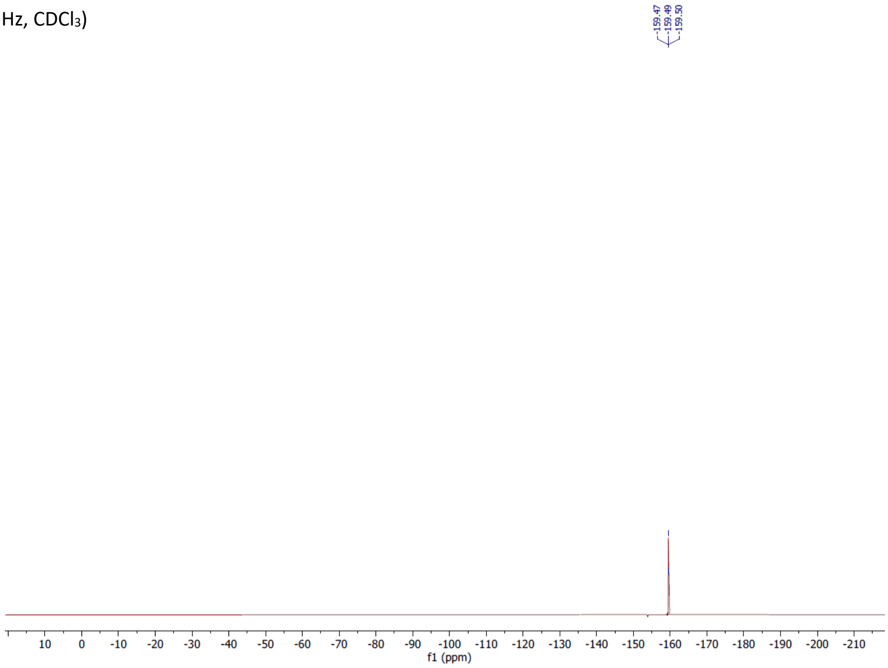

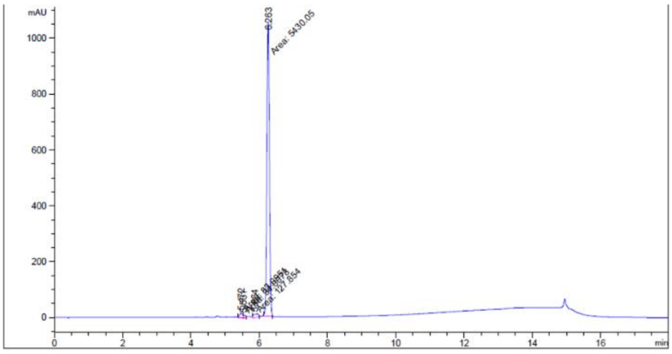

Peak #	RetTime [min]	Type	Width [min]	Area [mAU*s]	Height [mAU]	Area %
1	5.472	MM	0.0924	83.09515	14.98123	1.4642
2	5.531	MM	0.0584	34.00780	7.79105	0.5993
3	5.864	MM	0.1751	127.85358	12.17251	2.2529
4	6.263	MM	0.0857	5430.04736	1056.40625	95.6836
Totals :				5675.00390	1091.35104	

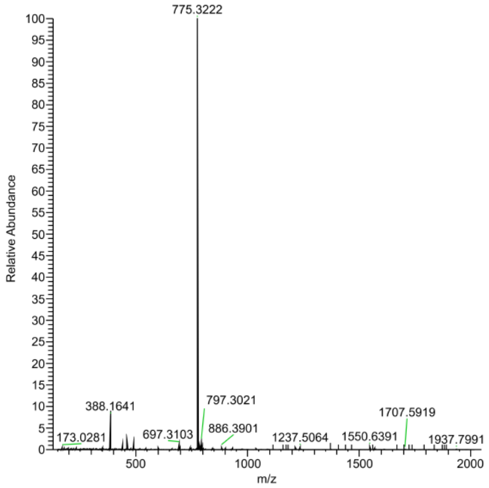

2-(2,6-Dioxopiperidin-3-yl)-5-(3-((4-(4-(5-(4-fluoro-3,5-dimethoxyphenyl)-4-methylpyridin-3-yl)phenyl)piperazin-1-yl)methyl)pyrrolidin-1-yl)isoindoline-1,3-dione (**22**)

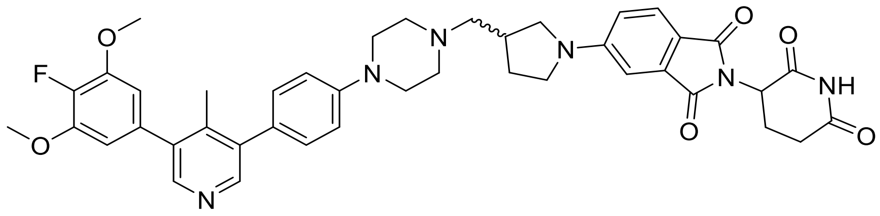

^{1}H NMR (400 MHz, CDCl$_3$)

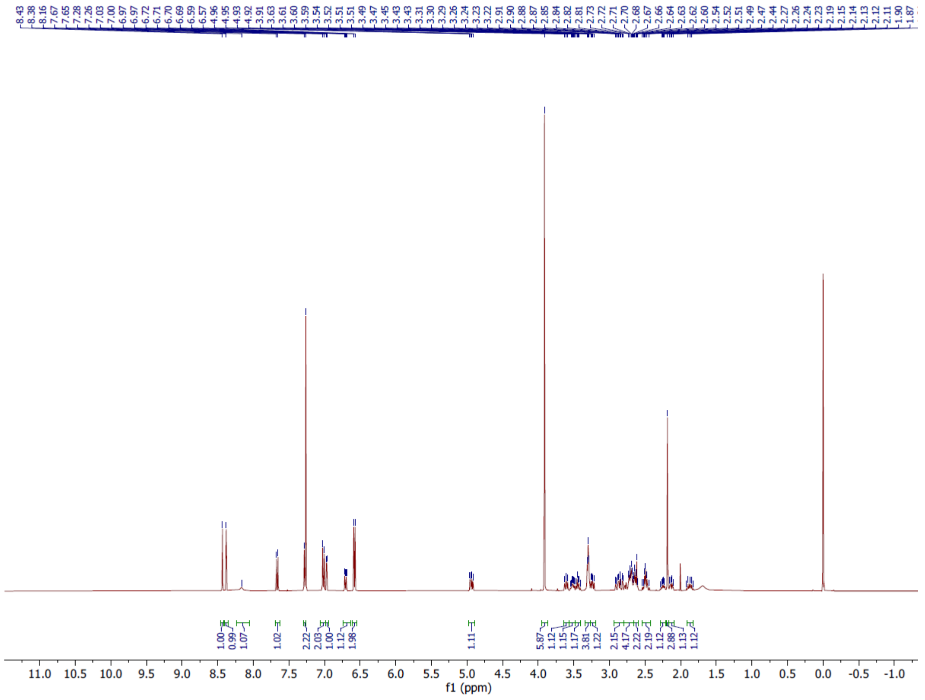

^{13}C NMR (101 MHz, CDCl$_3$)

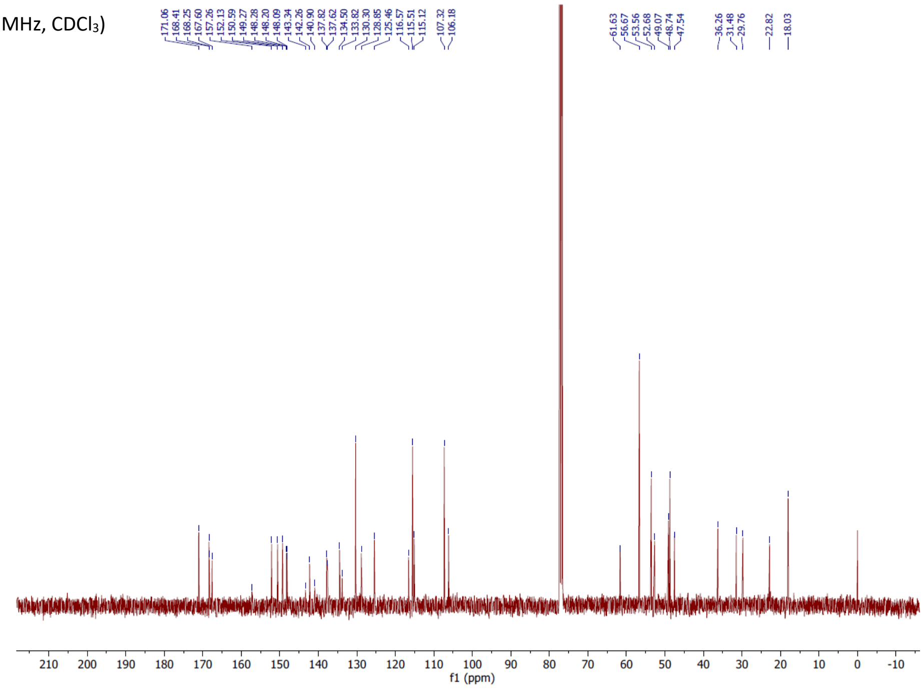

$^{19}F\{^{1}H\}$ NMR (376 MHz, $CDCl_3$)

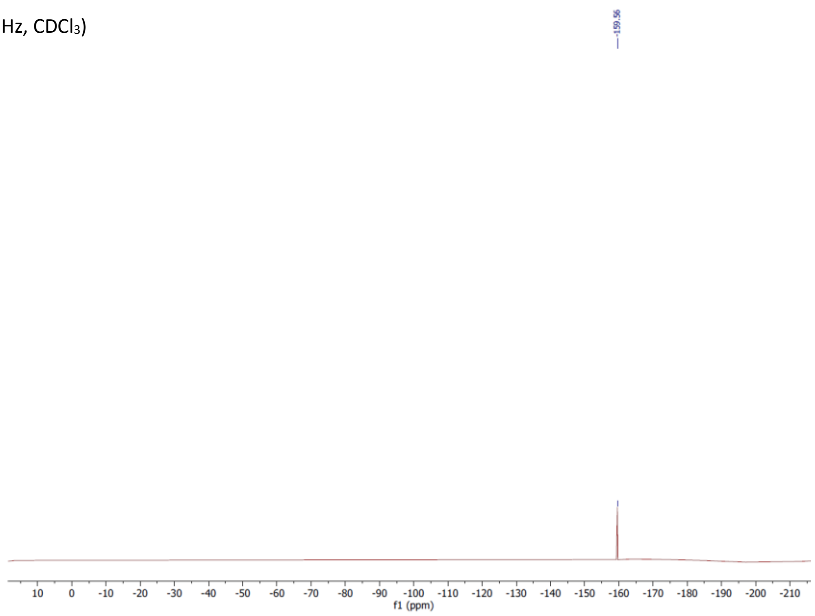

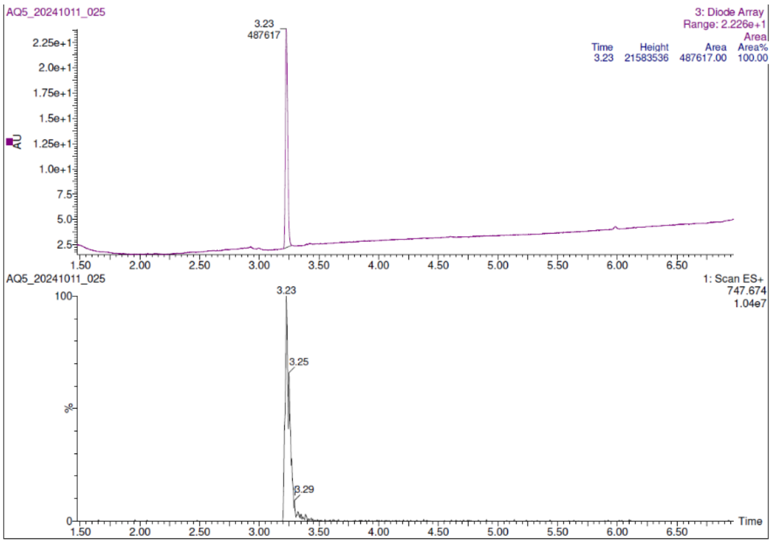

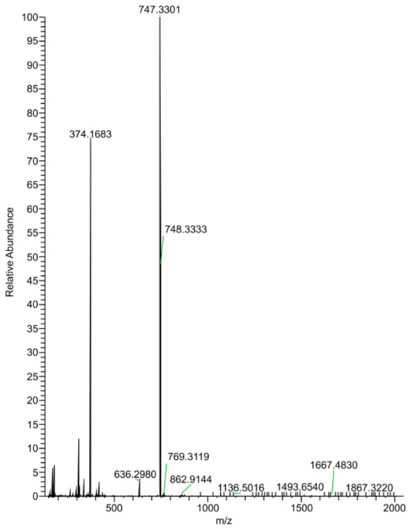

2-(2,6-Dioxopiperidin-3-yl)-5-(3-(4-(4-(5-(4-fluoro-3,5-dimethoxyphenyl)-4-methylpyridin-3-yl)phenyl)piperazine-1-carbonyl)pyrrolidin-1-yl)isoindoline-1,3-dione (**23**)

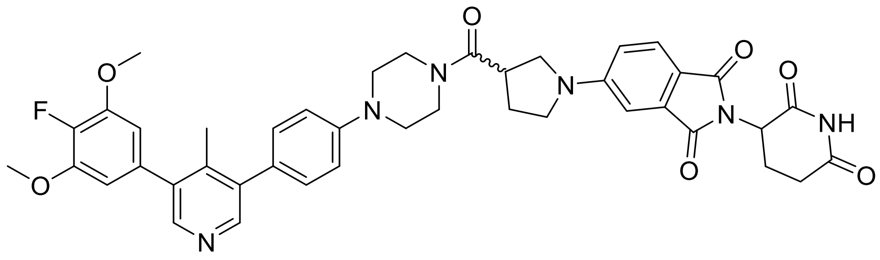

^{1}H NMR (400 MHz, $CDCl_3$)

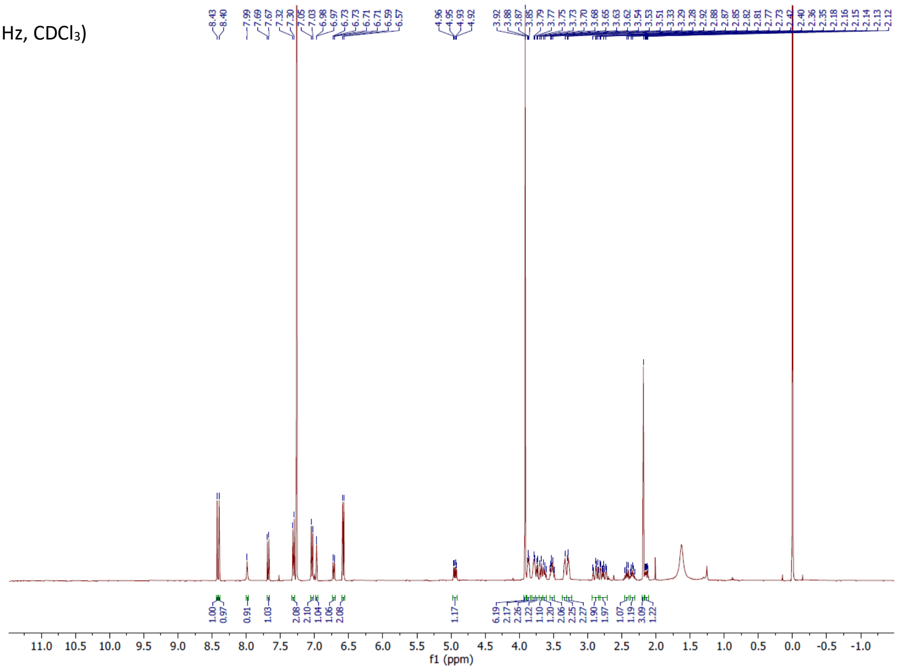

^{13}C NMR (101 MHz, $CDCl_3$)

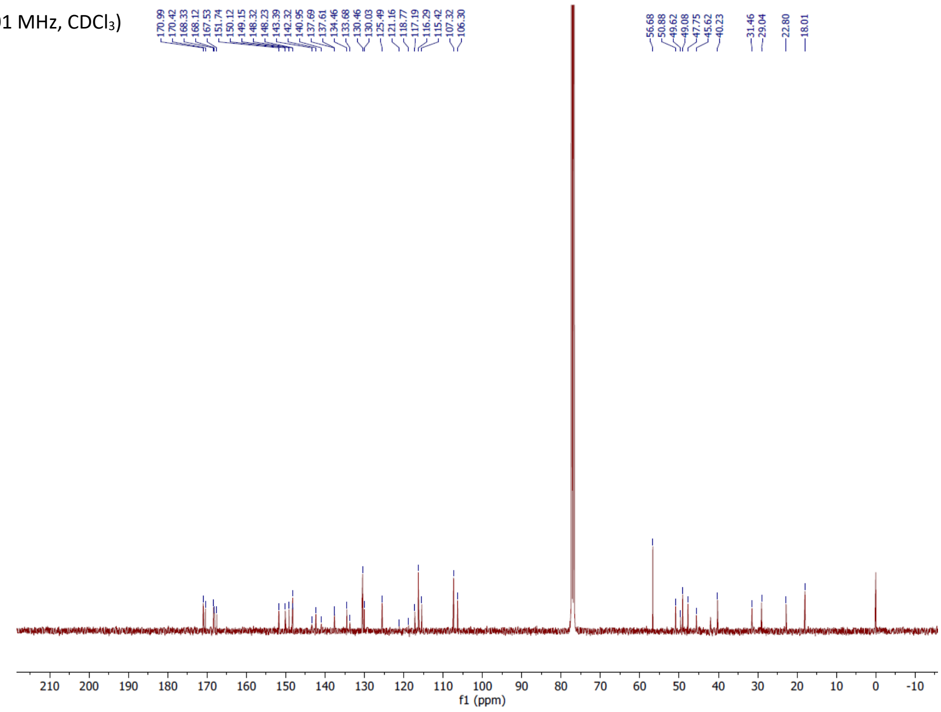

$^{19}F\{^{1}H\}$ NMR (376 MHz, $CDCl_3$)

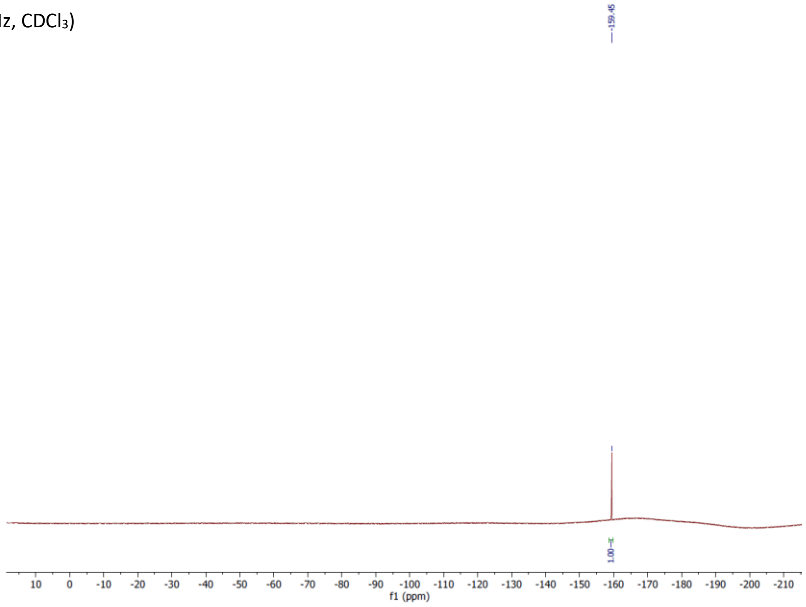

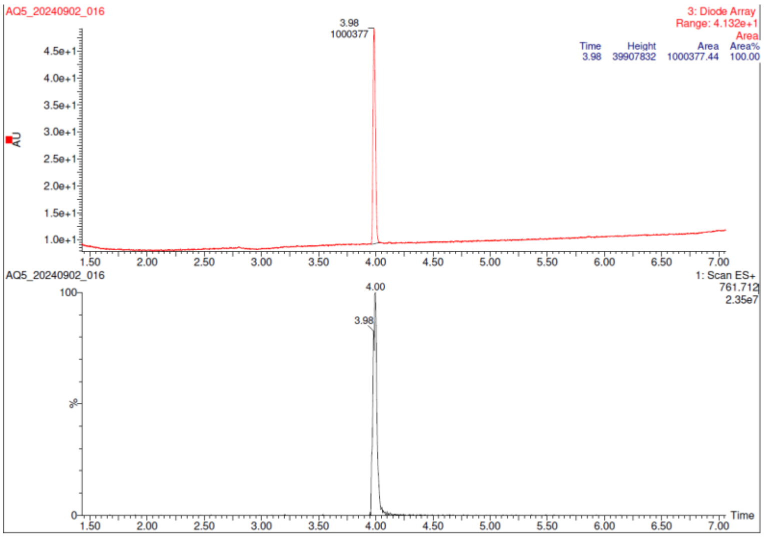

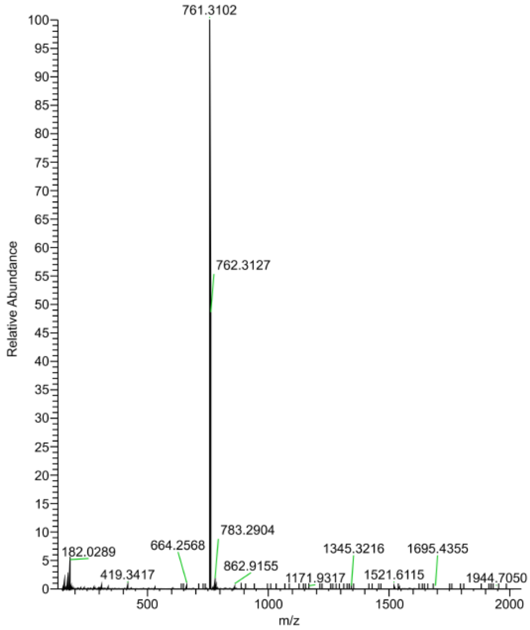

1-(5-(4-(4-(5-(4-Fluoro-3,5-dimethoxyphenyl)-4-methylpyridin-3-yl)phenyl)piperazine-1-carbonyl)-2-methoxyphenyl)dihydropyrimidine-2,4(1H,3H)-dione (**24**)

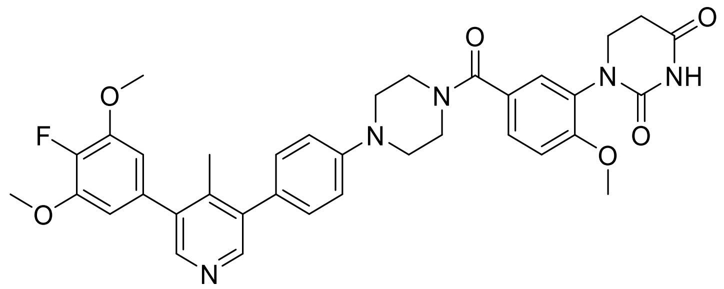

^{1}H NMR (500 MHz, $CDCl_3$)

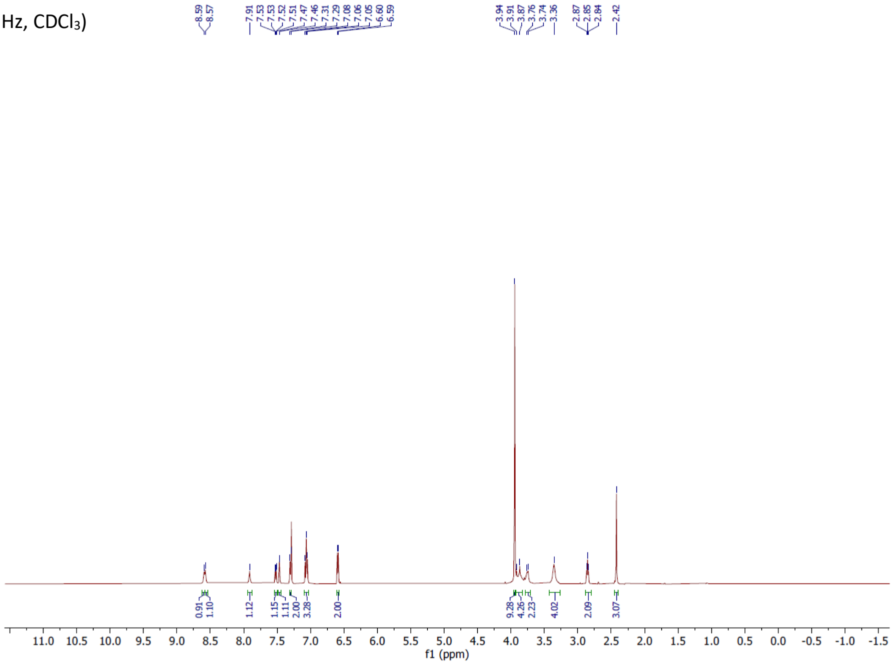

^{13}C NMR (126 MHz, $CDCl_3$)

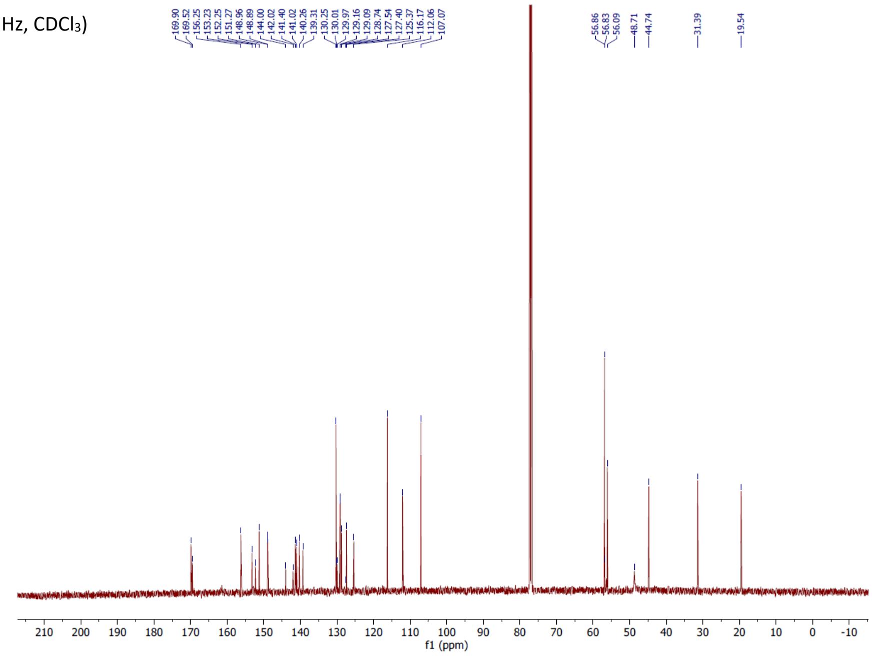

^{19}F NMR (471 MHz, $CDCl_3$)

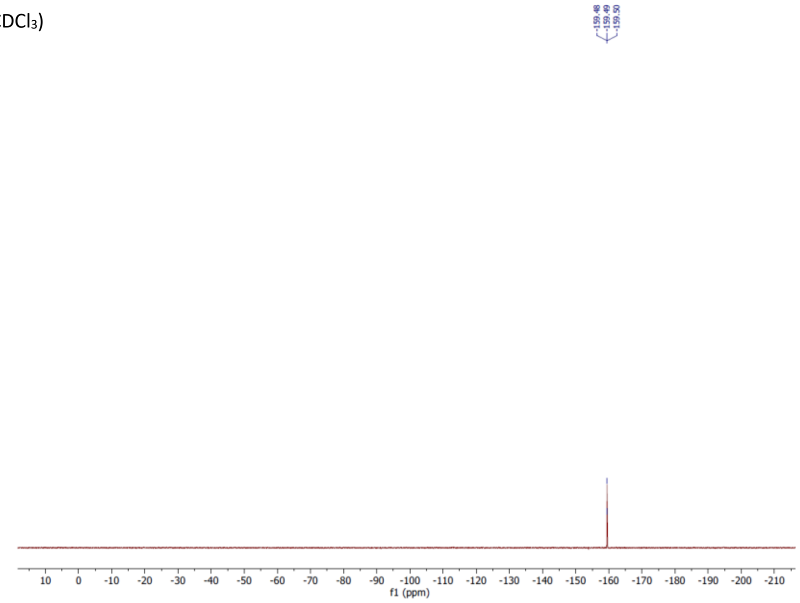

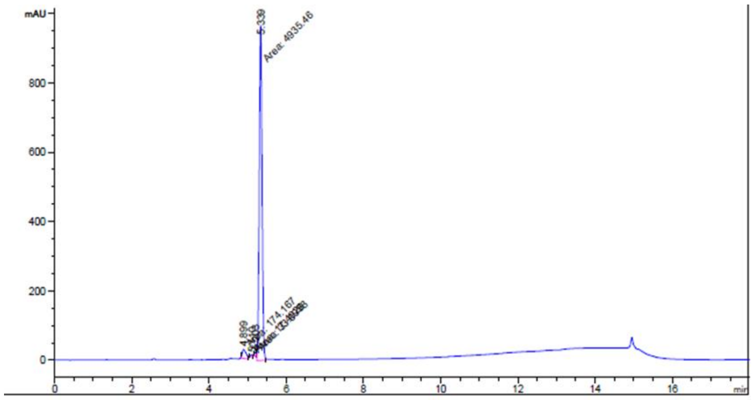

Signal 1: VWD1 A, Wavelength=250 nm

Peak #	RetTime [min]	Type	Width [min]	Area [mAU*s]	Height [mAU]	Area %
1	4.899	MM	0.1125	174.16684	25.80472	3.3748
2	5.110	MM	0.0570	17.49263	5.11535	0.3390
3	5.203	MM	0.0556	33.69981	10.09918	0.6530
4	5.339	MM	0.0849	4935.45557	968.37067	95.6333
Totals :				5160.81485	1009.38992	

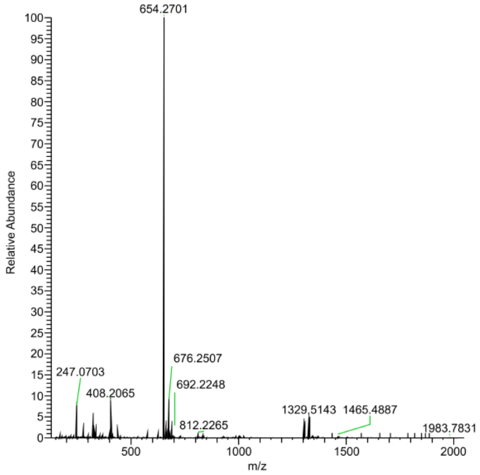

4. References

(1) Mohedas, A. H.; Xing, X.; Armstrong, K. A.; Bullock A. N.; Cuny, G. D.; Yu, P. B. Development of an ALK2-biased BMP type I receptor kinase inhibitor. *ACS Chem. Biol*. **2013**, *8*, 1291–1302.

(2) Yingling, J. M.; McMillen, W. T.; Yan, L.; Huang, H.; Sawyer, J. S.; Graff, J.; Clawson, D. K.; Britt, K. S.; Anderson, B. D.; Beight, D. W.; Desaiah, D.; Lahn, M. M.; Benhadji, K. A.; Lallena, M. J.; Holmgaard, R. B.; Xu, X.; Zhang, F.; Manro, J. R.; Iversen, P. W.; Iyer, C. V.; Brekken, R. A.; Kalos, M. D.; Driscoll, K. E. Preclinical assessment of galunisertib (LY2157299 monohydrate), a first-in-class transforming growth factor-β receptor type I inhibitor. *OncoTarget* **2018**, *9*, 6658-6667.
